# Metallaphosphinidene Coupling with a Phosphorus Ylide to Form a Phosphavinyl [P=CH_2_]^−^ Ligand

**DOI:** 10.1021/jacs.6c06304

**Published:** 2026-06-08

**Authors:** Mattias Tan, Christian Sandoval-Pauker, Zoltan Takacs, Anders Reinholdt

**Affiliations:** † Department of Chemistry, 5193 Lund University, Naturvetarvägen 22, Lund 22100, Sweden; ‡ Department of Chemical and Biomolecular Engineering, 3990 Rice University, 6100 Main Street, Houston, Texas 770052, United States

## Abstract

Metallaphosphinidenes, [M–P], contain open-shell single-atomic phosphorus but typically display uncontrollable reactivity, preventing their utilization to selectively construct elusive functional groups. Here, we report an iridium phosphaethynolate complex, [(PCP)­Ir­(PCO)] (**2**), in a halide metathesis with Na­(OCP). Photolysis of **2** leads to a bimetallic, side-on bound {P_2_} motif, [{(PCP)­(OC)­Ir}_2_(η^2^,η^2^;μ_2_-P_2_)] (**3**), via the intermediacy of a putative, triplet iridium phosphinidene, [(PCP)­Ir­(P)­(CO)] (**A**), probed computationally. When **2** is instead photolyzed in the presence of a phosphorus ylide, PhMe_2_PCH_2_, the photointermediate is intercepted, leading to a unique phosphavinyl complex, [(PCP)­Ir­(P=CH_2_)] (**4**), in 60% spectroscopic yield. Complex **2** also reacts thermally with PhMe_2_PCH_2_ to form **4**. Tracking of the extruded CO fragment uncovers a divergent reactivity landscape; in the photolytic pathway, a carbonyl complex, [(PCP)­Ir­(CO)]­(PCO), forms, whereas in the thermal pathway, one CO and two CH_2_ groups couple to a [C_3_] fragment in a new ylide, PhMe_2_PCHCOCH_3_. Structural characterization, isotopic labeling, and IR and NMR spectroscopic studies, along with quantum simulations, unveil a rigid, π-bonded [P=CH_2_]^−^ moiety in **4**, having magnetically inequivalent hydrogens at room temperature. Complex **4** comprises a deprotonated ligand form of the elusive phosphaethylene molecule (HP=CH_2_) but possesses a much lower isomerization barrier (15.9(5) kcal mol^–1^) than classical phosphaalkenes (>40 kcal mol^–1^), owing to an interplay between the [(PCP)­Ir]^+^ and [P=CH_2_]^−^ fragments, leading to a linear {Ir=P=CH_2_} transition geometry for this molecular switch. Lastly, we utilize the phosphavinyl ligand to form other rare π-constructs with an organic azide.

## Graphical abstract

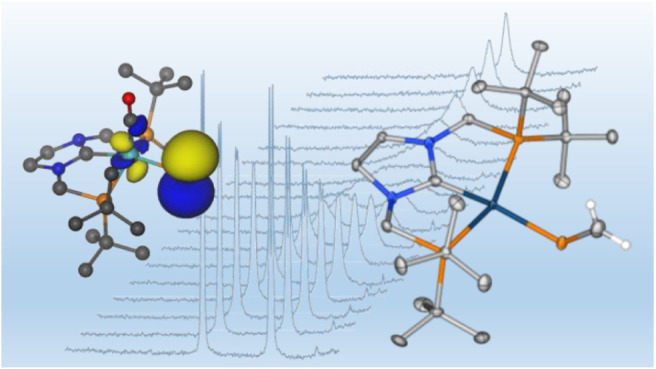

## Introduction

The short-lived and elusive phosphinidenide group (P^–^) has a subvalent^1^ electron configuration, which offers a donor–acceptor duality with enormous potential to install monatomic phosphorus in exotic bonding motifs. Substituted analogs of P^–^, such as organic phosphinidenes (PR), are extremely reactive in their free state and generally require cryogenic matrix isolation or transient spectroscopy to be observed.^2–5^ Being main-group-centered radicals,^6–13^ phosphinidenes are versatile intermediates with applications ranging from homogeneous catalysis,^14^ construction of conjugated polymers,^15^ C–P bond formation,^16^ small molecule activation,^17^ coordination chemistry,^18–22^ to group transfer reactivity.^23–35^ Considering the sterically exposed nature of an unsubstituted P^–^ moiety, this functionality is inherently reactive and difficult to handle. On the other hand, this also suggests it could have a synthetic impact on atom transfer processes. In 2016, Bertrand isolated a main-group, singlet phosphinidene and demonstrated its electrophilic coupling reactivity toward L-type ligands (Figure 1A).^36–38^ When a P^–^ group is instead bound to a transition metal, distinctive spin-states and reactivity result. Mindiola showed how a Ti^III^ phosphaethynolate extruded CO to form an electron-deficient, doublet titanium phosphinidene, [Ti^IV^≡P·], which dimerized to a side-on bound [Ti­(P_2_)­Ti] motif.^39^ Moreover, a growing number of studies have established how *d* ^8^ phosphaethynolate complexes may decarbonylate to form short-lived triplet metallaphosphinidene intermediates, [M–P], which dimerize to [M—P=P—M] zigzag motifs (Figure 1B).^40–43^ Given the transient existence of such electron-rich *d* ^8^ [M–P] intermediates, it would be desirable to kinetically outcompete their propensity for self-coupling and instead harness these reactive species to access phosphorus motifs that generally lack synthetic methodology of preparation. In one study, Powers showed that a triplet nickel phosphinidene abstracts hydrogen atoms from 1,3-cyclohexadiene (100 equiv) to form a phosphanide group, [PH_2_]^−^, in 20% yield (Figure 1C).^43^ Despite this encouraging result, strategies for creating more elaborate π-constructs remain scarce.

**Figure 1:**
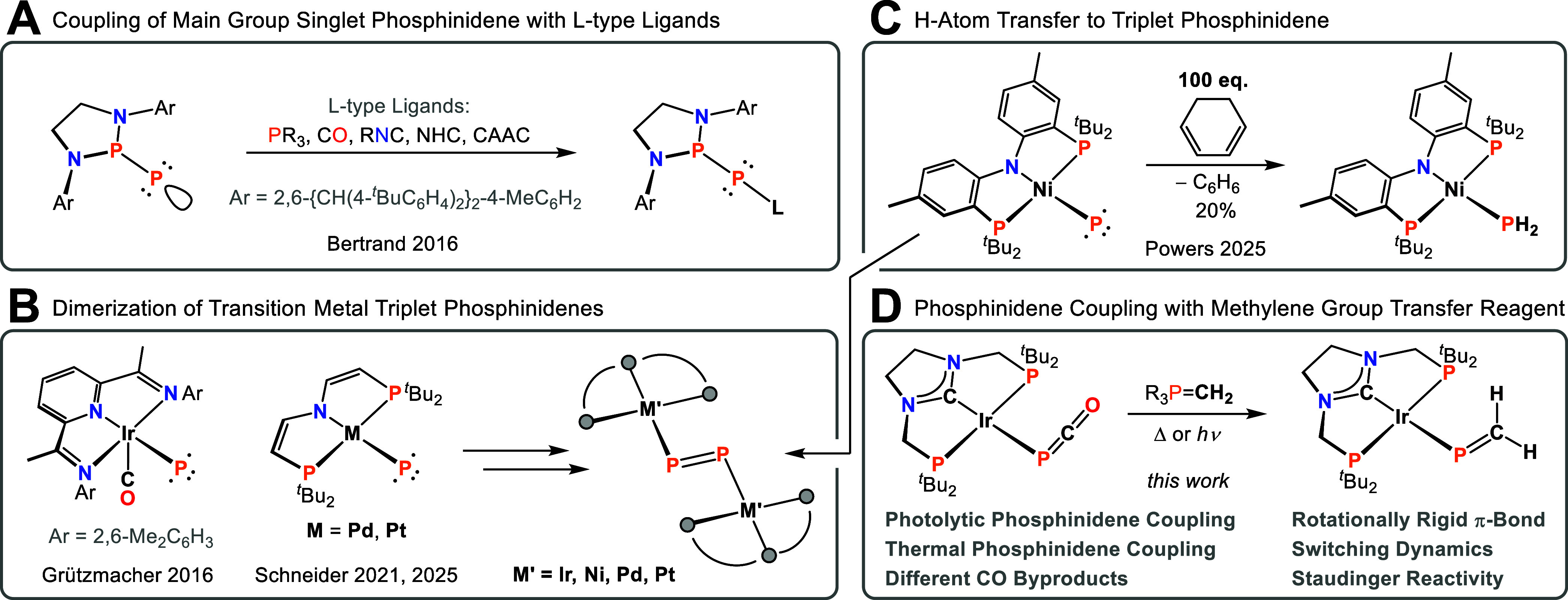
Selected examples of phosphinidene coupling reactions. A: Trapping of *p*-element singlet phosphinidene with L-type ligands. B: Dimerization of *d* ^8^ metal triplet phosphinidenes to form [P=P]^2–^ ligands, and C: H atom transfer to a *d* ^8^ Ni^II^ triplet phosphinidene to form [PH_2_]^−^ ligand. D: This work, demonstrating methylene group transfer to a putative triplet iridium phosphinidene to form a phosphavinyl complex.

One class of substances that could be advantageous to access from a phosphinidene coupling sequence is phosphaalkenes. The P=C double bond in a phosphaalkene is relatively weak compared to two P–C single bonds, making these unsaturated motifs susceptible to polymerization.^44–46^ To kinetically suppress oligomerization-type degradation pathways, phosphaalkenes have typically been isolated with sterically encumbering organic groups.^47–60^ In a similar fashion, distinctive classes of metallaphosphaalkenes have been pioneered by Weber,^61,62^ spanning phosphorus-bound (M—P=CR_2_, Type I)^63,64^ and carbon-bound (RP=CR—M, Type II)^65,66^ archetypes. Among all these phosphaalkene families, monosubstituted representatives, or even ones that are substituent-free, are scarce. Only two monosubstituted phosphaalkenes have been crystallographically authenticated to date, namely Appel’s supermesityl-bound [Mes*—P=CH_2_] system^67,68^ and Goicoechea’s extremely bulky arylbenzylamido-scaffolded [Ar*­(PhCH_2_)­N—P=CH_2_] phosphaalkene^69^ (Mes* = 2,4,6-^*t*^Bu_3_-C_6_H_2_, Ar* = 2,6-di­{3,5-di­(2,4,6-triisopropylphenyl)­phenyl}­phenyl). When looking to the simplest member of the phosphaalkene family, phosphaethylene (HP=CH_2_) is known to exist as a transient species in the gas phase,^70–73^ whereas the chemistry of this fundamental molecule, in neutral or deprotonated form, continues to elude experimental studies.

Herein, we report how a thermally stable iridium phosphaethynolate complex, [(PCP)­Ir­(PCO)], photolytically converts to a putative triplet metallaphosphinidene intermediate, [(PCP)­Ir­(P)­(CO)], which dimerizes to a bimetallic, side-on bound {P_2_} complex, [(PCP)­(OC)­Ir­(P_2_)­Ir­(CO)­(PCP)] (PCP = 1,3-bis­(di-*tert*-butylphosphinomethyl)-4,5-dihydroimidazol-2-ylidene). When [(PCP)­Ir­(PCO)] is instead photolyzed in the presence of a phosphorus ylide, PhMe_2_PCH_2_, coupling of the putative metallaphosphinidene with a methylene fragment produces an unsubstituted and room-temperature-stable phosphavinyl motif, [(PCP)­Ir­(P=CH_2_)], along with an iridium carbonyl complex (Figure 1D). In addition to the photochemical route, we also identify an alternate thermal coupling route, in which a series of C–C bond-forming steps lead to [(PCP)­Ir­(P=CH_2_)] along with a conjugated carbonyl ylide, PhMe_2_PCHCOCH_3_, which possesses a [C_3_] fragment derived from one CO and two CH_2_ groups. We scrutinize the π-bonded [(PCP)­Ir­(P=CH_2_)] complex through X-ray diffraction, isotopic labeling, IR spectroscopy, multinuclear NMR spectroscopy, and theoretical studies. Strikingly, a rigid P=C double bond gives rise to two magnetically distinct hydrogen environments for the [P=CH_2_]^−^ ligand at room temperature. The hydrogens interchange at elevated temperatures, and we probe this degenerate dynamic by variable-temperature NMR studies and quantum chemical simulations. By traversing a linear transition geometry, {Ir=P=CH_2_}, switching proceeds with a Gibbs’ free energy of activation (16 kcal mol^–1^) well below the rotational barrier for a typical phosphaalkene. Finally, we demonstrate the utility of the [P=CH_2_]^−^ ligand to form other π-conjugated motifs in its reaction with an organic azide.

## Results and Discussion

### Isolation of an [Ir–PCO] Complex (**2**)

Over the past decade, Na­(OCP) has gained traction as a versatile P atom transfer reagent, serving as the basis for myriads of main-group and transition metal functionalities.^74–107^ To obtain a platform suitable for accessing metallaphosphinidene reactivity, we treated [Ir­(cod)­Cl]_2_ with the fully saturated pincer ligand precursor, H_2_(PCP),^108^ under an atmosphere of H_2_ (Scheme 1). This protocol afforded cyclooctane along with pincer complex [(PCP)­IrCl] (**1**), isolated as a yellow crystalline material in 84% yield. Subsequent treatment of chloride precursor **1** with Na(OCP)·2.5 dioxane in THF over 16 h afforded the orange phosphaethynolate complex [(PCP)­Ir­(PCO)] (**2**) in 98% yield after removal of NaCl and volatiles (Figure 2). An X-ray crystallographic study revealed a phosphorus-bound PCO^–^ ligand coordinated at a right angle to a square planar iridium center. The presence of a phosphaethynolate ligand was further verified by a very strong IR resonance (1841 cm^–1^; antisymmetrical PCO mode), falling at higher energy than observed for typical salts containing a discrete PCO^–^ anion (1757 to 1772 cm^–1^).^109^ A comparison between the fingerprint regions of **1** and **2** also identified a symmetrical PCO mode at 1092 cm^–1^. Finally, NMR spectroscopic studies revealed a highly shielded ^31^P resonance (−355 ppm) as well as a ^13^C doublet (178 ppm, ^1^ *J*_CP_ = 77 Hz). These spectral data are in accord with representative data for the PCO^–^ anion.^75^

**Scheme 1:**
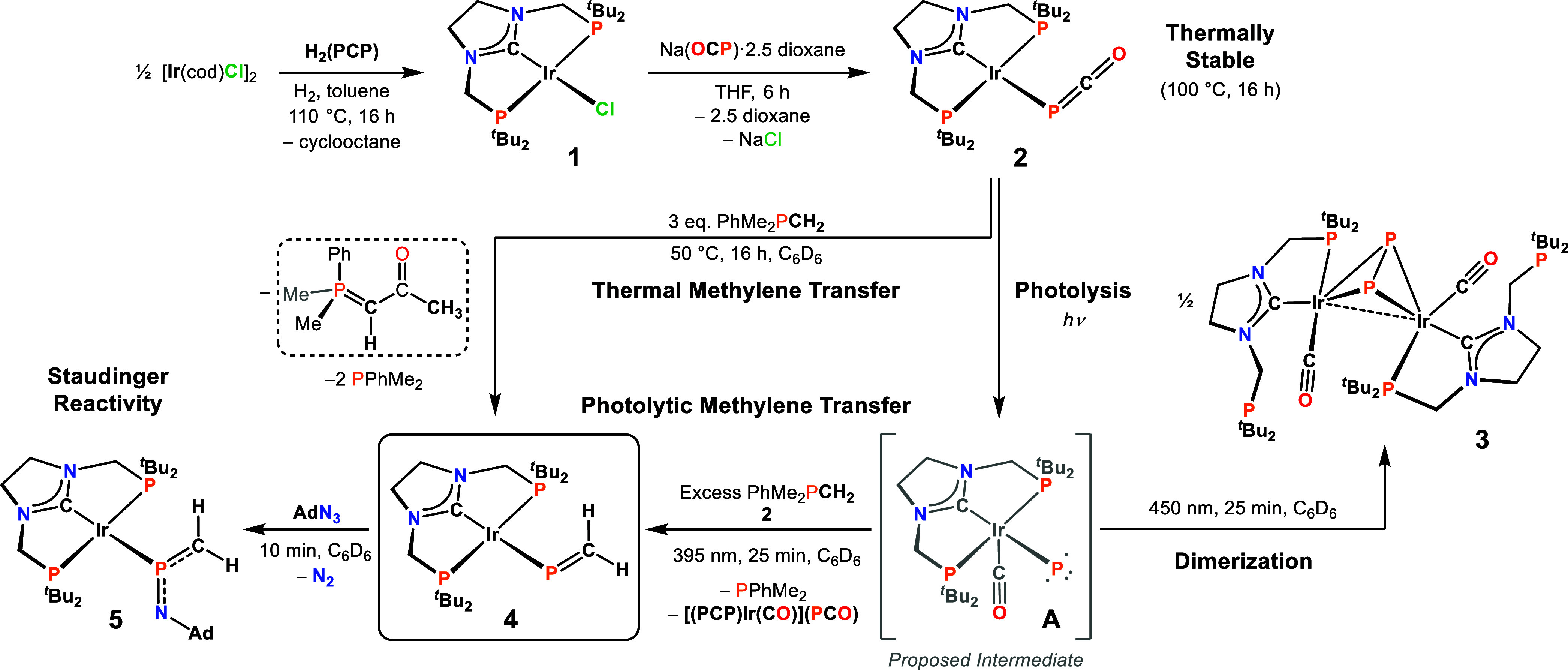
Precursor [(PCP)­IrCl] (**1**) Reacts with Na­(OCP) to Form Thermally Stable [(PCP)­Ir­(PCO)] (**2**); Photolysis of **2** Forms a Putative Triplet Phosphinidene [(PCP)­Ir­(P)­(CO)] (**A**), which Dimerizes to Form a Bimetallic and Side-On Bound {P_2_} Motif, [{(PCP)­(OC)­Ir}_2_(η^2^,η^2^;μ_2_-P_2_)] (**3**); Methylene Group Transfer from PhMe_2_PCH_2_ Traps Putative **A** to Assemble a Phosphavinyl Ligand, [(PCP)­Ir­(P=CH_2_)] (**4**); Staudinger-Type Reactivity of **4** with AdN_3_ Forms a Planar and π-Delocalized Ligand Motif, [(PCP)­Ir­{P­(=CH_2_)­(NAd)}] (**5**)

**Figure 2:**
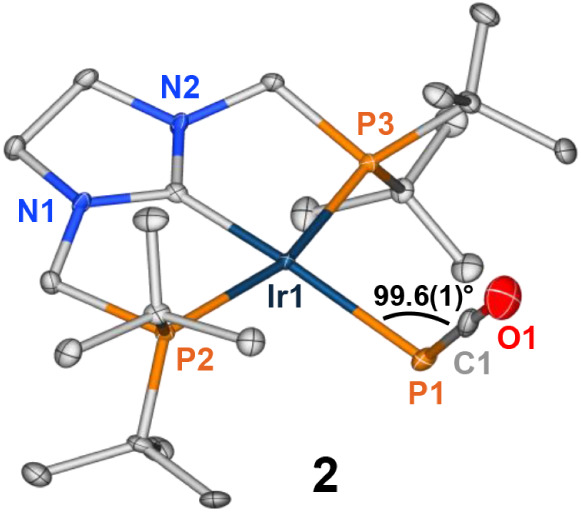
X-ray crystallographic structure of **2**, recorded at 100(2) K. Thermal ellipsoids are at 50% probability, and H atoms are omitted.

### Photolysis of [Ir–PCO] Complex **2** Generates a Bimetallic and Side-On Bound {P_2_} Ligand Motif, [Ir­(P_2_)­Ir] (**3**)

When kept in the dark, solutions of **2** are thermally stable (100 °C, 16 h), whereas the complex decomposes upon exposure to light. Photolysis in benzene (450 nm, LED lamp, 30 W, 25 min) generated a multicomponent reaction mixture, from which a bimetallic complex with a side-on bound {P_2_} ligand, [{(PCP)­(OC)­Ir}_2_(η^2^,η^2^;μ_2_-P_2_)] (**3**) was isolated in low yield (Scheme 1). X-ray diffraction revealed a tetrahedral {Ir_2_P_2_} core retaining a carbonyl ligand on each metal center, a noncoordinated phosphine group on each pincer ligand, as well as a P–P linkage (Figure 3). The Ir····Ir separation is 2.858(1) Å, and a topological analysis (DFT at the PBE0-D3/def2-TZVP­(-f) level of theory) revealed a bond critical point along the Ir····Ir path of a (3, −1) type, indicating an attractive bonding interaction with partial covalent character. Furthermore, there is no symmetry element relating the two {(PCP)­Ir­(CO)} moieties; for example, the noncoordinated PCP arms point in opposite directions from the {P_2_} ligand. The side-on bound {P_2_} ligand contrasts with the apparent tendency for *d* ^8^ phosphinidene dimerization products to assume zigzag [M—P=P—M] geometries.^40–43^ Spectral fingerprints of **3** were further established by diagnostic ^31^P NMR resonances for the noncoordinated pincer arm (17 ppm, singlet), the iridium-bound pincer arms (44 ppm, two overlapping multiplets), and two distinct chemical environments for the {P_2_} ligand (−110 and −150 ppm, both with ^1^ *J*_P–P_ = 443 Hz). Unlike zigzag disphosphene [M—P=P—M] motifs, which display strongly deshielded ^31^P NMR shifts (>650 ppm),^40–43^ the tetrahedral structure in **3** resulted in a much more shielded ^31^P NMR environment. However, these spectroscopic signatures from **3** are entirely in line with literature values for ^31^P chemical shifts and coupling constants in diphosphadimetallatetrahedranes.^110,111^ Finally, complex **3** displays two carbonyl IR stretching modes at 1960 cm^–1^ and 1880 cm^–1^, indicating a quite electron-rich bimetallic core.

**Figure 3:**
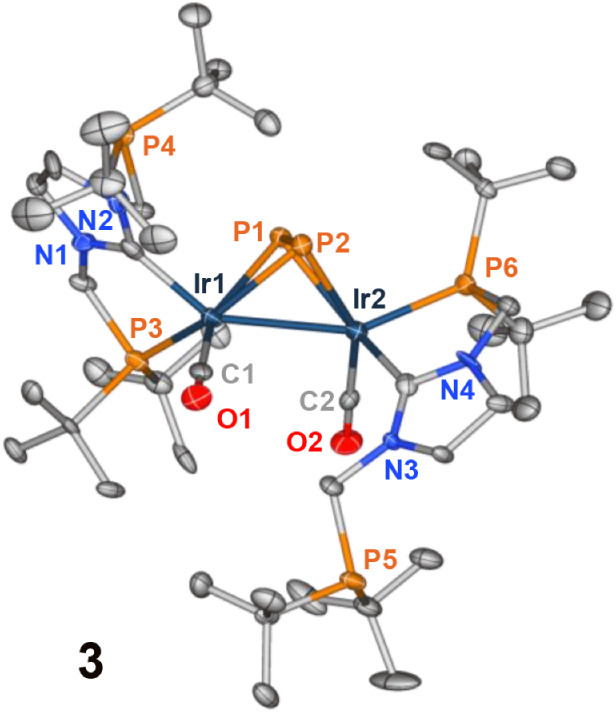
X-ray crystallographic structure of **3**, recorded at 100(2) K. Thermal ellipsoids are at 50% probability, and H atoms are omitted.

### Electronic Structure of a Putative Phosphinidene Intermediate (**A**)

Schneider^42^ and Powers^43^ recently scrutinized how *d* ^8^ phosphaethynolate complexes may extrude CO to form transient [M–P] phosphinidenes, through spectroscopic, magnetic, and theoretical means, and also studied how these triplet species dimerize to form diphosphene complexes. To address the electronic structure of likely intermediates leading to **3**, we turned to theoretical studies using DFT at the PBE0-D3/def2-TZVP­(-f) level of theory for geometry optimizations and TPSSh-D3/def2-TZVP for single-point calculations. Starting from **2**, initial breaking of the P–CO bond concomitant with migration of the CO group onto iridium would generate a five-coordinate phosphinidene intermediate, [(PCP)­Ir­(P)­(CO)] (**A**), which could subsequently expel CO to form a four-coordinate phosphinidene [(PCP)­Ir­(P)] (**B**). By evaluating the relative energetics of these putative intermediates, triplet **A** turned out to be most stable (8.3 kcal mol^–1^ lower in energy than singlet **A**, Supporting Information, Table S12). The two spin-states of **B** were found to be practically isoenergetic (to within 2.1 kcal mol^–1^), but more than 12 kcal mol^–1^ higher in energy than triplet **A**. In accord with the isolation of a {P_2_} coupling product such as **3**, the SOMOs of **A** essentially correspond to phosphorus-based 3*p* orbitals oriented perpendicular to the Ir–P interatomic axis (Figure S77).

### Photolysis of **2** in the Presence of a Phosphorus Ylide Generates a Phosphavinyl Complex (**4**)

Considering the high reactivity of an open-shell species such as the hypothetical triplet iridium phosphinidene **A**, we inquired if such a putative photointermediate could be trapped using a phosphorus ylide to transfer a methylene group. In a previous study, Appel found that a supermesityl phosphaketene, [Mes*–PCO], would thermally substitute its oxygen group in a Wittig-type conversion with the phosphorus ylide, Ph_3_PCPh_2_, to form a phosphorus allene, [Mes*—P=C=CPh_2_].^112^ However, in our case, strikingly different group transfer reactivity proceeded. Upon dissolving **2** and Ph_3_PCH_2_ in C_6_D_6_ at room temperature, no immediate reaction occurred, but when irradiating the orange reaction mixture (395 nm, 1.3 W, 25 min), a dark red phosphavinyl complex, [(PCP)Ir(P=CH_2_)] (**4**, Figure 4A), formed in low yield along with significant amounts of other decomposition products. When turning to a more sterically accessible phosphorus ylide such as PhMe_2_PCH_2_, a more selective photolytic conversion ensued to form **4** as the major phosphorus-containing product, along with several different byproducts (395 nm, LED, 1.3 W, 25 min, Scheme 1). Optimal conversion of **2** to **4** required an excess of PhMe_2_PCH_2_, leading to 60% spectroscopic yields of **4** (when using 3 equiv PhMe_2_PCH_2_). The irradiation time must be kept as brief as possible to prevent the phosphavinyl product from decaying to poorly defined decomposition products. From the photolytic conversion, an iridium salt, [(PCP)­Ir­(CO)]­(PCO), was also isolated, indicating the fate of photoreleased CO. This carbonyl complex was identified from a combination of X-ray diffraction data (Figure S71), as well as characteristic ^31^P NMR resonances (81.2, −391.5 ppm; PCP, PCO^–^), and IR stretching frequencies (1942, 1966 cm^–1^; CO, PCO^–^).

**Figure 4:**
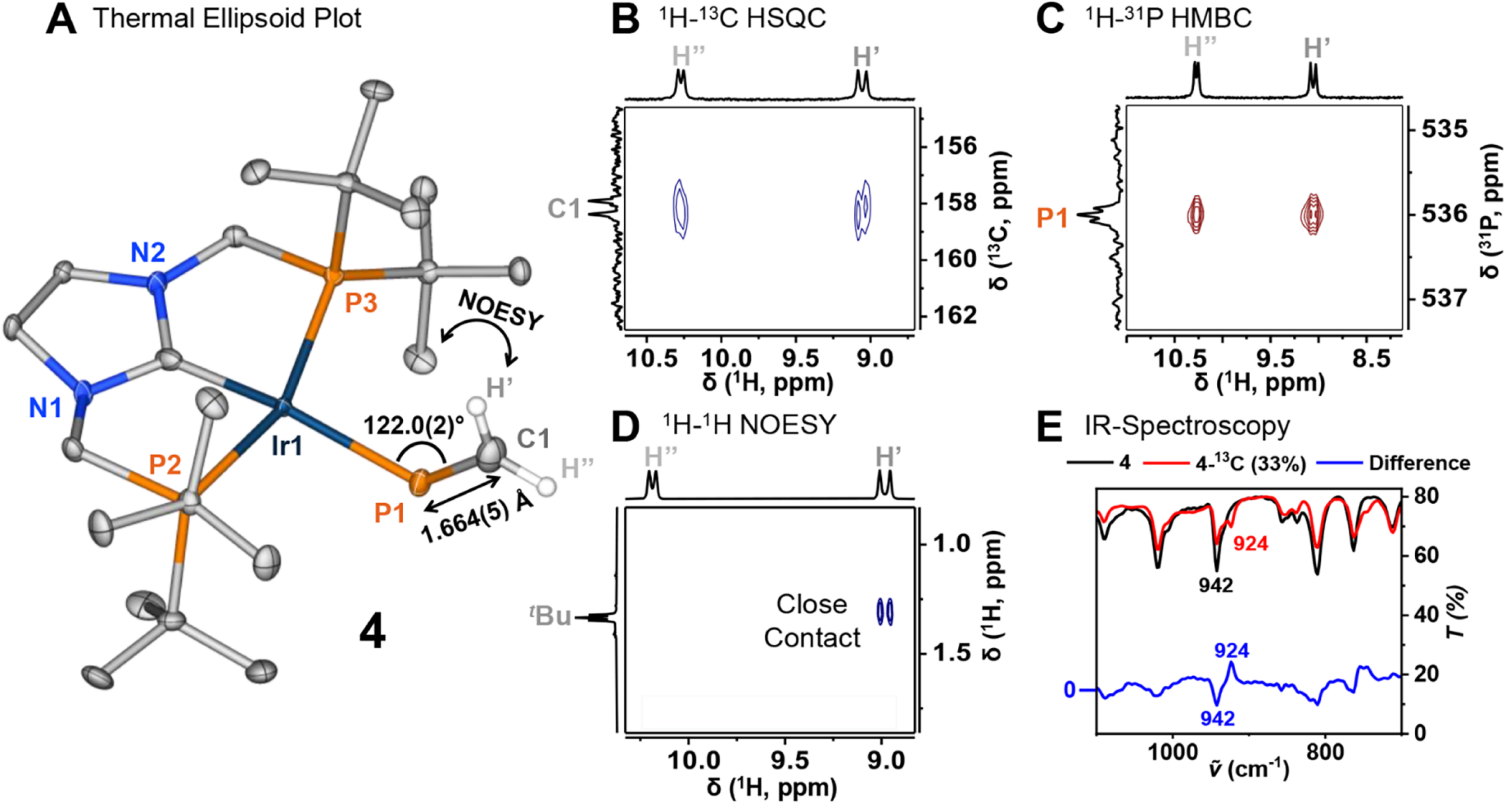
A: X-ray crystallographic structure of **4**, recorded at 100(2) K. Thermal ellipsoids are at 50% probability, and H atoms are omitted (except H′ and H″). B: ^1^H–^13^C HSQC correlating H′, H″, and C1 of the [P=CH_2_]^−^ ligand. C: ^1^H–^31^P HMBC correlating H′, H″, and P1 of the [P=CH_2_]^−^ ligand. D: ^1^H NOESY showing a close proximity between H′ and the *^t^* Bu groups of the PCP ligand. E: IR spectral data of **4** and the 33% ^13^C-enriched isotopologue **4-^13^C**, identifying the P=C stretching frequency.

### Thermal Conversion of **2** with PhMe_2_PCH_2_ Generates Phosphavinyl Complex **4** along with a New Phosphorus Ylide

When monitoring reaction mixtures containing **2** and PhMe_2_PCH_2_, slow formation of **4** proceeded (4%, 1 h, 25 °C), even when the mixtures were kept in the dark. Upon heating (50 °C, 16 h), the phosphavinyl complex **4** formed in a highly selective transformation. Interestingly, it was necessary to employ three equivalents of PhMe_2_PCH_2_ to achieve full thermal conversion of **2** to **4**. In accord, we observed free PhMe_2_P (2 equiv) but also isolated 1 equiv of an acetylmethylene ylide, PhMe_2_PCHCOCH_3_, from the reaction mixtures. The structure of the new ylide was confirmed in an X-ray crystallographic study, and the CH and CH_3_ groups in PhMe_2_PCHCOCH_3_ were shown to retain a ^13^C-label, when a ^13^C-enriched PhMe_2_P^13^CH_2_ reagent was employed, demonstrating the [C_3_] chain to derive from the [C_1_] fragments, CO and CH_2_ (Supporting Figures S42–S44).

### Mechanistic Differences between Photolytic and Thermal Routes to Phosphavinyl Complex **4**

To gain more insight into the mechanistic pathways leading from **2** to **4**, we conducted a series of control experiments. In case of the thermal pathway, we exposed a C_6_D_6_ solution of PhMe_2_PCH_2_ to CO (1 bar). No formation of PhMe_2_PCHCOCH_3_ was observed at room temperature or at 70 °C over 16 h, and iridium complexes such as **2** and **4** did not catalyze the reaction between CO and PhMe_2_PCH_2_. Since free CO fails to react with PhMe_2_PCH_2_, it is unlikely that the thermal pathway leading from **2** to **4** begins with cleavage of the P–CO bond of the phosphaethynolate ligand, to form a putative iridium phosphinidene intermediate, followed by release of CO. On the other hand, a mechanism with an initial C–C bond forming step between the phosphaethynolate (**2**) and ylide (PhMe_2_PCH_2_) carbons, followed by additional methylene and proton transfer steps, is a plausible scenario to form the byproduct, PhMe_2_PCHCOCH_3_. Along these lines, Stephan recently reported how a germanium phosphaethynolate, [Ph_3_Ge–PCO], reacts with Ph_3_PCH_2_, to form a C–C coupled intermediate, [Ph_3_GeP­(C=O)­CH_2_PPh_3_], which tautomerizes to form [Ph_3_GePH­(C=O)­CHPPh_3_].^113^ We next turned attention to the photolytic pathway. When analyzing the byproducts formed during the photolysis of **2** and PhMe_2_PCH_2_, we observed only minor amounts of PhMe_2_PCHCOCH_3_ (∼10% spectroscopic yield, Supporting Figure S35). Considering the slow thermal conversion of **2** to **4** at room temperature, the observed PhMe_2_PCHCOCH_3_ from photolysis is likely due to such a background reaction. More importantly, since the amount of the [C_3_] product is too low to account for the amount of phosphavinyl complex formed (60%), the elementary steps in the photolytic conversion must be mechanistically distinct from those in the thermal conversion. Thus, an initial C–C bond forming step between the phosphaethynolate carbon (**2**) and ylide carbon (PhMe_2_PCH_2_) is unlikely in the photolytic route. On the other hand, an iridium phosphinidene intermediate akin to the hypothetical species **A** could trap a methylene group and form **4**. Subsequent release of CO could then explain why a complex salt such as [(PCP)­Ir­(CO)]­(PCO) is observed in the photolytic route.

### Structural and Spectroscopic Studies of Phosphavinyl Complex **4**

To remove the thermal byproducts, PhMe_2_P and PhMe_2_PCHCOCH_3_, complex **4** was fractionally crystallized from Et_2_O in 46% isolated yield. Analysis of the dark red crystals by X-ray crystallography revealed a slightly puckered, square planar iridium center coordinated by a P-bound phosphavinyl [P=CH_2_]^−^ ligand (Figure 4A). The presence of a double bond was indicated by a short P=C distance (1.664(5) Å), in close agreement with the bond distance for free HP=CH_2_ in the gas phase (1.673(2) Å).^70^ In keeping with this description, the [P=CH_2_]^−^ ligand displays nonlinear coordination to iridium, 122.0(2)°, whereas its two hydrogen atoms were located in the Fourier difference map, indicating a planar, *sp* ^2^-hybridized carbon atom.

We then went on to characterize the [P=CH_2_]^−^ multiple-bonded ligand fragment by spectroscopic means. First and foremost, the ^1^H NMR spectrum of **4** displays two deshielded doublets at 9.06 and 10.27 ppm (^2^ *J*_HP_ = 28 and 16 Hz), suggesting restricted rotation about the P=C double bond at 298 K. These resonances further displayed correlations to a carbon at 158 ppm (^1^H–^13^C HSQC, Figure 4B) and a very deshielded phosphorus nucleus at 536 ppm (^1^H–^31^P HMBC, Figure 4C), reaffirming the overall connectivity within the [P=CH_2_]^−^ ligand. Whereas phosphaethynolate precursor **2** displays a one-bond coupling constant of ^1^ *J*_PC_ = 77 Hz, this interaction is diminished for the phosphavinyl ligand in **4** (^1^ *J*_PC_ = 60 Hz), suggesting a decrease in *s*-character between the respective PC bonds. By conducting a NOESY experiment, we could further assign one hydrogen (9.06 ppm) as being in closer through-space proximity to the ^*t*^Bu groups of the pincer ligand, therefore defining the orientation of the [P=CH_2_]^−^ ligand (Figure 4D). Finally, by preparing a 33% ^13^C-enriched sample of **4** (denoted **4-^13^C**), and comparing the IR-spectral data for the ^12^C and ^13^C isotopologues (Figure 4E), we observed a resonance at 942 cm^–1^ (**4**) shift to 924 cm^–1^ (**4-^13^C**), corresponding to a lower vibrational energy as compared to the {P=C} stretching mode in parent HP=CH_2_ (1012 cm^–1^).^114^

### Electronic Structure of **4**

Next, we probed the electronic structure of **4** by DFT, using PBE0-D3/def2-TZVP­(-f) for geometry optimizations and TPSSh-D3/def2-TZVP for single-point calculations. Among the frontier orbitals of **4** (Figure S83), the most telling interaction is the HOMO–2, which depicts a P=C π-bond, arising from side-one overlap between carbon 2*p* and phosphorus 3*p* orbitals. In accord with this type of π-manifold, calculated Mayer (1.828) and Wiberg (2.264) bond orders indicate double-bond character for the phosphavinyl ligand. This description is further corroborated by a topological analysis using the quantum theory of atoms in molecules (QTAIM), which shows substantial ellipticity (ε = 0.3159), consistent with the π-bonding electrons being concentrated perpendicular to the [P=CH_2_]^−^ plane.

Moreover, in order to elucidate the origin of the deshielded ^31^P environment for the phosphavinyl ligand, we found that the HOMO of **4** essentially portrays a phosphorus-based lonepair donating to iridium (σ_Ir–P_), while the LUMO+1 represents a vacant π*_P=C_ orbital. This *n*(P)-π* frontier orbital sequence of the metallaphosphaalkene contrasts with the π–π* sequence expected for a normal phosphaalkene. The resulting HOMO–LUMO gap is therefore relatively low, and the NMR shielding tensor of **4** is dominated by a strong contribution from the paramagnetic term, leading to a high value for the ^31^P chemical shift (Table S16).^61,62^

### Interconversion Dynamics for the [P=CH_2_]^−^ Group

Considering the interest in rigid P=C double bonds for molecular switching,^115,116^ and given the spectroscopic and computational evidence for a [P=CH_2_]^−^ moiety in **4**, we set out to probe if its two hydrogen environments (denoted H′ and H″) could thermally interconvert. While rotational barriers for free phosphaalkene double bonds have been well studied,^115–117^ in our case, the interplay with iridium 5*d* orbitals opens distinctive mechanistic scenarios (Figure 5A). First of all, the CH_2_ group could rotate, resulting in breakage of the [P=CH_2_]^−^ π-bond and interchange of H′ and H″ (Scenario A). This scenario was calculated to have a prohibitive activation barrier (>50 kcal mol^–1^), in keeping with typical rotational barriers for phosphaalkenes (>40 kcal mol^–1^).^117^ Second, the [P=CH_2_]^−^ ligand could change coordination mode from being essentially a σ-coordinated ligand to being a side-on, π-bound moiety,^118–120^ resulting in H′ and H″ being momentarily indistinguishable (Scenario B). This scenario also possesses a relatively high interconversion barrier (21.3 kcal mol^–1^). Third, the [P=CH_2_]^−^ group could undergo a bending vibration, traversing a linear, 18-electron transition geometry with heteroallenic {Ir=P=CH_2_} bonding^121^ (Scenario C). This scenario was calculated to be thermally more accessible (15.5 kcal mol^–1^).

**Figure 5:**
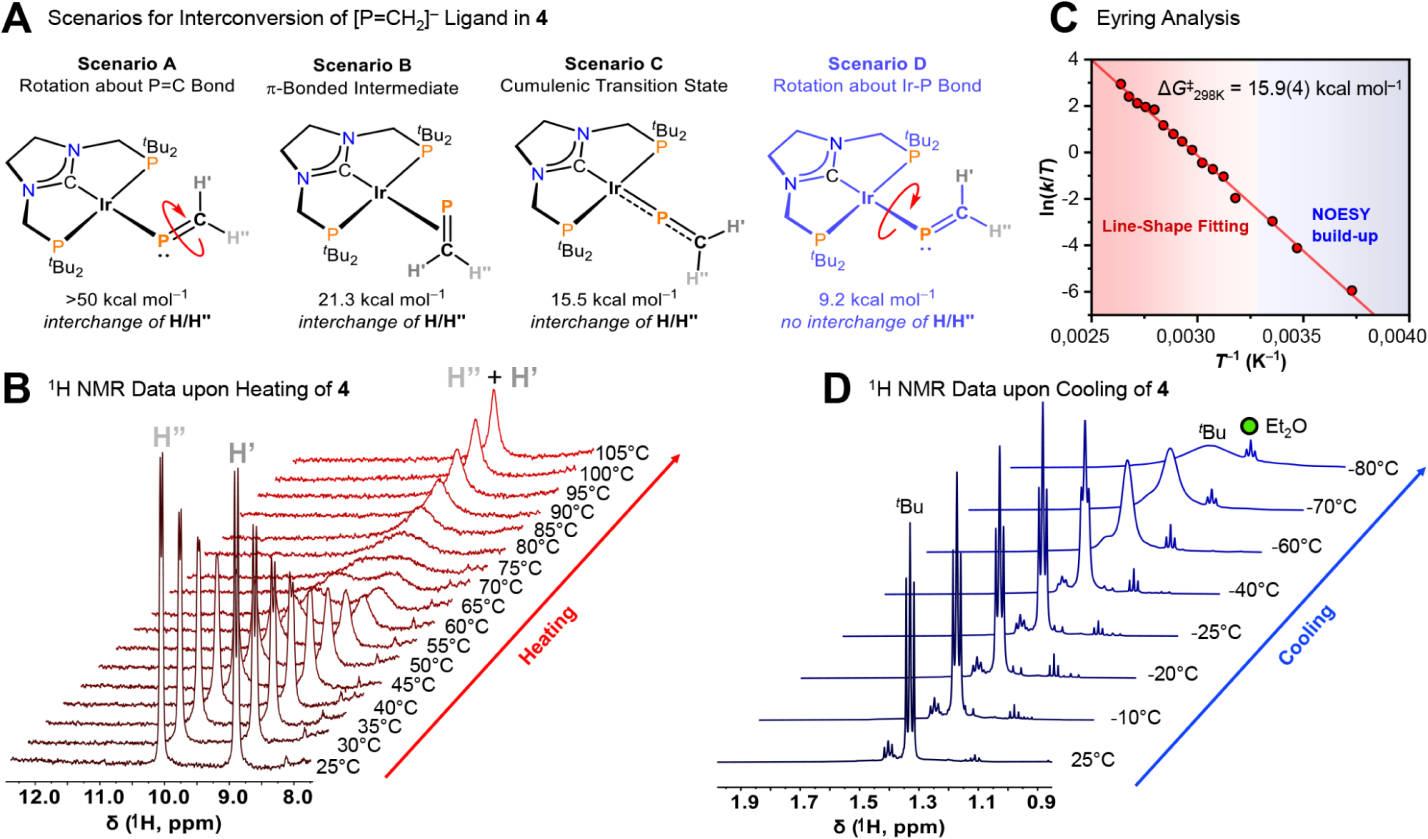
Solution dynamics for **4**. A: Scenarios for interconversion of the protons within the [P=CH_2_]^−^ ligand. Rotation about [P=CH_2_]^−^ π-bond (Scenario A). Formation of a side-on, π-bonded [P=CH_2_]^−^ ligand (Scenario B). Bending vibration traversing heteroallenic transition state (Scenario C). Rotation about Ir–P σ-bond (Scenario D). B: Variable temperature ^1^H NMR studies (25 to 105 °C) showing coalescence of [P=CH_2_]^−^ protons. C: Eyring analysis for variable temperature studies at −5 to +105 °C. D: Variable temperature ^1^H NMR studies (+25 °C to −80 °C) showing broadening of *^t^* Bu resonances.

We then tracked the spectral features of **4** (toluene-*d* ^8^) at 5 K intervals between 25 and 105 °C, focusing on the ^1^H NMR resonances from H′ and H″ (Figure 5B). During this experiment, the spectra developed from showing two sharp doublets (25 °C), to two separate but broadened peaks, followed by a broad coalescing feature, to finally showing a single ^1^H resonance for the [P=CH_2_]^−^ ligand (105 °C). On cooling the sample back to 25 °C, the two separate doublets reappeared in a fully reversible fashion. By conducting a line-width analysis (+41 to +105 °C), complemented with NOESY buildup experiments (−5 to +25 °C), we could extract rate constants for the underlying interconversion process over a temperature interval of approximately 110 K. A subsequent Eyring analysis (Figure 5C) quantified the activation enthalpy Δ*H* ^‡^ = 16.3(3) kcal mol^–1^ and activation entropy Δ*S* ^‡^ = 1.6(8) cal mol^–1^ K^–1^ for the degenerate interconversion process. The near-zero magnitude of the activation entropy suggests an ordered transition state, consistent with a unimolecular process. Furthermore, the activation Gibbs free energy, Δ*G* ^‡^_298 K_ = 15.9(4) kcal mol^–1^, is in excellent agreement with the computationally determined barrier for Scenario C, lending experimental support for an interconversion mechanism traversing a linear {Ir=P=CH_2_} transition geometry.

Finally, we note that a fourth interconversion process, involving rotation about the Ir–PCH_2_ σ-bond could be imagined (Scenario D). In this scenario, H′ constantly remains in closer proximity to the iridium center than H″, and so, these hydrogens do not interchange as a result of the rotation. Nevertheless, when cooling a sample of **4**, the ^1^H NMR signature from the ^*t*^Bu groups of the pincer ligand (one sharp triplet at room temperature) drastically broadened (fwhm = 110 Hz at −80 °C, Figure 5D). It should be noted that a triplet peak arising from a trace of diethyl ether remained sharp throughout this cooling process. The broadening reflects that the ^*t*^Bu groups go from experiencing rapid rotation to experiencing restricted rotation of the neighboring [P=CH_2_]^−^ group, effectively lowering the apparent spectroscopic symmetry of the whole molecule from *C*_2*v*_ to *C*_*s*_. In accord, computational modeling revealed a low rotational barrier for the Ir–PCH_2_ σ-bond (9.2 kcal mol^–1^).

### Oxidation of [P=CH_2_]^−^ Complex **4** with an Azide Produces a π-Conjugated [P­(=CH_2_)­(NAd)]^−^ Ligand

To probe the synthetic utility of the [P=CH_2_]^−^ fragment in **4**, we exposed the complex to an azide. When treated with 1-adamantyl azide (AdN_3_) in C_6_D_6_, evolution of N_2_ ensued over 10 min, and the color of the reaction mixture changed from orange to red. Crystallization from pentane afforded a Staudinger-type product, [(PCP)­Ir­{P­(=CH_2_)­(NAd)}] (**5**) in 97% isolated yield. X-ray diffraction revealed an essentially planar [P­(=CH_2_)­(NAd)]^−^ ligand (angle sum about P1 = 359.9°) oriented perpendicular to a square planar iridium node (Figure 6A). The Fourier difference map further located the hydrogen atoms of the CH_2_ group within the plane spanned by the central {NPC} atoms (Figure 6B). The imino­(methylene)­phosphorane motif in **5**, having only one organic group appended, is an exceedingly rare coordination motif.^122^ Niecke reported a more crowded, disubstituted organic analog, [(Me_3_Si)_3_C–P­(=CH_2_)­(NMes*)],^123^ and this P^V^ system possesses a short P=C linkage (1.612(3) Å) compared to our system **5** (1.684(5) Å). Considering the electropositive nature of the iridium­(I) center, complex **5** is instead describable as containing a π-conjugated P^III^ ligand, in line with its comparatively long P=C bond. Salient spectroscopic features for **5** include relatively shielded ^13^C (86 ppm) and ^31^P (211 ppm) nuclei, as compared to the corresponding environments in **4**. In a similar fashion, **5** displays two separate ^1^H resonances at 3.92 and 5.00 ppm, which are more shielded than the analogous signals in **4**, but chemically inequivalent, in line with restricted rotation about a P=C π-bond. Akin to **4**, a NOESY correlation determined the relative orientation of the hydrogens, with the most shielded hydrogen being closer to the ^*t*^Bu groups of the pincer ligand. Finally, a computational study revealed orbital interactions in the [P­(=CH_2_)­(NAd)]^−^ ligand in **5** to be reminiscent of those in an allyl group (Figure 6C). Thus, the HOMO–3 depicts a π-conjugated in-phase combination of *p*-orbitals on the N, P, and C atoms, whereas the HOMO depicts a nonbonding combination with a nodal plane on P, and opposite phases of the 2*p* orbitals on N and C. Furthermore, the Mayer (1.589) and Wiberg (1.918) bond orders for the P=C bond are diminished in comparison to their counterparts in **4**, but clearly in line with multiple-bond character, whereas the ellipticity (ε = 0.5710) indicates directionality of electron density in line with π-bonding. Likewise, the P=N bond possesses bond orders (Mayer: 1.643, Wiberg: 1.928) and ellipticity (0.2829) in line with a π-linkage.

**Figure 6:**
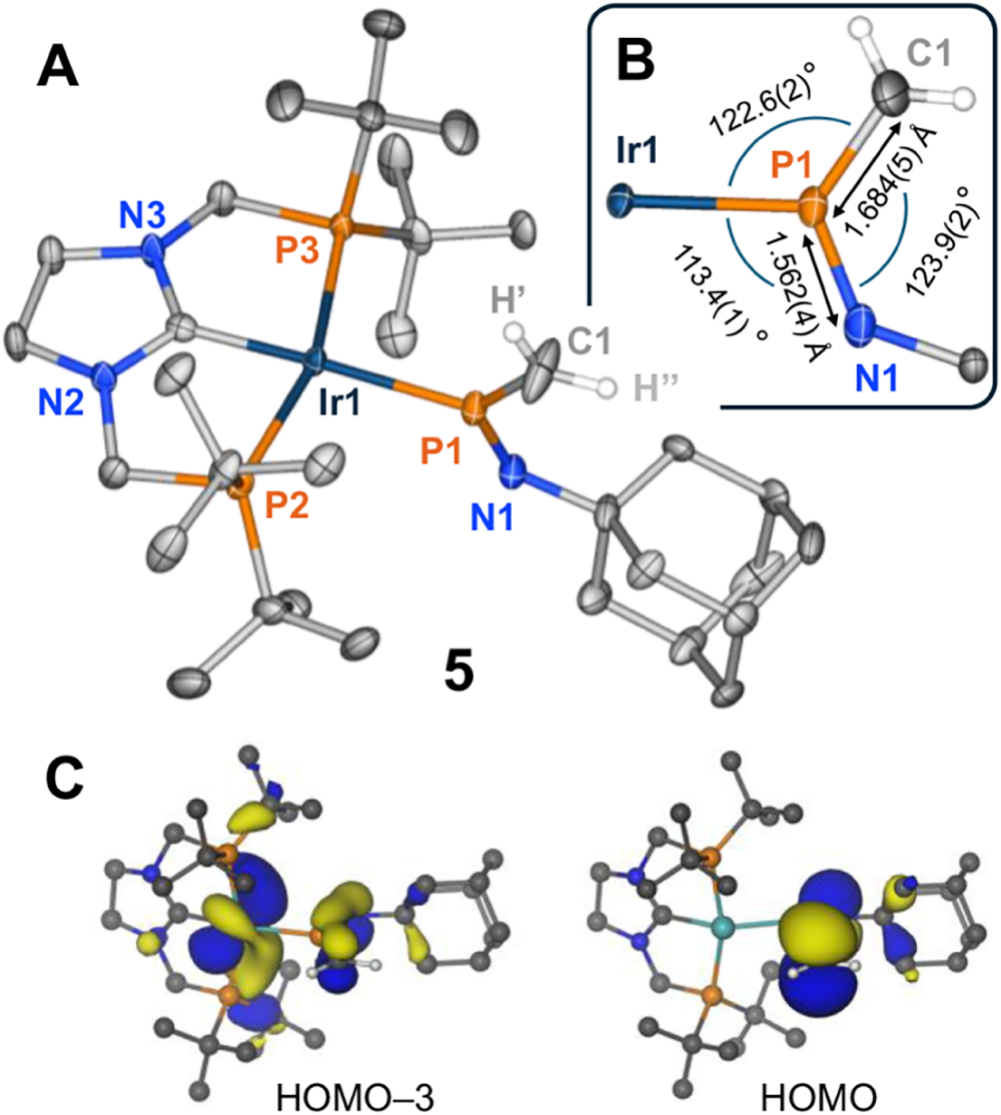
A: X-ray crystallographic structure of **5** recorded at 100(2) K. Thermal ellipsoids are at 50% probability. Rotational disorder in the Ad group and cocrystallized toluene are not shown. H atoms are omitted, except for H′ and H″. B: Metrics for {P­(=CH_2_)­(NAd)}^−^ motif. C: Frontier molecular orbitals of **5** showing allene-type bonding within the [P­(=CH_2_)­(NAd)]^−^ ligand (HOMO, HOMO–3) calculated using DFT (TPSSh-D3/def2-TZVP) and portrayed at an isovalue of ±0.04 au.

## Conclusion

In summary, we have developed two unique metallaphosphinidene coupling strategies to form a substituent-free phosphavinyl ligand. Photolysis of a pincer-scaffolded iridium phosphaethynolate complex, **2**, generates a putative triplet metallaphosphinidene, [(PCP)­Ir­(P)­(CO)], which dimerizes to form a bimetallic complex with a side-on bound {P_2_} ligand, **3**, in the absence of other reactants. When photolysis is instead conducted in the presence of a phosphorus ylide, a net coupling of the putative metallaphosphinidene and a methylene group forms an unprecedented phosphavinyl motif, **4**, [(PCP)­Ir­(P=CH_2_)]. We further uncover a thermal metallaphosphinidene transfer route to **4**; this mechanistically distinct pathway produces a conjugated phosphorus ylide, PhMe_2_PCHCOCH_3_, bearing a [C_3_] chain derived from one CO and two CH_2_ fragments. Structural characterization, isotopic labeling, variable-temperature NMR, multinuclear 2D NMR, and IR spectroscopic studies combined with theoretical calculations on complex **4** unravel a stereochemically rigid and rotationally locked [P=CH_2_]^−^ π-bonded moiety. The phosphavinyl ligand in **4** constitutes a deprotonated ligand form of the elusive phosphaethylene molecule, HP=CH_2_, and we scrutinize the impact of the iridium fragment on the switching dynamics of the P=C double bond. From an array of scenarios ranging from direct rotation about the [P=CH_2_]^−^ double-bond, side-on coordination of [P=CH_2_]^−^ to iridium, to linear coordination of [P=CH_2_]^−^, a linear transition geometry, {Ir=P=CH_2_} is responsible for a drastically lowered isomerization barrier in **4** compared to classical phosphaalkenes [15.9(4) versus >40 kcal mol^–1^]. Finally, we utilize the phosphavinyl ligand to access other rare main-group motif in its reaction with an azide. Overall, we not only lay out a strategy for transferring an open-shell main-group fragment in two mechanistically distinct ways, but also demonstrate how this phosphinidene coupling methodology provides access to unique main-group ligand architectures that are virtually impossible to generate through classic synthetic means. Guided by the present subvalent atom-transfer strategy, other exotic π-bonded pnictogen constructs are underway.

## Supplementary Material



## Notes

Synthetic procedures; NMR, IR, and UV–vis spectroscopic data, kinetic data, crystallographic tables, and computational studies (PDF)

## Abbreviations

Ad: 1-adamantyl
Ar: aryl
Ar*: 2,6-di­{3,5-di­(2,4,6-triisopropylphenyl)­phenyl}­phenyl
cod: 1,5-cyclooctadiene
Et: ethyl
H_2_PCP: 1,3-bis­(di-*tert*-butylphosphinomethyl)­imidazolidine
Me: methyl
Mes*: 2,4,6-^*t*^Bu_3_-C_6_H_2_
Ph: phenyl
^*t*^Bu: *tert*-butyl.
