# Supplementary material for: Metallaphosphinidene Coupling with a Phosphorus Ylide to Form a Phosphavinyl [P=CH2]− Ligand
Source: J Am Chem Soc. 2026 Jun 8;148(24):25096–106. doi: 10.1021/jacs.6c06304 (PMC13307361; doi:10.1021/jacs.6c06304)
Supplement: Supplementary file 1 [file ja6c06304_si_001.pdf]

Supplementary Information

For

Metallaphosphinidene Coupling with a Phosphorus Ylide to Form a Phosphavinyl $[P{=}CH_2]^-$ Ligand

Mattias Tan,[1] Christian Sandoval-Pauker,[2] Zoltan Takacs,[1] Anders Reinholdt[1*]

1 Department of Chemistry, Lund University, Naturvetarvägen 22, 22100 Lund, Sweden
anders.reinholdt@chem.lu.se

2 Department of Chemical and Biomolecular Engineering, Rice University, 6100 Main Street, Houston, TX 770052, USA

1 Contents

1 Contents **S2**

2 Materials and Methods **S4**

3 Syntheses **S6**

3.1 Synthesis of [(PCP)IrCl] (**1**) S6

3.2 Synthesis of [(PCP)Ir(PCO)] (**2**) S6

3.3 Generation of [(PCP)Ir(CO)$_2$(η^2,η^2;μ^2-P_2)] (**3**) S7

3.4 Synthesis of [(PCP)Ir(P=CH_2)] (**4**) S7

*Method A. Photolysis of [(PCP)Ir(PCO)] (**2**) and PhMe$_2$P=CH$_2$* S7

*Method B. Heating of [(PCP)Ir(PCO)] (**2**) and PhMe$_2$P=CH$_2$* S7

*Method C. Photolysis of [(PCP)Ir(PCO)] (**2**) and Ph$_3$P=CH$_2$* S8

3.5 Identification of [(PCP)Ir(CO)] S8

3.6 Identification of PhMe$_2$PCHCOCH$_3$ S9

3.7 Synthesis of [(PCP)Ir{P(=CH_2)(NAd)}] (**5**) S9

3.8 Attempted Reactions between PhMe$_2$PCH$_2$ and CO to Form PhMe$_2$PCHCOCH$_3$ S10

4 NMR Spectroscopy **S11**

4.1 NMR Spectral Data for [(PCP)IrCl] (**1**) S11

4.2 NMR Spectral Data for [(PCP)Ir(PCO)] (**2**) S14

4.3 NMR Spectral Data for [{(PCP)(OC)Ir}$_2$(η^2,η^2;μ_2-P_2)] (**3**) S17

4.4 NMR Spectral Data for [(PCP)Ir(P=CH_2)] (**4**) S18

4.4.1 NMR Spectral Data for 33% ^{13}C-enriched [(PCP)Ir(P=$^{13}CH_2$)] (**4-^{13}C**) S25

4.4.2 NMR Spectral Data for [(PCP)Ir(CO)](PCO) S27

4.4.3 NMR Spectral Data for Reaction Mixtures Forming [(PCP)Ir(P=CH_2)] (**4**); Methods A (Photolysis, PhMe$_2$PCH$_2$), B (Heating, PhMe$_2$PCH$_2$), and C (Photolysis, Ph$_3$PCH$_2$) S30

4.5 NMR Spectral Data for PhMe$_2$P=CHCOMe S32

4.5.1 NMR Spectral Data for 33% ^{13}C-enriched Ph($^{13}CH_3$)$_2$P=^{13}CHCO^{13}CH$_3$ S34

4.6 NMR Spectral Data for [(PCP)Ir{P(=CH_2)(NAd)}] (**5**) S36

4.6.1 NMR Spectral Data for 33% ^{13}C-enriched [(PCP)Ir{P(=$^{13}CH_2$)(NAd)}] (**5-^{13}C**) S40

5 IR Spectroscopy **S42**

5.1 IR Spectral Data for [(PCP)IrCl] (**1**) S42

5.2 IR Spectral Data for [(PCP)Ir(PCO)] (**2**) S43

5.3 IR Spectral Data comparison between [(PCP)IrCl] (**1**) and [(PCP)Ir(PCO)] (**2**) S44

5.4 IR Spectral Data for [{(PCP)(OC)Ir}$_2$(η^2,η^2;μ_2-P_2)] (**3**) S45

5.5 IR Spectral Data for [(PCP)Ir(P=CH_2)] (**4**). S46
5.5.1 IR Spectral Data for [(PCP)Ir(P=CH_2)] (**4**) and 33% ^{13}C-enriched (**4-^{13}C**). S47
5.6 IR Spectral Data for [(PCP)Ir(CO)](PCO) S48
5.7 IR Spectral of [(PCP)Ir{P(=CH_2)(NAd)}] (**5**). S49
6 UV-vis Spectroscopy S50
6.1 UV-vis Spectral Data for [(PCP)IrCl] (**1**) S51
6.2 UV-vis Spectral Data for [(PCP)Ir(PCO)] (**2**) S52
6.3 UV-vis Spectral Data for [(PCP)Ir(PC=H_2)] (**4**) S53
6.4 UV-vis Spectral Data for [(PCP)Ir{P(=CH_2)(NAd)}] (**5**) S54
7 Crystallographic Data S54
7.1 Crystallographic Tables S54
7.2 Thermal Ellipsoid Plot of [(PCP)IrCl] (**1**) S56
7.3 Thermal Ellipsoid Plot of [(PCP)Ir(PCO)] (**2**) S57
7.4 Thermal Ellipsoid Plot of [(PCP)Ir$(CO)_2$(η^2,η^2;μ^2-P_2)] (**3**) S58
7.5 Thermal Ellipsoid Plot of [(PCP)Ir(P=CH_2)] (**4**) S59
7.6 Thermal Ellipsoid Plot of [(PCP)Ir(CO)](PCO) S60
7.7 Thermal Ellipsoid Plot of PhMe_2P=CHCOMe S61
7.8 Thermal Ellipsoid Plot of [(PCP)Ir{P(=CH_2)(NAd)}] (**5**) S62
8 Kinetic Studies by NMR Spectroscopy S63
9 Computational Studies S64
9.1. Computational methodology S64
9.2 Computational data S65
9.3 Scenario A – Rotation about P=C bond S81
9.4 Scenario B – π-bonded intermediate S82
9.5 Scenario C – Cumulenic Intermediate S83
9.6 Scenario D – Rotation about Ir–P σ-bond S84
9.7 Cartesian Coordinates S85
10 References S116

2 Materials and Methods

All synthetic operations were performed in Vigor glove boxes under a purified nitrogen atmosphere. A UV flashlight (Darkbeam Model V5, V5-DB-395nm, 1.3 W) and UV lamp 450 nm, 30 W were used for photolysis experiments. Hexane (Fisher Scientific), and toluene (Fisher Scientific), were purified with a SPS system (MBraun SPS 800). Tetrahydrofuran (Sigma Aldrich), and diethyl ether (Sigma Aldrich) were stored over sodium with benzophenone as indicator, distilled by trap-to-trap transfer *in vacuo*, and degassed by freeze-pump-thaw cycles and stored over 4 Å molecular sieves to further reduce traces of residual water. Benzene-d_6 and toluene-d_8 (Sigma Aldrich) were stored over a potassium mirror overnight, sublimed/distilled by trap-to-trap transfer *in vacuo*, and degassed by freeze-pump-thaw cycles. Celite and 4 Å molecular sieves were activated *in vacuo* overnight at 175 °C. The H_2(PCP) pincer ligand[1], Na(OCP) · 2.5 dioxane,[2] methylenetriphenylphosphorane (Ph_3PCH_2)[3] and methylenedimethylphenylphosphorane ($PhMe_2PCH_2$)[4, 5] were prepared according to published procedures. [Ir(μ_2-Cl)(cod)]$_2$, triphenylphosphine, dimethylphenyl phosphine, methyl iodide, methyl iodide-^{13}C (99% ^{13}C), potassium bis(trimethylsilyl)amide (95%) (Sigma Aldrich) were used as received. Methyltriphenylphosphonium iodide [$PPhMe_3$]I and trimethylphenylphosphonium iodide [$PPhMe_3$]I were dried at 80 °C *in vacuo* for 18 h before use.

Crystallographic studies were carried out on single crystals, which were coated with NVH oil, mounted at the end of a MiTeGen Dual-Thickness Micromount, and placed in the nitrogen cold stream of the diffractometer. Data were collected and processed using Oxford Diffraction Xcalibur Eos diffractometer (Mo K_α radiation), operated *via* CrysAlisPro software.[6] The crystal structures were solved using SHELXT (intrinsic phasing) and refined using SHELXL-2018 (least squares),[7, 8] with data processing carried out in Olex2.[9] Non-hydrogen atoms were refined anisotropically. Hydrogen atoms were placed at calculated positions and refined as riding atoms with isotropic displacement parameters (U_{iso} = 1.2 U_{eq} of the parent atom for CH_2 groups, and U_{iso} = 1.5 U_{eq} of the parent atom for CH_3 groups). For atoms demonstrating high disorder ISOR restraints were applied.

Crystals of **3** were relatively weakly diffracting [$I/\sigma(I)$ = 8.5], and as a result, the recorded dataset has a relatively high value for R_{int} (14.7%, C-alert in checkCIF), leading to some light atoms (C and N) having somewhat deform thermal ellipsoids.

Elemental analyses were carried out by Mikroanalytisches Laboratorium Kolbe (Oberhausen, Germany).

IR spectroscopic studies were carried out using a Bruker ALPHA II spectrometer by measuring solid samples pressed into pellets using dry KBr as matrix.

NMR spectroscopic studies were carried out using Bruker 400 MHz and 500 MHz, spectrometers equipped with J. Young NMR tubes. ^{1}H and ^{13}C NMR chemical shifts are referenced to residual solvent signals (C_6D_6: ^{1}H: 7.16 ppm, ^{13}C: 128.06 ppm, toluene-d_8: ^{1}H: 2.09 ppm, ^{13}C: 128.33 ppm). 85% H_3PO_4 in H_2O defines 0 ppm for ^{31}P; these frequencies are based on indirect referencing to ^{1}H.

UV-vis spectroscopic studies were carried out using an Agilent Technologies Cary 60 Spectrometer equipped with 1.00 cm quartz cuvettes sealed with Teflon stoppers and electrical tape.

3 Syntheses

3.1 Synthesis of [(PCP)IrCl] (1)

Under a N_2 atmosphere, [Ir(μ_2-Cl)(cod)]$_2$ (1.00 g, 1.49 mmol) was added together with H_2(PCP) (1.12 g, 2.88 mmol) dissolved with 15 ml toluene in a 100 ml Straus flask, freeze-pumped and then filled with H_2 before being heated at 110 °C and stirred for 16 h, resulting in a colour change from red to yellow. The solution was then filtered through celite and recrystallized from toluene at –35 °C. [(PCP)IrCl] (**1**) was obtained as yellow crystals in 1.53 g, 2.49 mmol, 83.7% yield based on [Ir(μ_2-Cl)(cod)]$_2$. Crystals suitable for X-ray crystallography were obtained from a toluene solution of [(PCP)IrCl] subjected to hexane diffusion at –35 °C.

^{1}H-NMR, (400 MHz, C_6D_6) δ 2.74 (vt, *J* = 2.4 Hz, 4H, N-C**H**$_2$-P), 2.50 (s, 4H, N-(C**H**$_2$)$_2$-N), 1.45 (vt, *J* = 6.5 Hz, 36H, C(C**H**$_3$)$_3$) ppm. **^{13}C NMR**, (101 MHz, C_6D_6) δ 198.59 (t, *J* = 5.3 Hz, N-**C**-N, 49.17 (t, *J* = 4.2 Hz, N-(**C**H$_2$)$_2$-N), 44.99 (t, *J* = 12.8 Hz, N-**C**H$_2$-P), 35.67 (t, *J* = 8.8 Hz, **C**(CH$_3$)$_3$), 29.49 (t, *J* = 3.1 Hz, C(**C**H$_3$)$_3$) ppm. **^{31}P-NMR**, (162 MHz, C_6D_6) δ 77.80 (s, **P**-Ir-**P**) ppm. **UV/Vis**, THF, *λ* [nm, *ε* (max/sh, M^{-1} cm^{-1})]: 299 (max, 5900), 366 (max, 1500), 391 (sh, 1100), 420 (max, 800), 468 (max, 350). **Elemental analysis**, calculated for $C_{21}H_{44}N_2P_2IrCl$: C: 41.07%, H: 7.22%, N: 4.56%; found: C: 40.86%, H: 7.24%, N: 4.53%.

3.2 Synthesis of [(PCP)Ir(PCO)] (2)

Under a N_2 atmosphere, [(PCP)IrCl] (**1**) (50.0 mg, 81.4 µmol) was added together with Na(PCO) · 2.5 dioxane (24.6 mg, 0.081 mmol) in a 4 ml vial, dissolved with 2 ml THF and stirred for 16 h in the dark resulting in a colour change from yellow to orange. The solution was concentrated *in vacuo,* redissolved in benzene and filtered through celite. [(PCP)Ir(PCO)] (**2**) was obtained (51.0 mg, 80.0 µmol), 98.2% yield based on [(PCP)IrCl]. Crystals suitable for X-ray crystallography were obtained from a THF solution of [(PCP)Ir(PCO)], concentrated at 25 °C using toluene as a sorbent.

^{1}H NMR, (400 MHz, C_6D_6) δ 2.75 (vt, *J* = 2.3 Hz, 4H, N-C**H**$_2$-P), 2.47 (s, 4H, N-(C**H**$_2$)$_2$-N), 1.39 (vt, *J* = 13.11 Hz, 36H, C(C**H**$_3$)$_3$) ppm. **^{13}C NMR**, (151 MHz, C_6D_6) δ 203.88 (dt, *J* = 42.5, 5.6 Hz, N-**C**-N), 178.37 (dt, *J* = 77.2, 5.8 Hz, P=**C**=O), 49.72 (t, *J* = 4.2 Hz, N-(**C**H$_2$)$_2$-N), 46.25 (td, *J* = 12.8, 3.5 Hz, N-**C**H$_2$-P), 36.12 (t, *J* = 9.4, **C**(CH$_3$)$_3$), 29.87 (q, *J* = 3.5 Hz, C(**C**H$_3$)$_3$), ppm. **^{31}P NMR**, (162 MHz, C_6D_6) δ 78.81 (s, **P**-Ir-**P**) , -355.06 (s, Ir-**P**=C=O) ppm. **IR**, solid in KBr matrix, *v* (cm^{-1}): 1841 (antisymmetrical PCO mode), 1092 (symmetrical PCO mode). **UV/Vis**, THF, *λ* [nm, *ε* (max/sh, M^{-1} cm^{-1})]: 314 (max, 14.000), 390 (max, 6000), 395

(max, 6500), 505 (max, 1600). **Elemental analysis**, calculated for $C_{22}H_{44}N_2IrOP_3$: C: 41.43%, H: 6.95%, N: 4.39%; found: C: 41.18%, H: 7.00%, N: 4.37%.

3.3 Generation of [{(PCP)(OC)Ir}$_2$(η^2,η^2;μ_2-P_2)] (3)

[(PCP)Ir(PCO)] (**2**) (100 mg, 157 µmol) was dissolved in 7 ml benzene and added to a 50 ml Straus flask with a stirrer and irradiated with a 450 nm LED lamp (30 W) under stirring for 25 min (solution turned dark green, then dark red). The solution was concentrated *in vacuo* before being redissolved in pentane and filtered through celite. The solution was then left to crystallize (yellow crystals) by concentrating with toluene as a sorbent at -30 °C. [{(PCP)(OC)Ir}$_2$(η^2,η^2;μ_2-P_2)] (**3**) was isolated from the multicomponent mixture as yellow crystals in 2% yield (2.1 mg, 1.6 µmol).

^{31}P NMR, (162 MHz, C_6D_6) δ 43.91 – 43.40 (m, Ir–**P**^{t}Bu$_2$), 17.39 (s, **P**^{t}Bu$_2$), -109.72 (dt, *J* = 443.0, 16.0 Hz, **P=P**), -149.69 (dt, *J* = 443.0, 11.3 Hz, **P=P**). **IR**, solid in KBr matrix, *v* (cm^{-1}): 1960 (CO), 1880 (CO).

3.4 Synthesis of [(PCP)Ir(P=CH$_2$)] (4)

*Method A. Photolysis of [(PCP)Ir(PCO)] (**2**) and PhMe$_2$P=CH$_2$*

[(PCP)Ir(PCO)] (**2**) (9.7 mg, 15.5 µmol) was added together with PhMe$_2$P=CH$_2$ (7.0 mg, 45 µmol, 3.0 eq) in a J-Young NMR tube and dissolved in 1 ml benzene. The reaction mixture was exposed to 395 nm light (1.3 W) for 25 minutes, and the solution turned dark green for about five minutes, and then gradually turned dark red (after ten minutes). The solution was then filtered through celite, and the volatiles were removed *in vacuo*. The residue was dissolved in 0.5 ml C_6D_6 together with [(PCP)IrCl] (**1**, 10.1 mg, 16.4 µmol) used as an internal standard for integration of the NMR spectrum. Based on integration of the ^{31}P NMR spectrum, [(PCP)Ir(P=CH$_2$)] (**4**) formed in 60% spectroscopic yield, [(PCP)Ir(CO)](PCO) formed in 15% spectroscopic yield, and PhMe$_2$P=CHCOMe formed in 10% spectroscopic yield (Figure S35).

*Method B. Heating of [(PCP)Ir(PCO)] (**2**) and PhMe$_2$P=CH$_2$*

[(PCP)Ir(PCO)] (**2**) (40.0 mg, 62.7 µmol) was added together with PhMe$_2$P=CH$_2$ (28.8 mg, 189 µmol, 3.0 eq) in a J-Young NMR tube and dissolved in 1 ml benzene and heated at 50 °C for 16 h. The colour of the solution turned from orange to dark red. The solution was then filtered through celite and concentrated *in vacuo* and the remaining solid was washed with pentane (removing PPhMe$_2$) and recrystallized from diethyl ether. At this point, the solution contains

both [(PCP)Ir(P=CH_2)] (**4**) and a new ylide, $PhMe_2P$=CHCOMe, which have similar but not identical solubilities. To separate these complexes, slow concentration of an ether solution of the reaction mixture (at –35 °C, using toluene as sorbent) results in dark red crystals of [(PCP)Ir(P=CH_2)] depositing on the inside of the crystallization vial, whereas the ylide, $PhMe_2P$=CHCOMe, remains in the mother liquor. If necessary, this recrystallization method must be repeated to completely separate the ylide, $PhMe_2P$=CHCOMe, from [(PCP)Ir(P=CH_2)]. Ultimately, [(PCP)Ir(P=CH_2)] was obtained as dark red crystals (18 mg, 29 µmol), 46% yield based on [(PCP)Ir(PCO)]. Crystals suitable for X-ray crystallography were obtained from a THF solution of [(PCP)Ir(P=CHc_2)], concentrated at 25 °C using toluene as a sorbent.

*Method C. Photolysis of [(PCP)Ir(PCO)] (**2**) and $Ph_3P=CH_2$*

[(PCP)Ir(PCO)] (**2**) (40.0 mg, 62.7 µmol) was added together with $Ph_3P=CH_2$ (52.0 mg, 188 µmol, 3.0 eq) in a J-Young NMR tube and exposed to 395 nm light (1.3 W) for 20 min. The reaction mixture showed severeal products along with (**4**) and [(PCP)Ir(CO)](PCO) as seen in Figure S37.

^{1}H NMR (500 MHz, C_6D_6): δ 10.28 (d, *J* = 16.4 Hz, 1H, P=C**H**$_2$), 9.07 (d, *J* = 27.6 Hz, 1H, P=C**H**$_2$), 2.88 (vt, *J* = 2.6 Hz, 4H, N-C**H**$_2$-P), 2.56 (s, 4H, N-(C**H**$_2$)$_2$-N), 1.39 (vt, *J* = 6.6 Hz, 36H, C(C**H**$_3$)$_3$) ppm. **^{13}C NMR** (126 MHz, C_6D_6) δ 215.43 (N-**C**-N), 158.15 (d, *J* = 59.5 Hz, P=**C**H$_2$), 50.35 (s, N-(**C**H$_2$)$_2$-N), 47.43 (t, *J* = 12.7 Hz, N-**C**H$_2$-P), 36.53 (t, *J* = 9.8 Hz, **C**(CH$_3$)$_3$), 29.94 (s, C(**C**H$_3$)$_3$). ppm. **^{31}P NMR** (203 MHz, C_6D_6) δ 536.00 (t, *J* = 15.56 Hz, **P**=CH$_2$), 88.09 (d, *J* = 15.1 Hz, **P**-Ir-**P**). **IR**, solid in KBr matrix, *v* (cm^{-1}): 942/924 (P=CH_2) for **4/4-^{13}C**. **UV/Vis**, THF, *λ* [nm, *ε* (max/sh, M^{-1} cm^{-1})]: 314 (max, 15000), 389 (max, 9000), 456 (sh, 3900), 517 (max, 1100). **Elemental analysis**, calculated for $C_{22}H_{46}N_2IrP_3$: C: 42.36%, H: 7.43%, N: 4.49%; found: C: 41.88%, H: 7.41%, N: 4.37%.

3.5 Identification of [(PCP)Ir(CO)](PCO)

From 3.4 Method A, the reaction mixture was washed with pentane and diethyl ether before being extracted with benzene and filtered through celite. [(PCP)Ir(CO)](PCO) was crystallized by allowing hexane to diffuse into the solution at 25 °C. Crystals suitable for X-ray crystallography were obtained by recrystallization from a solution of [(PCP)Ir(CO)](PCO) in diethylether, concentrated at –35 °C using toluene as a sorbent.

^{1}H NMR (400 MHz, C_6D_6): δ 3.74 (s, 4H, N-C**H**$_2$-P), 3.65 (s, 4H, N-(C**H**$_2$)$_2$-N), 1.22 (t, *J* = 7.0 Hz, C(C**H**$_3$)$_3$) ppm. **^{13}C NMR** (101 MHz, C_6D_6): δ 51.14 (s, N-(**C**H$_2$)$_2$-N), 37.68 (s, **C**(CH$_3$)$_3$), 31.97(s, N-**C**H$_2$-P), 29.79(s, C(**C**H$_3$)$_3$) ppm. **^{31}P NMR** (162 MHz, C_6D_6): δ 81.20 (s, **P**-Ir-**P**), -391.46 (s, **P**=C=O) ppm. **IR**, solid in KBr matrix, *v* (cm^{-1}): 1966 (CO), 1942 (PCO).

3.6 Identification of PhMe$_2$P=CHCOMe

From 3.4 Method B, PhMe$_2$P=CHCOMe was extracted from the reaction mixture with pentane and recrystallized. The ^{1}H NMR spectral data is in line with the literature.[10] Crystals suitable for X-ray crystallography were obtained from a pentane solution of PhMe$_2$P=CHCOMe, concentrated at –35 °C using toluene as a sorbent.

^{1}H NMR(400 MHz, C_6D_6): δ 7.44-7.39 (m, 2H, C$_6$**H**$_5$), 7.07 – 6.97 (m, 3H, C$_6$**H**$_5$), 3.27 (d, *J* = 29.6 Hz, 1H, OC-**H**C-PPhMe$_2$), 2.34 (d, *J* = 2.0 Hz, 3H, **H**$_3$C-CO), 1.32 (d, *J* = 13.6 Hz, 6H, P-C**H**$_3$). **^{13}C NMR**(101 MHz, C_6D_6): δ 190.61 (d, *J* = 2.7 Hz, **C**O), 131.34 (d, *J* = 2.7 Hz, **C**$_6$H$_5$), 130.76 (d, *J* = 10.0 Hz, **C**$_6$H$_5$), 128.74 (d, *J* = 11.4 Hz, **C**$_6$H$_5$), 128.59 (**C**$_6$H$_5$), 49.68 (OC-H**C**-PPhMe$_2$), 28.69 (d, *J* = 15.0 Hz, P-**C**H$_3$), 11.87 (d, *J* = 61.3 Hz, P-**C**H$_3$). **^{31}P NMR**: (162 MHz, C_6D_6) δ 2.51 (**P**-CH$_3$).

3.7 Synthesis of [(PCP)Ir{P(=CH$_2$)(NAd)}] (5)

[(PCP)Ir(P=CH$_2$)] (**4**) (15.0 mg, 24.0 µmol) was added together with AdN$_3$ (adamantyl azide) (4.3 mg, 24 µmol) in a J-Young tube and dissolved in 1 ml benzene, which resulted in gas formation (N_2) over 10 min. The solution was then filtered through celite, the solvent was removed *in vacuo*. The solid residue was washed with pentane (1 ml), and dissolved in diethyl ether (1 ml), and concentrated at –35 °C using toluene as sorbent. [(PCP)Ir{P(=CH$_2$)(NAd)}] · 0.9 toluene (**5**) was obtained (20.2 mg, 23,6 µmol) in 98.1% yield. Crystals suitable for X-ray crystallography were obtained from a pentane solution of [(PCP)Ir{P(=CH$_2$)(NAd)}] (**5**), concentrated at -30 °C using toluene as a sorbent.

^{1}H NMR (500 MHz, C_6D_6): δ 5.01 (dd, *J* = 11.0, 5.3 Hz, 1H, P=C**H**$_2$), 3.92 (dd, *J* = 19.6, 5.3 Hz, 1H, P=C**H**$_2$), 2.85 (vt, *J* = 2.2 Hz, 4H, N-C**H**$_2$-P), 2.52 (m+s, 6H+4H, overlapping Ad + N-(C**H**$_2$)$_2$-N), 2.32 (s, 3H, Ad), 1.99 (m, 3H, Ad), 1.83 (m, 3H, Ad), 1.48 (vt, *J* = 6.7 Hz, 36H, C(C**H**$_3$)$_3$). **^{13}C NMR** (126 MHz, C_6D_6): δ 215.38 (d, *J* = 99.5 Hz, N-**C**-N), 86.15 (d, *J* = 3.4 Hz, P=**C**H$_2$), 56.02 (d, *J* = 23.2 Hz, Ad), 49.87 (s, N-(**C**H$_2$)$_2$-N), 47.28 (d, *J* = 13.6 Hz, Ad), 46.95

(td, J = 12.3, 6.8 Hz, N-**C**H_2-P), 38.25 (s, Ad), 37.35 (t, J = 9.8 Hz, **C**$(CH_3)_3$), 31.71 (s, Ad), 30.23 (t, C(**C**$H_3)_3$)). **^{31}P NMR** (162 MHz, C_6D_6): δ 211.41 (t, J = 32.5 Hz, **P**=CH_2), 82.94 (d, J = 32.5 Hz, **P**-Ir-**P**). **UV/Vis**, THF, λ [nm, ε (max/sh, M^{-1} cm^{-1})]: 328 (max, 11000), 360 (sh, 3200), 405 (max, 5000), 466 (max, 3500), 550 (max, 900). **Elemental analysis**, calculated for $C_{32}H_{61}IrN_3P_3$ · 0.9 (C_7H_8): C: 53.75%, H: 8.03%, N: 4.91%; found: C: 53.64%, H: 7.99%, N: 4.81%.

3.8 Attempted Reactions between $PhMe_2PCH_2$ and CO to Form $PhMe_2PCHCOCH_3$

Caution: CO is highly toxic. Backfilling procedures were carried out in a well ventilated fumehood. CO leakage detectores were placed immediately outside of the fumehood.

[1] $PhMe_2P=CH_2$ (10 mg, 65.7 µmol) was dissolved in 0.5 ml C_6D_6 in a J-Young NMR tube. The tube tube was frozen with liquid nitrogen, the headspace was evacuated, and then backfilled with gaseous CO (1 bar). The solution was brought to room temperature and left for 1 hour (analyzed by NMR) before being heated at 70 °C over 16 hours (analyzed by NMR). No $PhMe_2PCHCOCH_3$ was detected by ^{1}H and ^{31}P-NMR spectroscopy.

[2] [(PCP)Ir(PCO)] (**2**, 2.1 mg, 3.3 µmol) and $PhMe_2P=CH_2$ (10 mg, 65.7 µmol) were dissolved in 0.5 ml C_6D_6 in a J-Young NMR tube. The tube tube was frozen with liquid nitrogen, the headspace was evacuated, and then backfilled with gaseous CO (1 bar). Complex **2** degraded, whereas no $PhMe_2PCHCOCH_3$ was detected by ^{1}H and ^{31}P-NMR spectroscopy.

[3] [(PCP)Ir(P=CH_2)] (**4**, 2.1 mg, 3.7 µmol) and $PhMe_2P=CH_2$ (10 mg, 65.7 µmol) were dissolved in 0.5 ml C_6D_6 in a J-Young NMR tube. The tube tube was frozen with liquid nitrogen, the headspace was evacuated, and then backfilled with gaseous CO (1 bar). Complex **4** degraded, whereas no $PhMe_2PCHCOCH_3$ was detected by ^{1}H and ^{31}P-NMR spectroscopy.

4 NMR Spectroscopy

4.1 NMR Spectral Data for [(PCP)IrCl] (1)

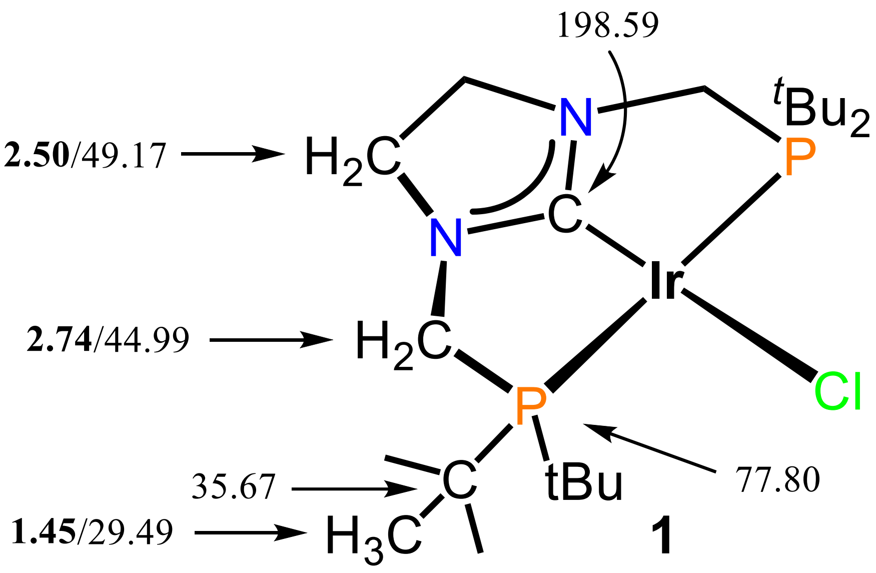

Figure S1. [(PCP)IrCl] (**1**) **^{1}H-**, ^{13}C-, and ^{31}P-NMR shifts.

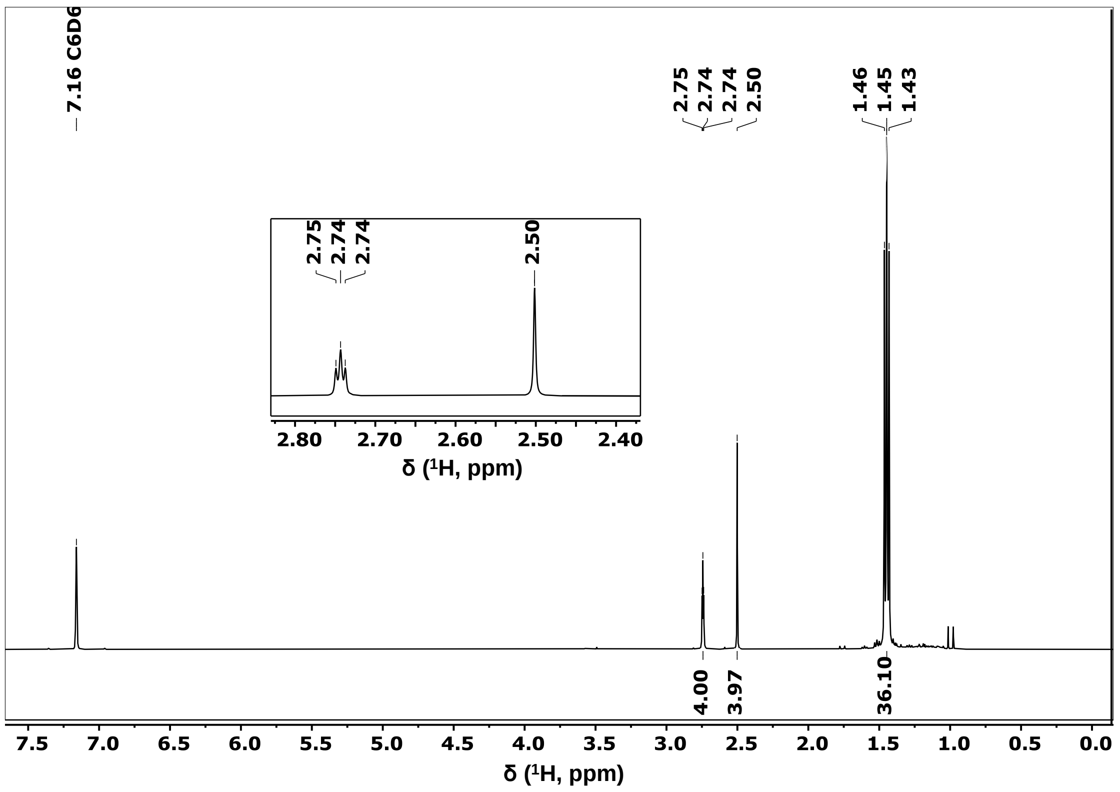

Figure S2. ^{1}H-NMR spectrum of [(PCP)IrCl] (**1**) in C_6D_6 (400 MHz).

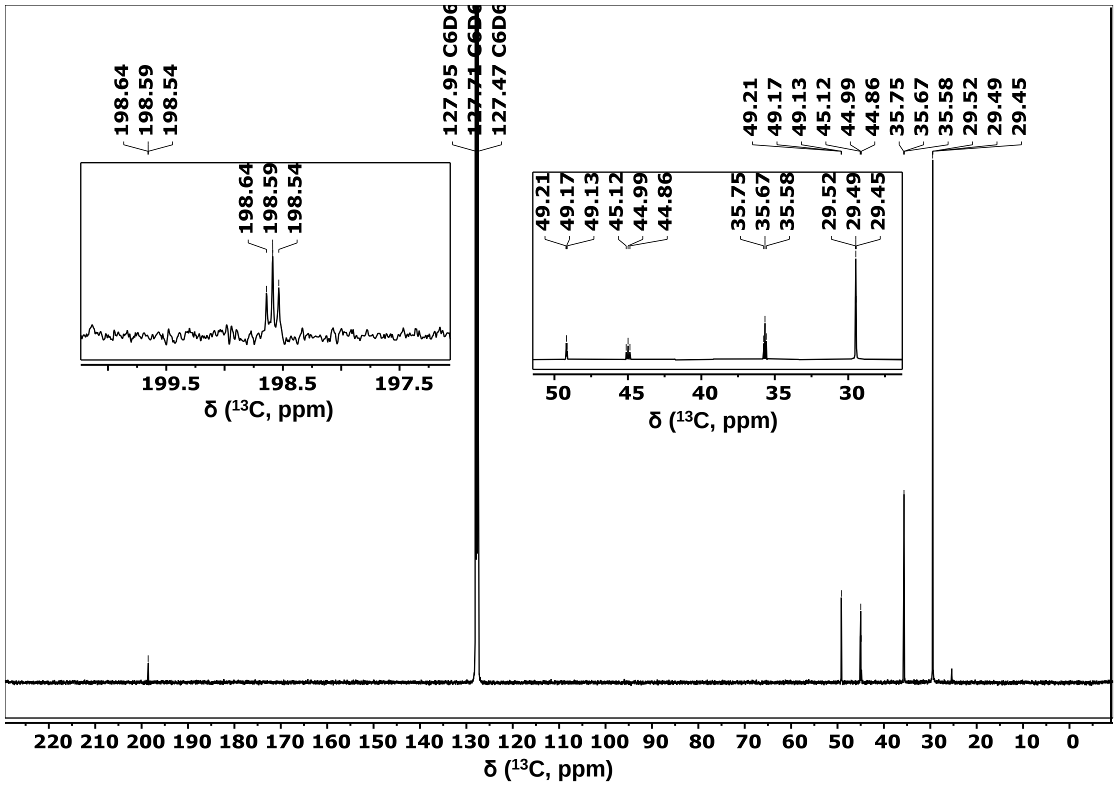

Figure S3. $^{13}C\{^{1}H\}$ NMR spectrum of [(PCP)IrCl] (**1**) in C_6D_6 (101 MHz).

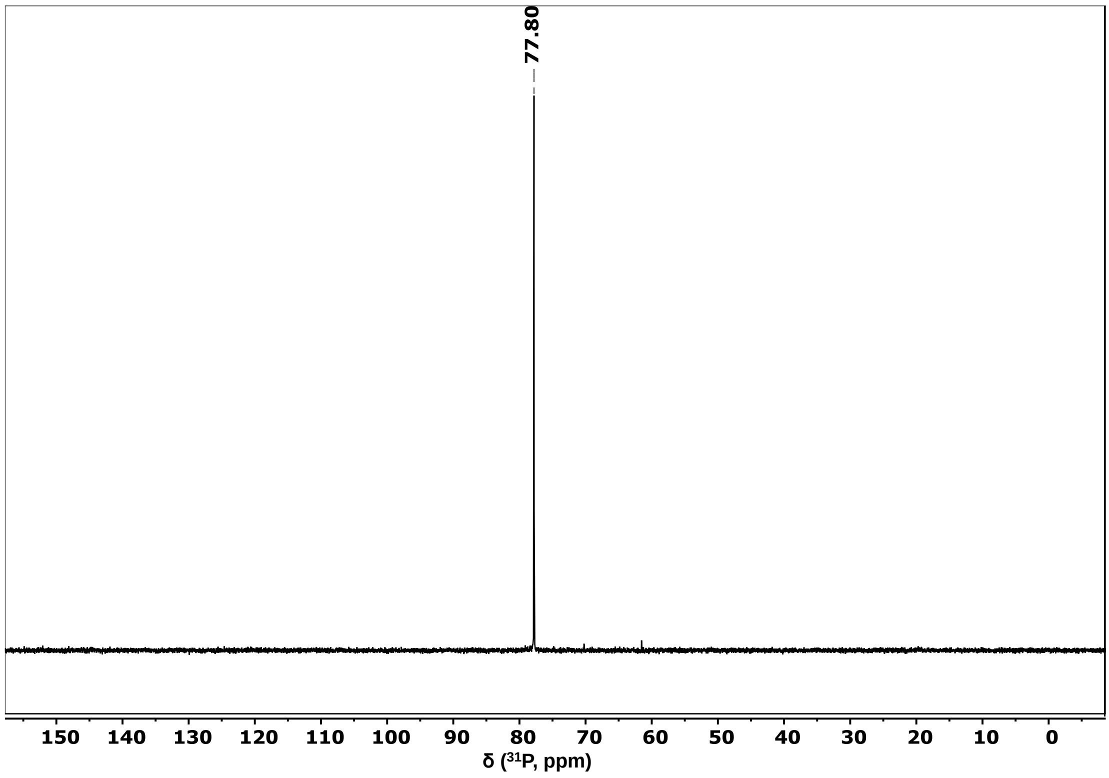

Figure S4. $^{31}P\{^{1}H\}$-NMR spectrum of [(PCP)IrCl] (**1**) in C_6D_6 (162 MHz).

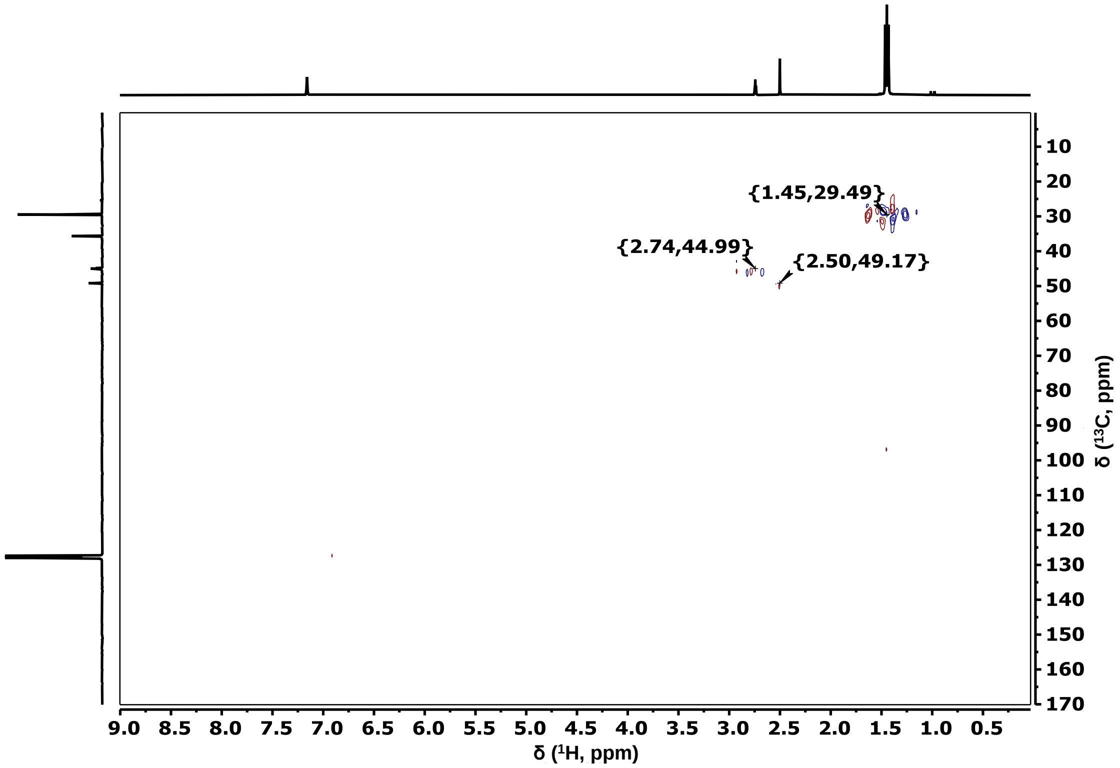

Figure S5. ^1H-^{13}C HSQC NMR spectrum of [(PCP)IrCl] (**1**) in C_6D_6 (400 MHz).

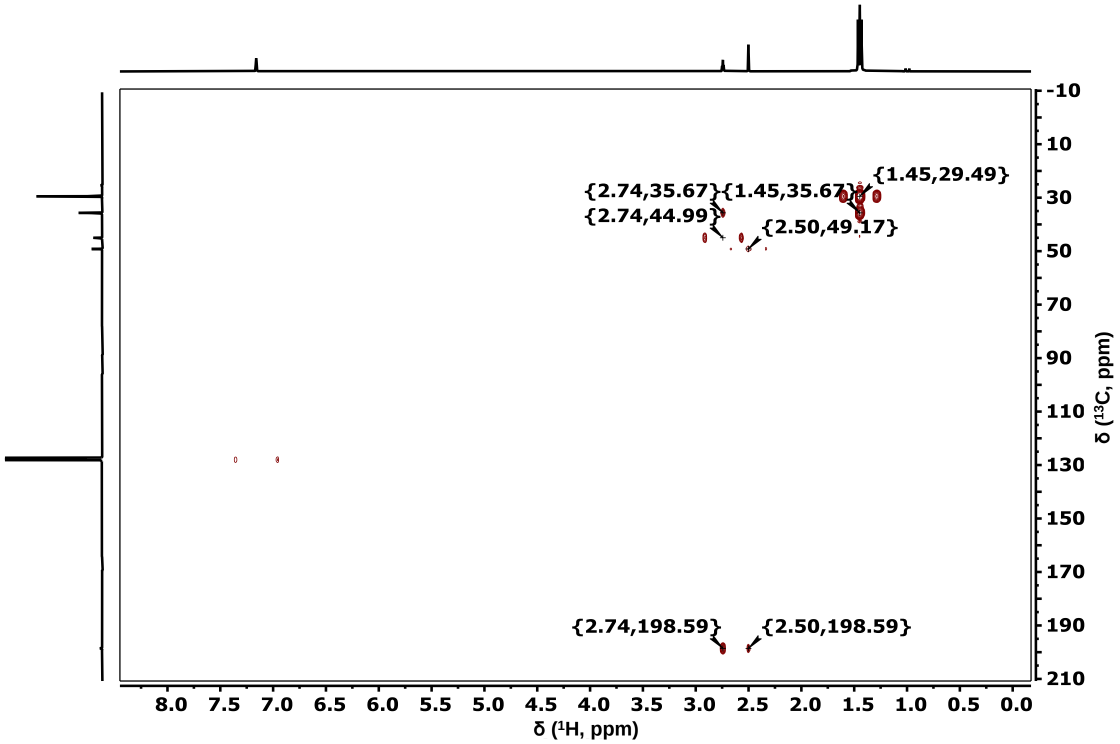

Figure S6. ^1H-^{13}C HMBC NMR spectrum of [(PCP)IrCl] (**1**) in C_6D_6 (400 MHz).

4.2 NMR Spectral Data for [(PCP)Ir(PCO)] (2)

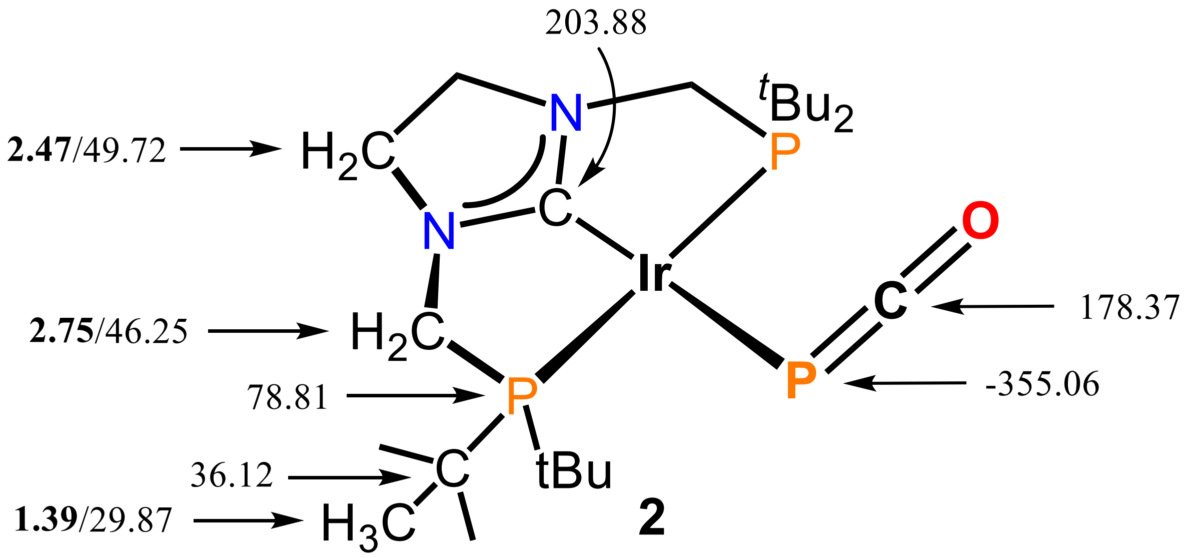

Figure S7. [(PCP)Ir(PCO)] (**2**) **^{1}H-**, ^{13}C-, and ^{31}P-NMR shifts.

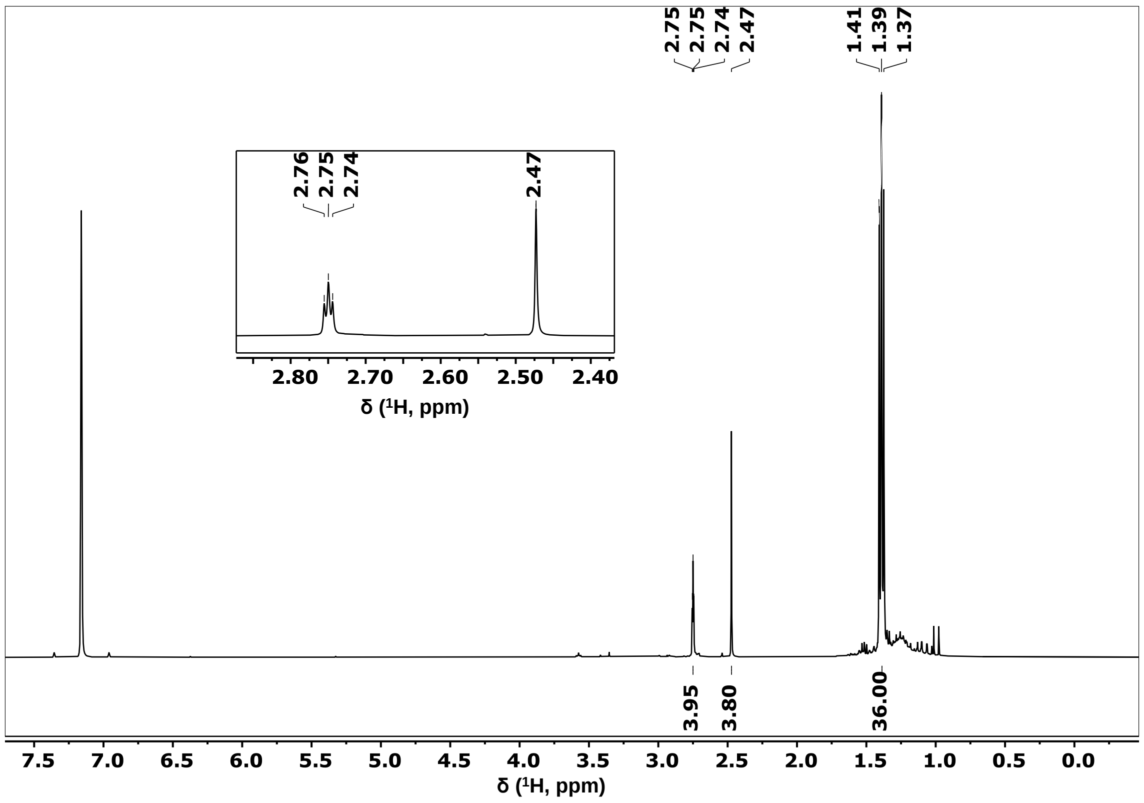

Figure S8. ^{1}H-NMR spectrum of [(PCP)Ir(PCO)] (**2**) in C_6D_6 (400 MHz).

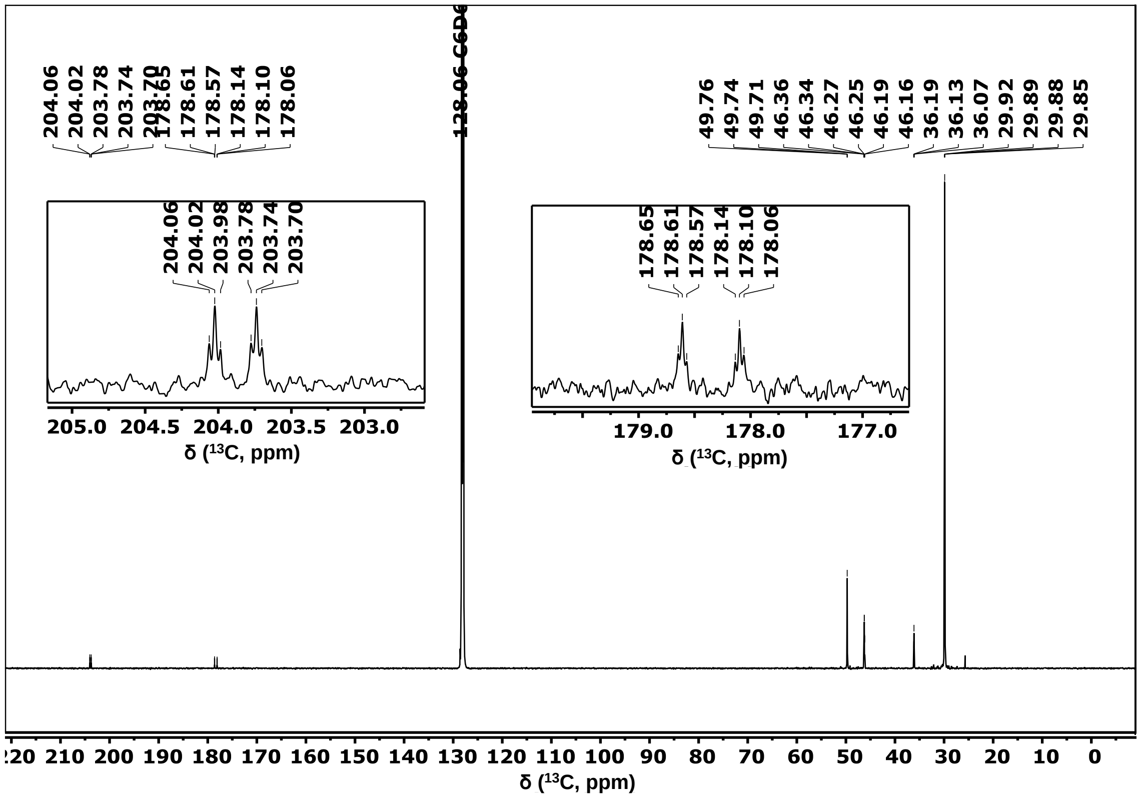

Figure S9. $^{13}C\{^1H\}$ NMR spectrum of [(PCP)Ir(PCO)] (**2**) in C_6D_6 (151 MHz).

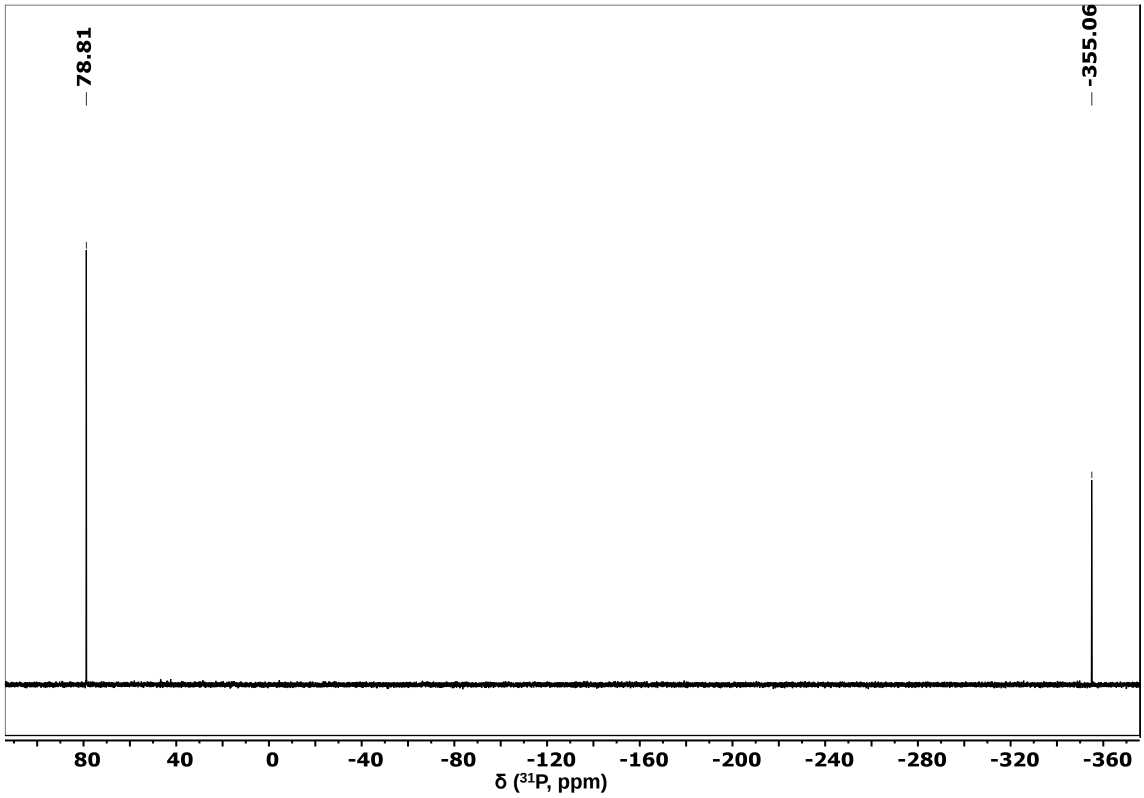

Figure S10. $^{31}P\{^1H\}$-NMR spectrum of [(PCP)Ir(PCO)] (**2**) in C_6D_6 (162 MHz).

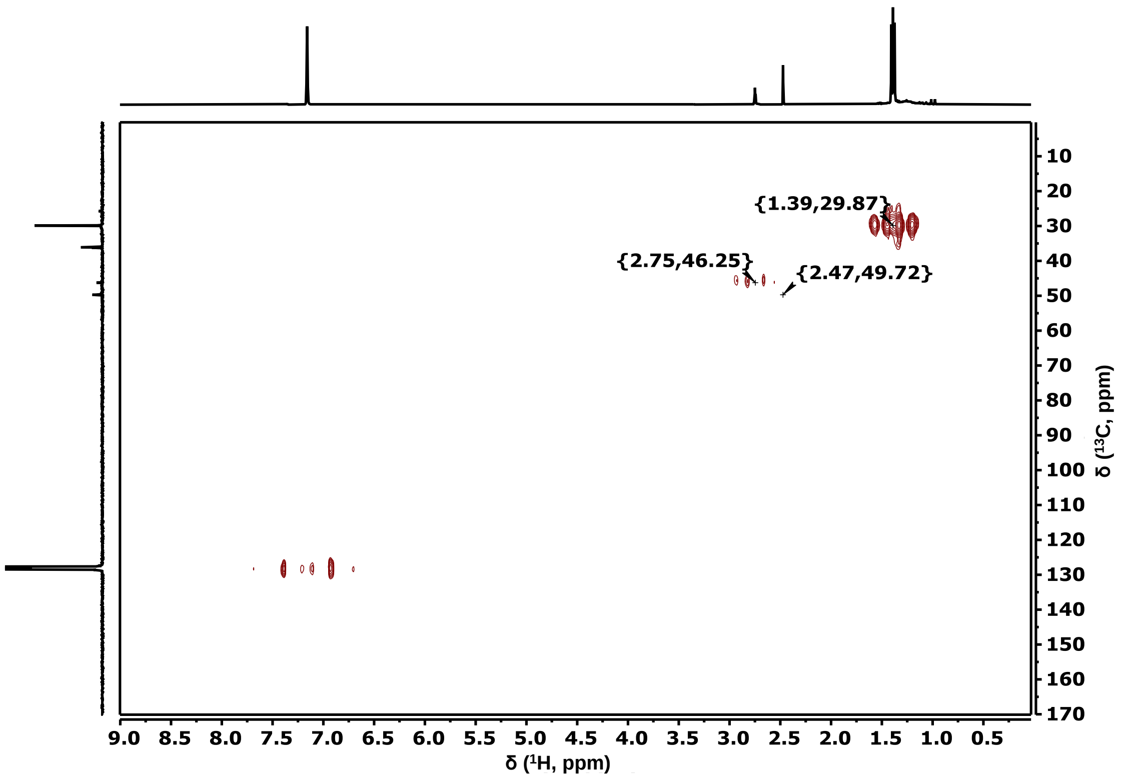

Figure S11. ^{1}H-^{13}C HSQC NMR spectrum of [(PCP)Ir(PCO)] (**2**) in C_6D_6 (400/101 MHz).

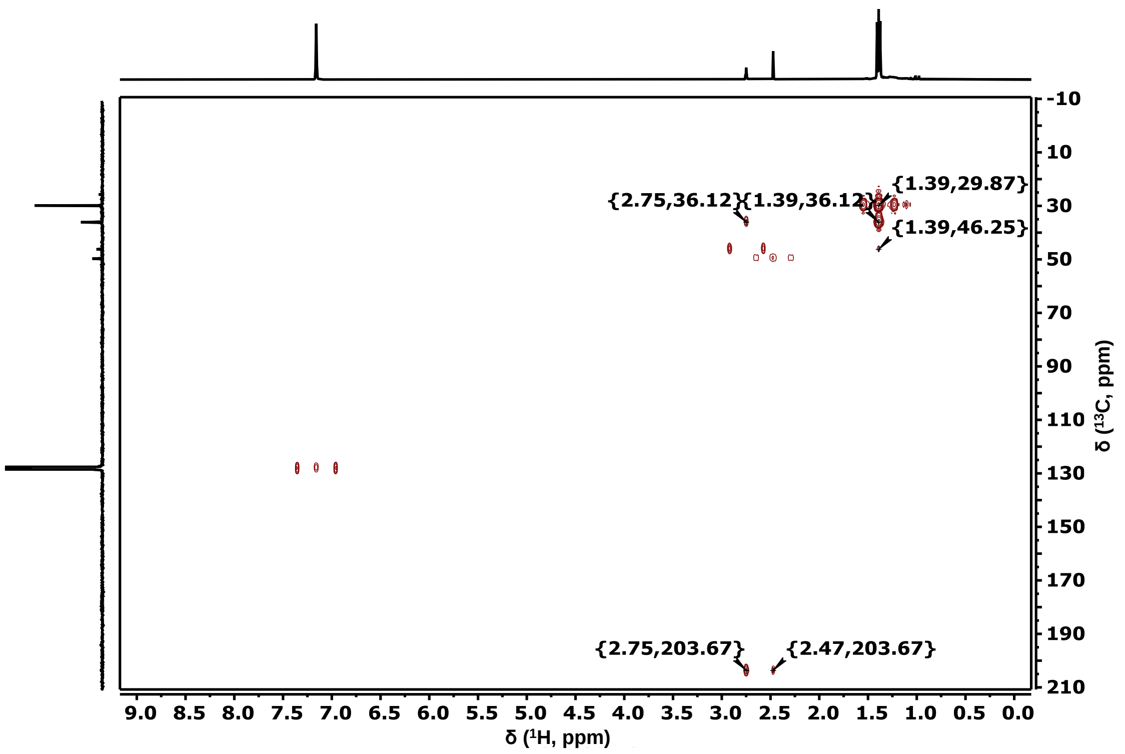

Figure S12. ^{1}H-^{13}C HMBC NMR spectrum of [(PCP)Ir(PCO)] (**2**) in C_6D_6 (400/101 MHz).

4.3 NMR Spectral Data for [{(PCP)(OC)Ir}$_2$(η^2,η^2;μ_2-P$_2$)] (3)

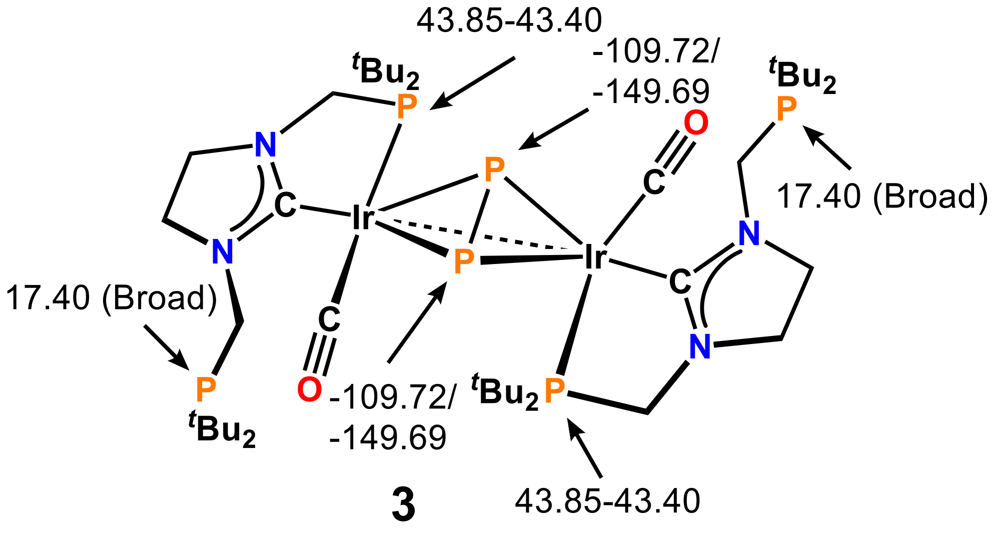

Figure S13. [{(PCP)(OC)Ir}$_2$(η^2,η^2;μ_2-P$_2$)] (**3**) ^{31}P-NMR shift.

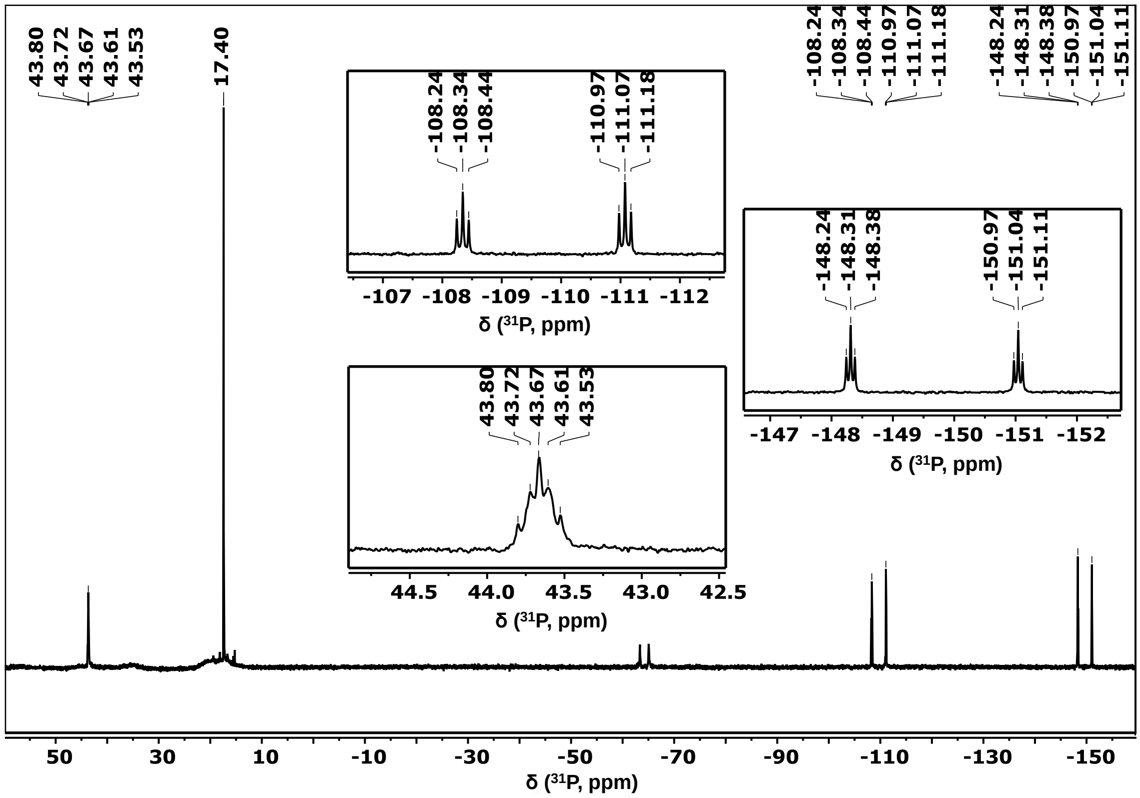

Figure S14. ^{31}P{^{1}H}-NMR spectrum of [{(PCP)(OC)Ir}$_2$(η^2,η^2;μ_2-P$_2$)] (**3**) in C_6D_6 (162 MHz).

4.4 NMR Spectral Data for [(PCP)Ir(P=CH2)] (4)

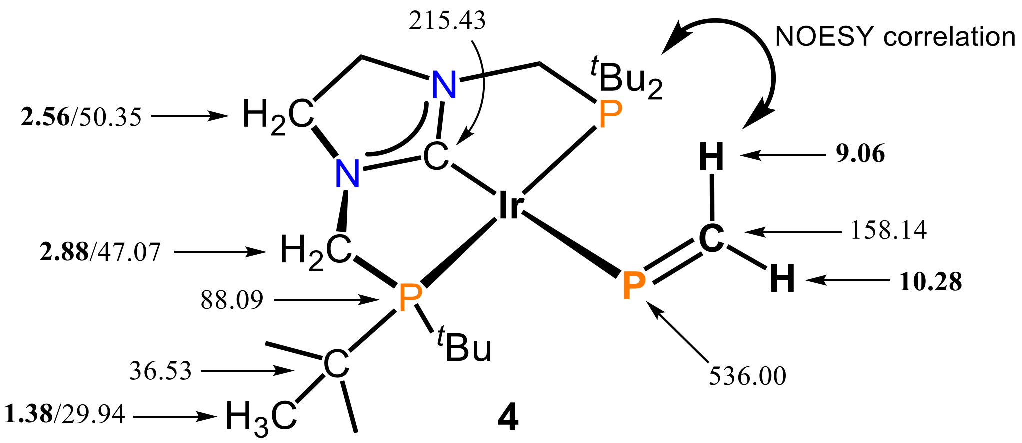

Figure S15 [(PCP)Ir(P=CH2)] (**4**) **^{1}H-**, ^{13}C-, and ^{31}P-NMR shifts.

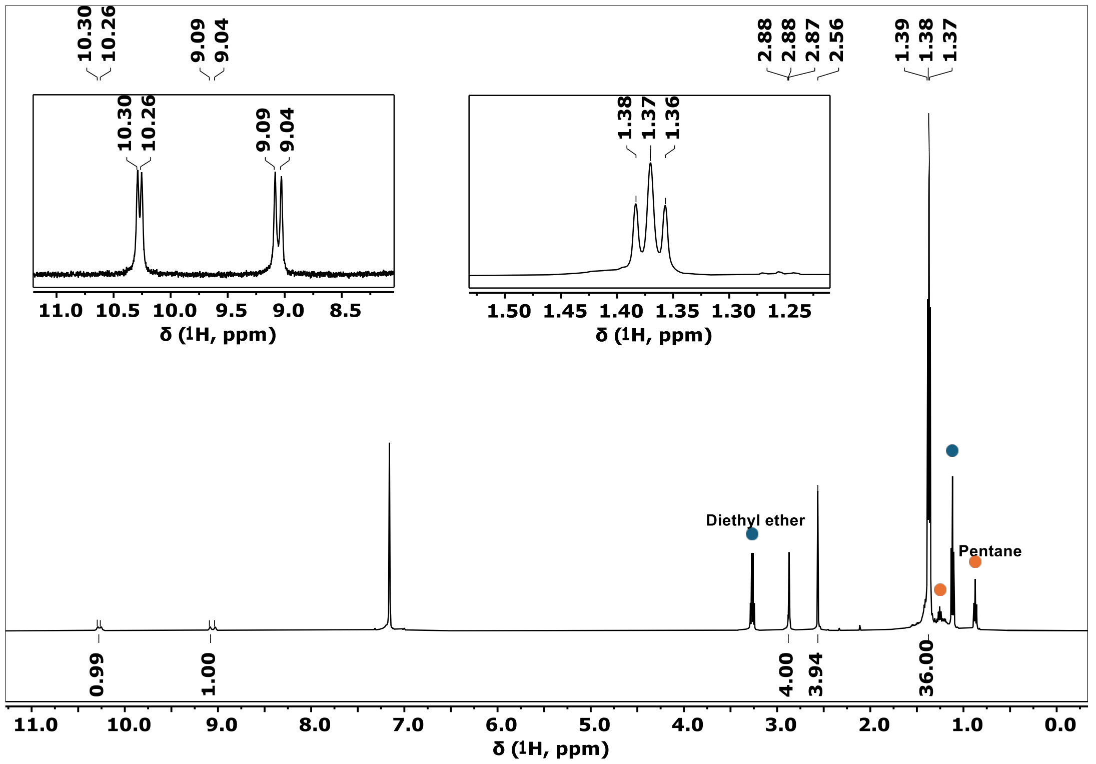

Figure S16. ^{1}H NMR spectrum of [(PCP)Ir(P=CH2)] (**4**) in C_6D_6 (500 MHz). The resonances at 3.26 and 1.12 ppm (diethyl ether) and at 0.87 and 1.22 ppm (pentane) stem from a trace of solvent.

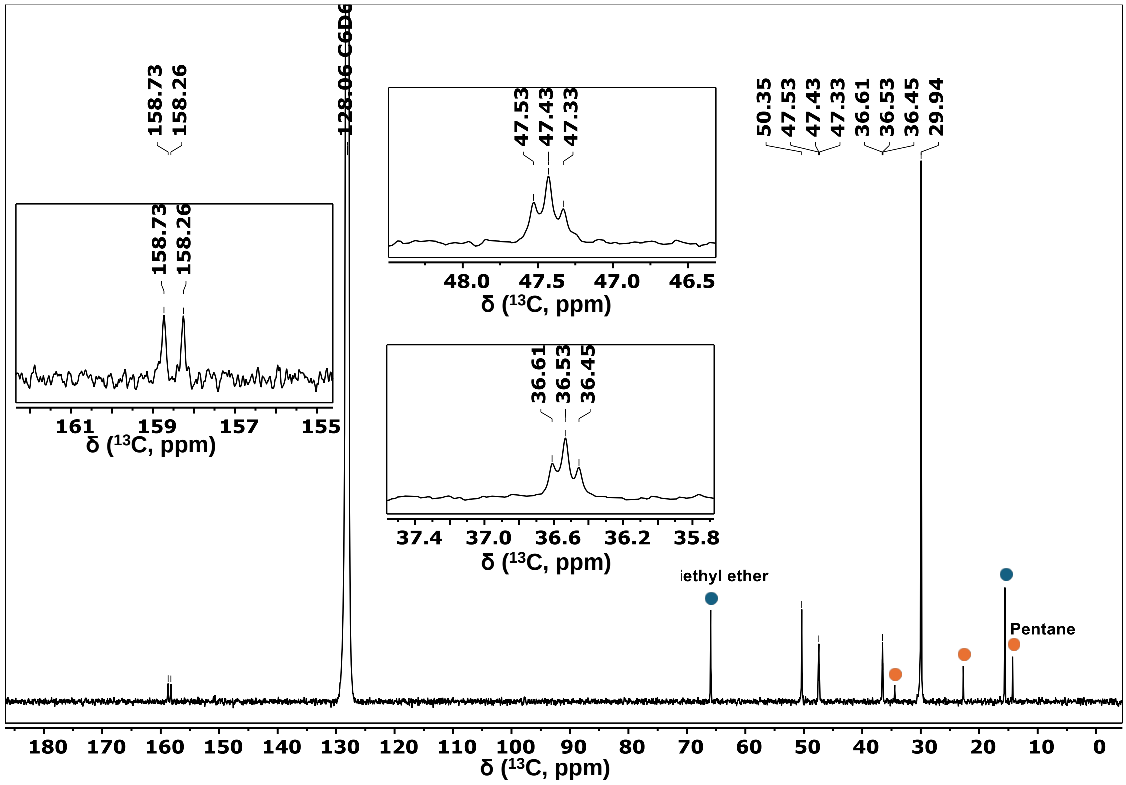

Figure S17. $^{13}C\{^1H\}$ NMR spectrum of [(PCP)Ir(P=CH$_2$)] (**4**) in C_6D_6 (126 MHz). The resonances at 65.92 and 15.60 ppm (diethyl ether) and at 34.44, 22.73 and, 14.28 ppm (pentane) stem from a trace of solvent.

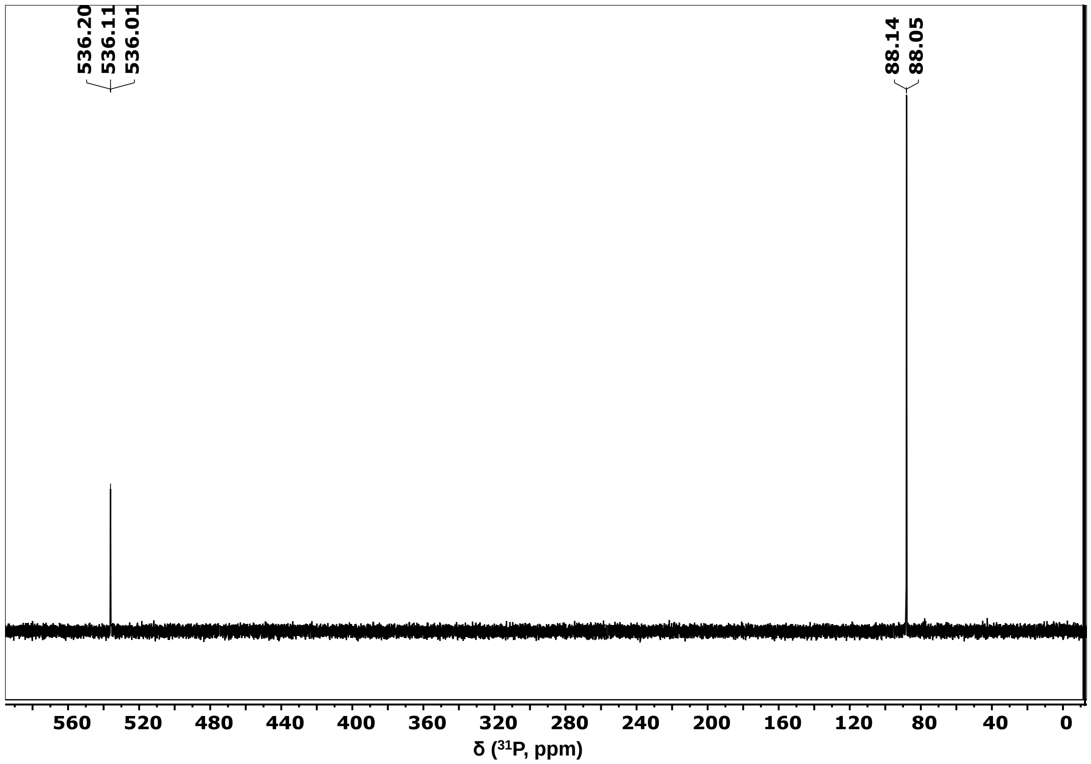

Figure S18. $^{31}P\{^{1}H\}$ NMR spectrum of [(PCP)Ir(P=CH_2)] (**4**) in C_6D_6 (203 MHz).

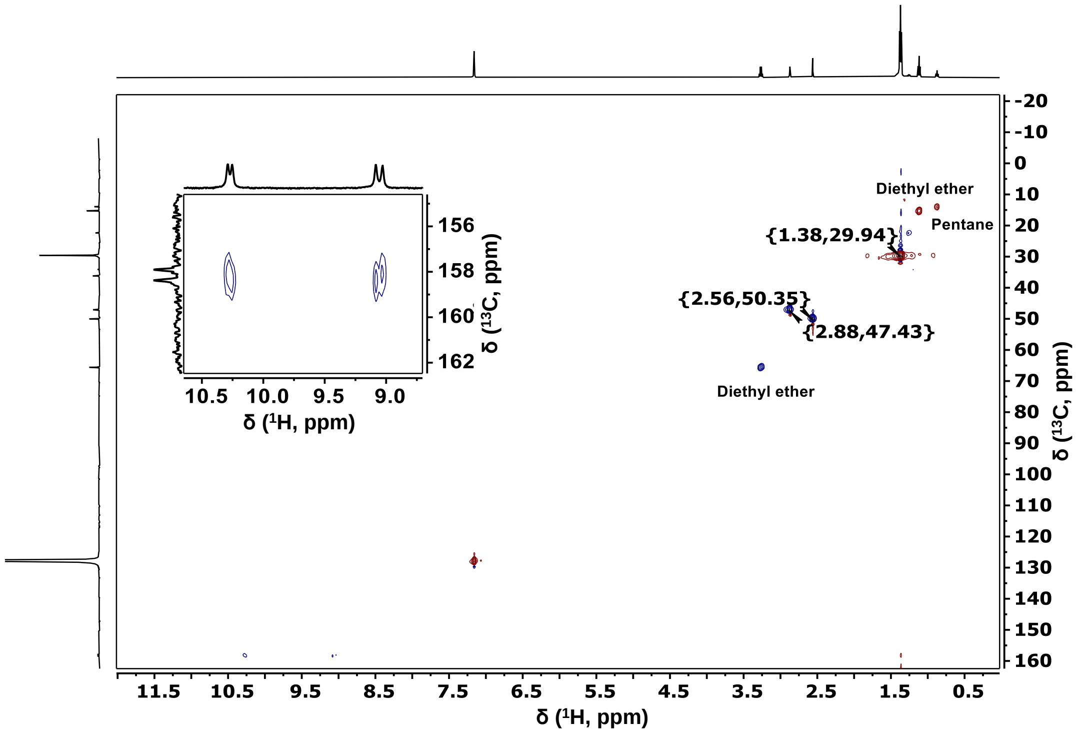

Figure S19. Phase-edited ^{1}H-^{13}C HSQC NMR spectrum of [(PCP)Ir(P=CH_2)] (**4**) in C_6D_6 (500, 126 MHz). The correlation from CH_3 groups (^{t}Bu, red) has negative phase, and correlations from CH_2 groups (P=CH_2 and PCP, blue) have positive phase.

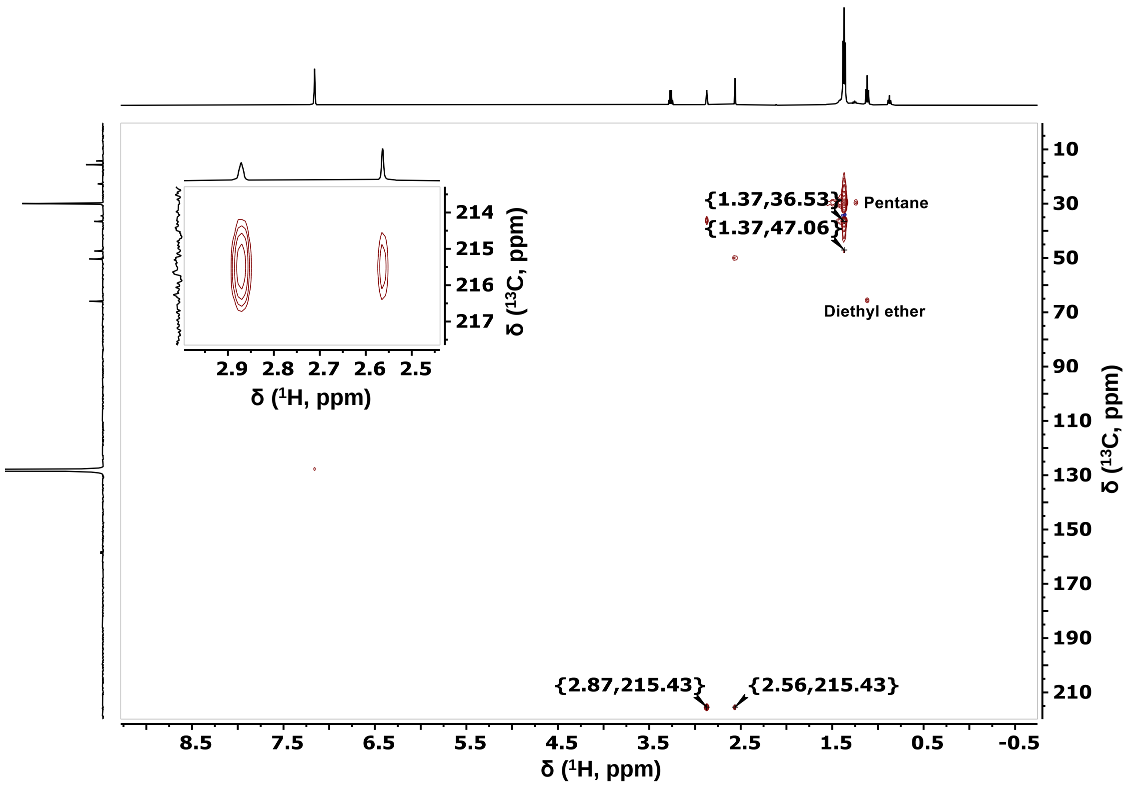

Figure S20. ^{1}H-^{13}C HMBC NMR spectrum of [(PCP)Ir(P=CH_2)] (**4**) in C_6D_6 (500, 126 MHz).

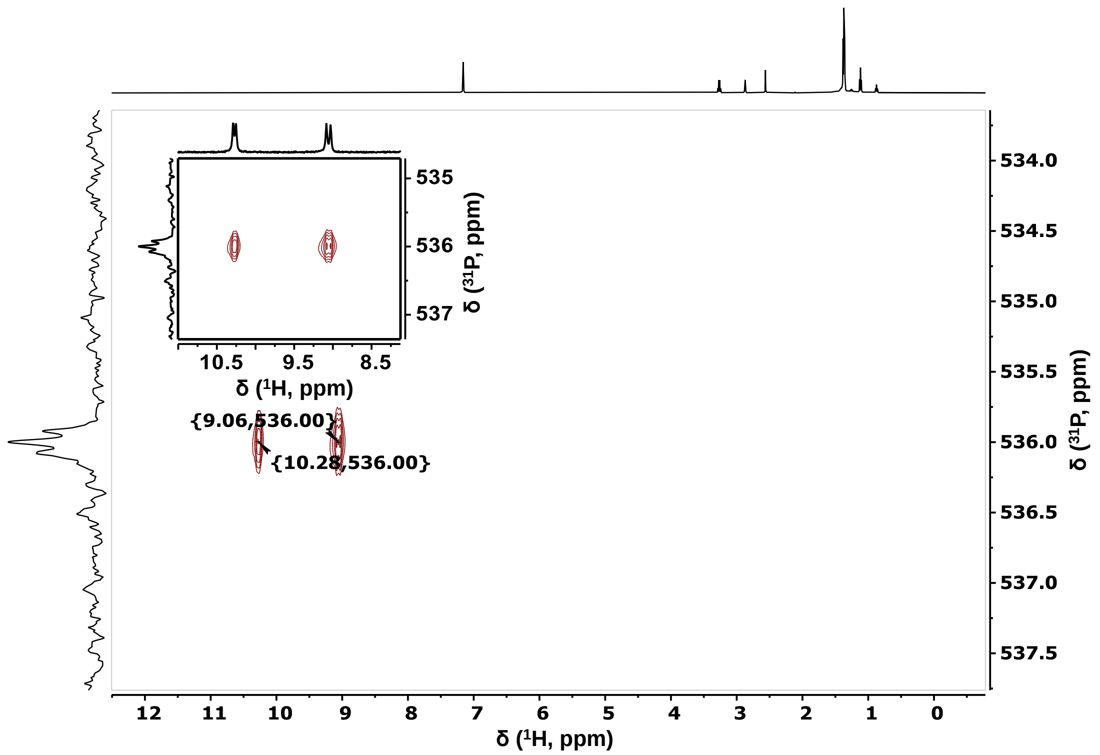

Figure S21. ^{1}H-^{31}P HMBC NMR spectrum of [(PCP)Ir(P=CH_2)] (**4**) in C_6D_6 (500, 203 MHz).

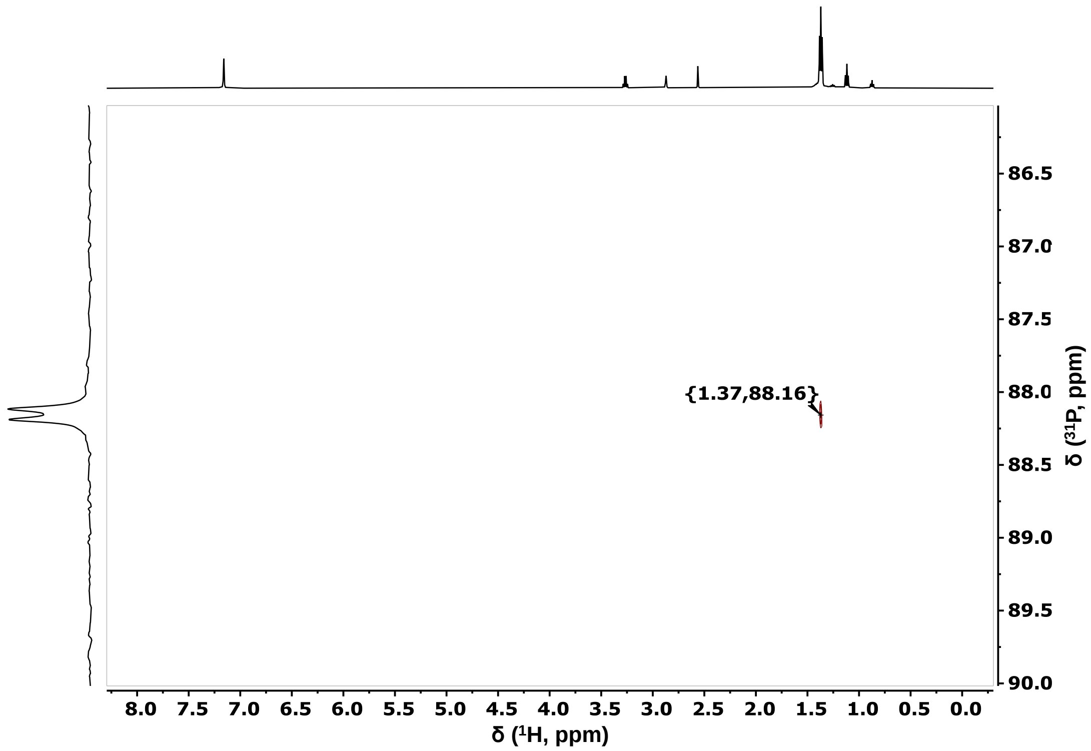

Figure S22. ^{1}H-^{31}P HMBC NMR spectrum of [(PCP)Ir(P=CH_2)] (**4**) in C_6D_6 (500, 203 MHz).

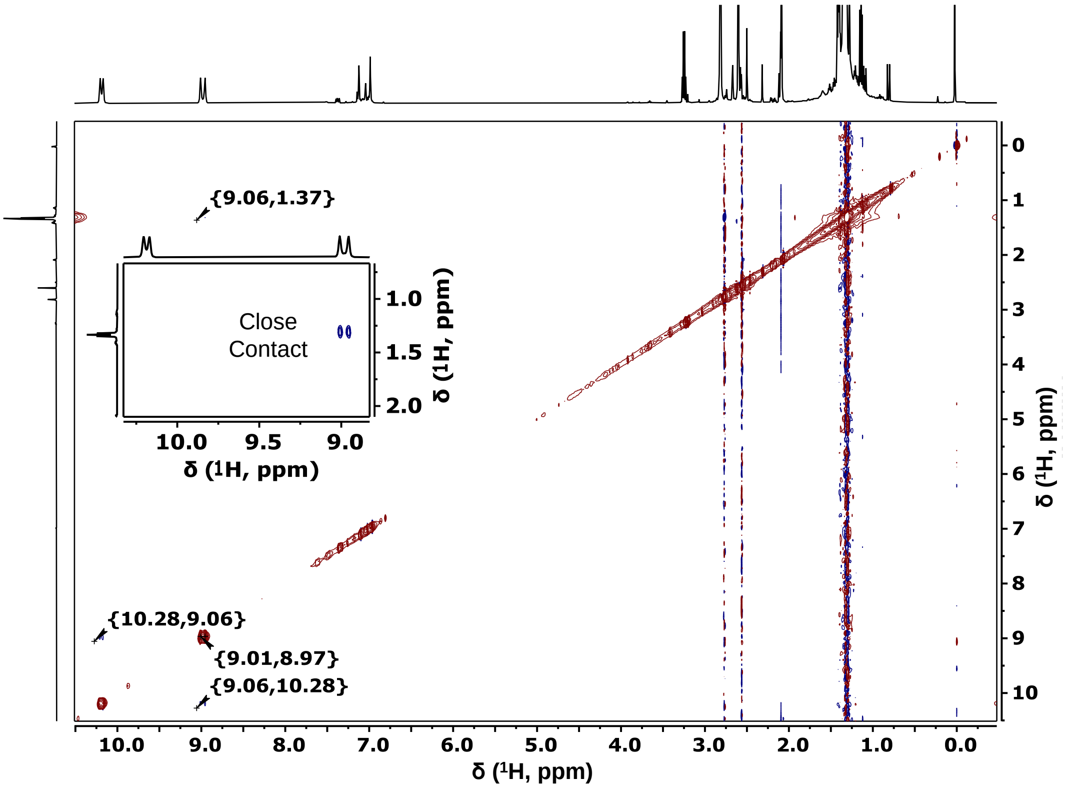

Figure S23. NOESY NMR spectrum of [(PCP)Ir(P=CH_2)] (**4**) in toluene-d_8 (500 MHz), showing a close proximity between the ^{t}Bu groups and H' of the [PCH_2]$^-$ ligand.

The assignment of H' and H" of the [PCH_2]$^-$ ligand is based on a series of NOESY spectra recorded at –25 °C with mixing times ranging from 50 to 300 ms. At this temperature, the chemical exchange is "frozen out" and the cross-peaks are dominated only by the dipole-dipole interaction. All cross peaks have oppositie phase compared to that of the diagonal ones, a tell tale sign of dipole-dipole interaction in the extreme narrowing limit.

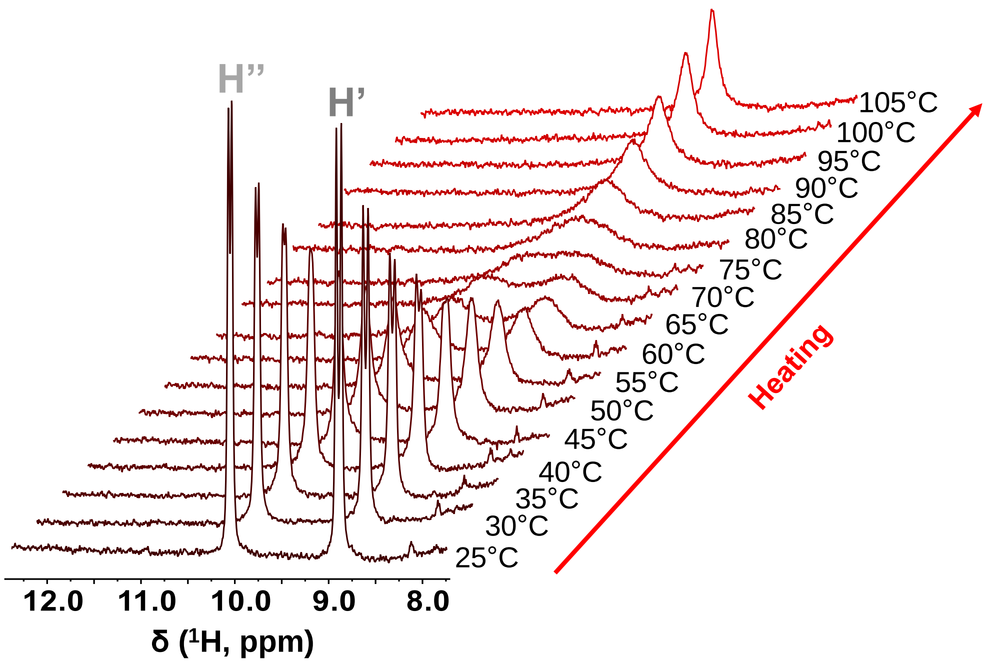

Figure S24. Variable temperature ^{1}H NMR spectra of [(PCP)Ir(P=CH$_2$)] (**4**) showing resonances from the P=CH$_2$ ligand between +25 to +105 °C.

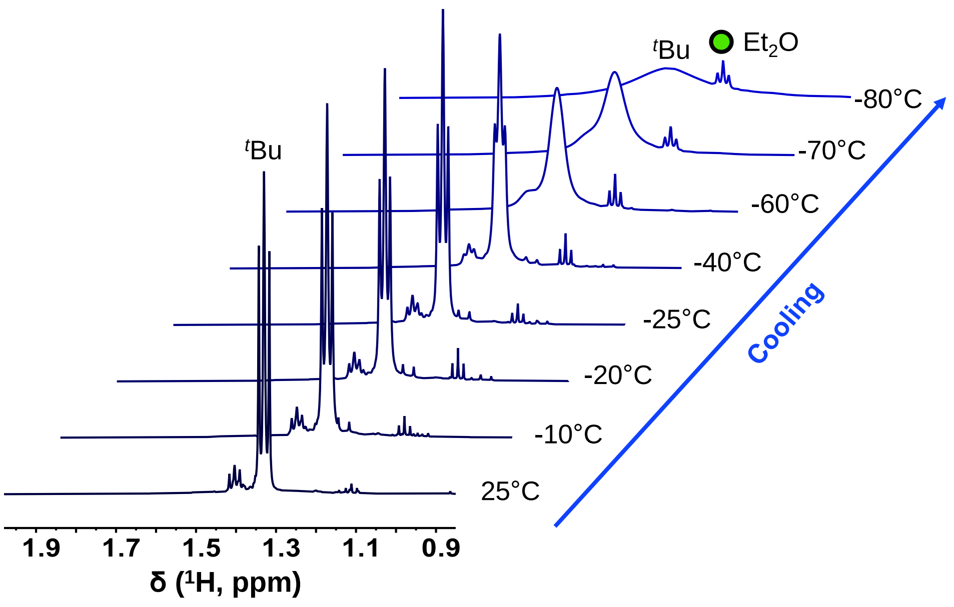

Figure S25. Variable temperature ^{1}H NMR spectra of [(PCP)Ir(P=CH$_2$)] (**4**) showing resonances from the ^{t}Bu ligand between +25 to –80 °C.

4.4.1 NMR Spectral Data for 33% ^{13}C-enriched [(PCP)Ir(P=^{13}CH$_2$)] (4-^{13}C)

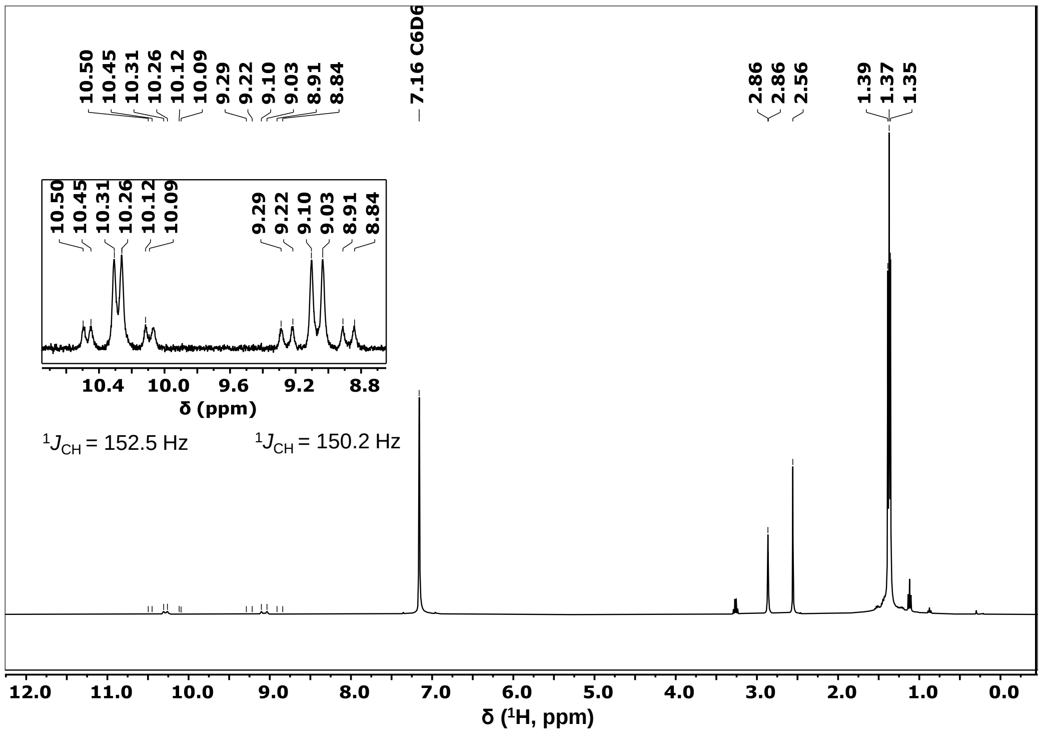

Figure S26. ^{1}H NMR spectrum of 33% ^{13}C-enriched [(PCP)Ir(P=^{13}CH$_2$)] (**4-^{13}C**) in C_6D_6 (400 MHz). The resonances at 3.26 and 1.12 ppm stem from a trace of diethyl ether.

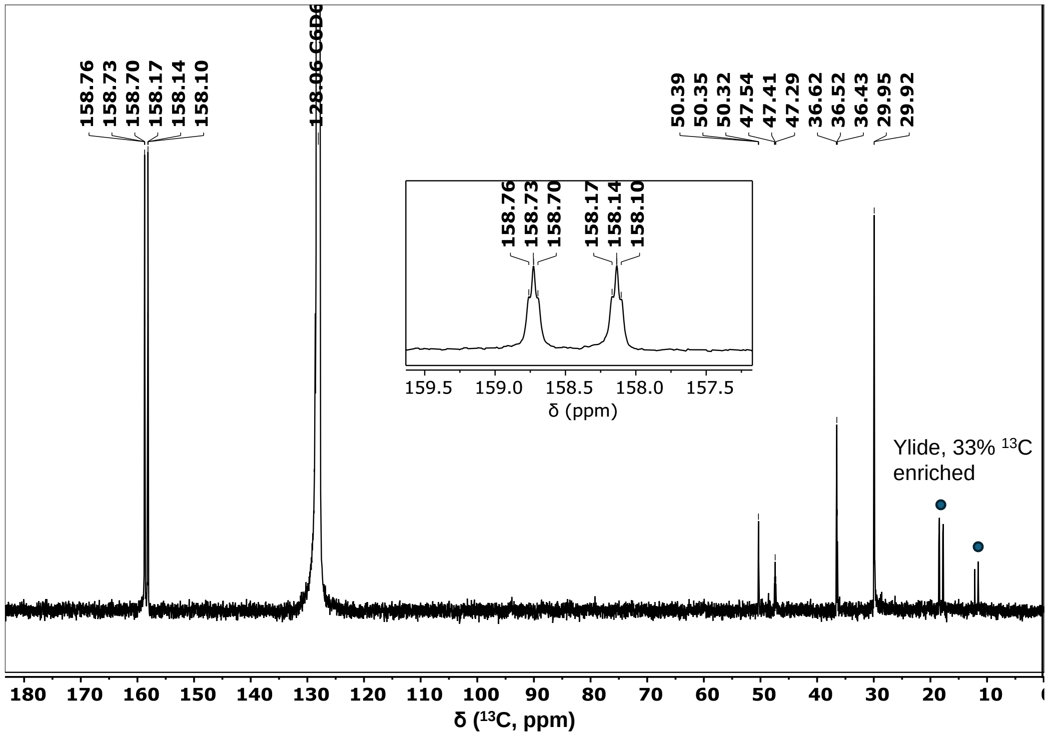

Figure S27. ^{13}C{^{1}H} NMR spectrum of 33% ^{13}C-enriched [(PCP)Ir(P=^{13}CH$_2$)] (**4-^{13}C**) in C_6D_6 (101 MHz), (traces of 33% ^{13}C-enriched Ph(^{13}CH$_3$)$_2$P=^{13}CHCO^{13}CH$_3$, at 19 and 11 ppm).

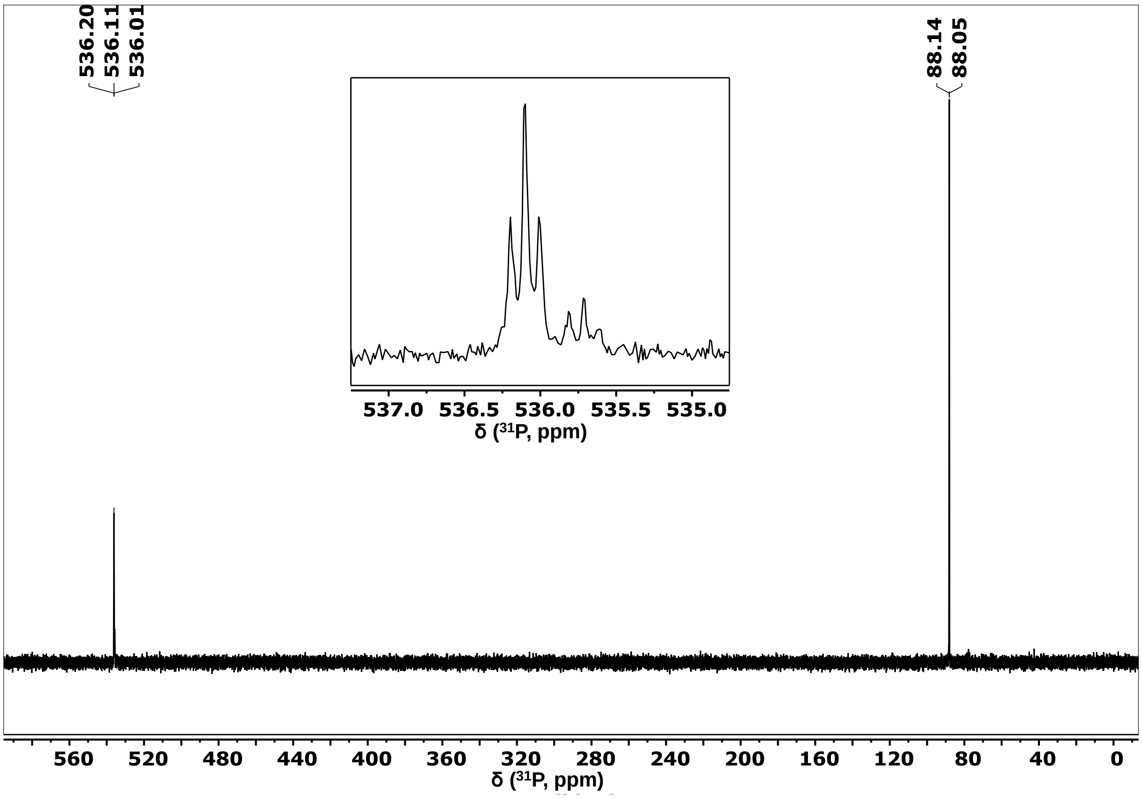

Figure S28. $^{31}P\{^{1}H\}$ NMR spectrum of 33% ^{13}C-enriched [(PCP)Ir(P=$^{13}CH_2$)] (**4-^{13}C**) in C_6D_6 (162 MHz). The central peak from the P=CH_2 ligand overlaps with one half of the ^{13}C satelite from P=$^{13}CH_2$, due to the chemical shift being isotopically shifted.

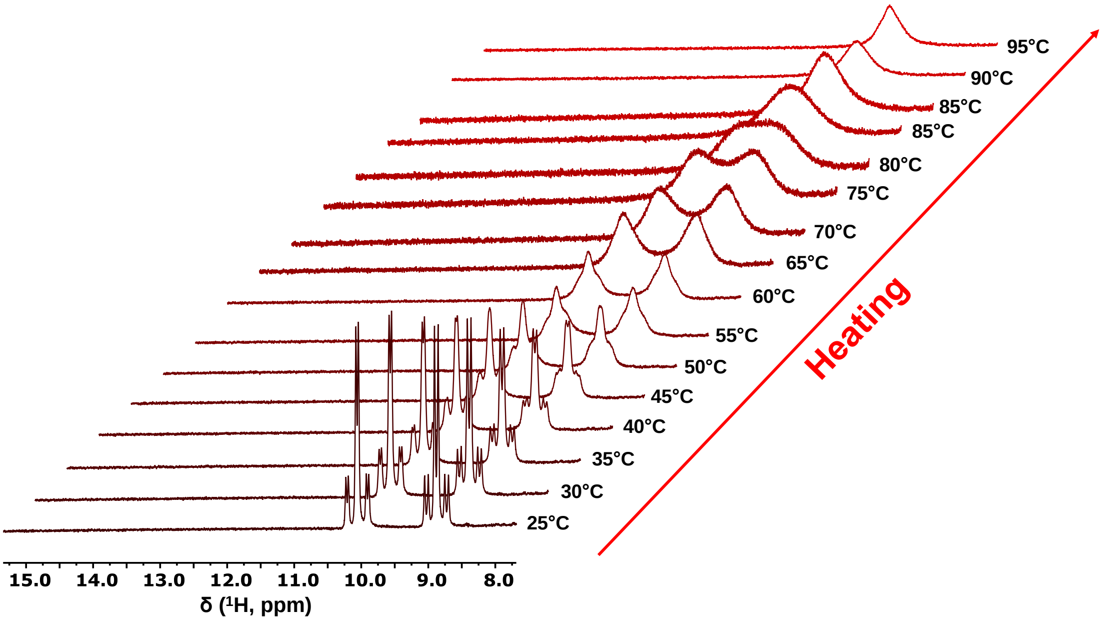

Figure S29. Variable temperature ^{1}H NMR spectra of 33% ^{13}C-enriched [(PCP)Ir(P=$^{13}CH_2$)] (**4-^{13}C**) showing resonances from the P=CH_2 ligand between +25 to +95 °C.

4.4.2 NMR Spectral Data for [(PCP)Ir(CO)](PCO)

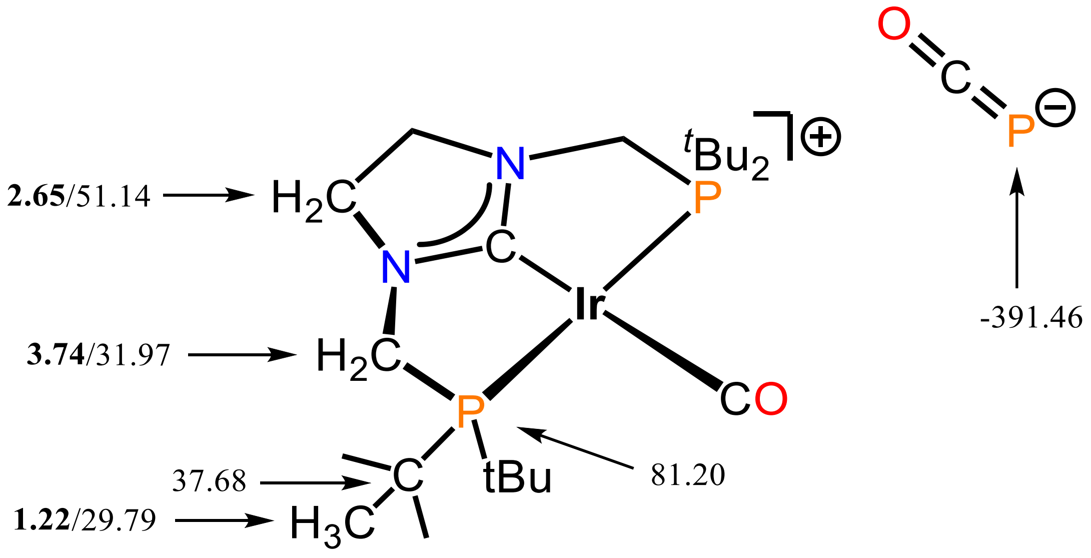

Figure S30. [(PCP)Ir(CO)](PCO) **^{1}H-**, ^{13}C-, and ^{31}P-NMR shifts.

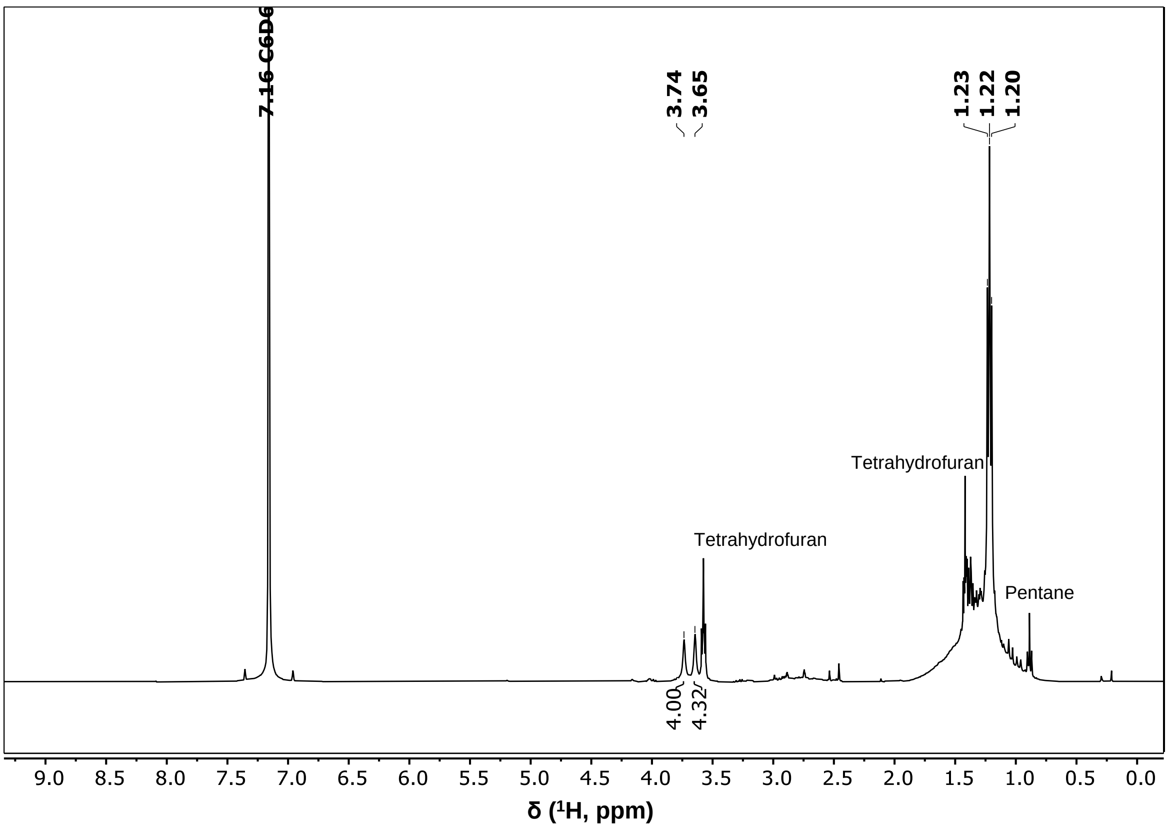

Figure S31. ^{1}H-NMR spectrum of [(PCP)Ir(CO)](PCO) in C_6D_6 (400 MHz). The resonances at 3.57 and 1.40 ppm (THF) and at 0.87 ppm (pentane) arise from traces of solvent.

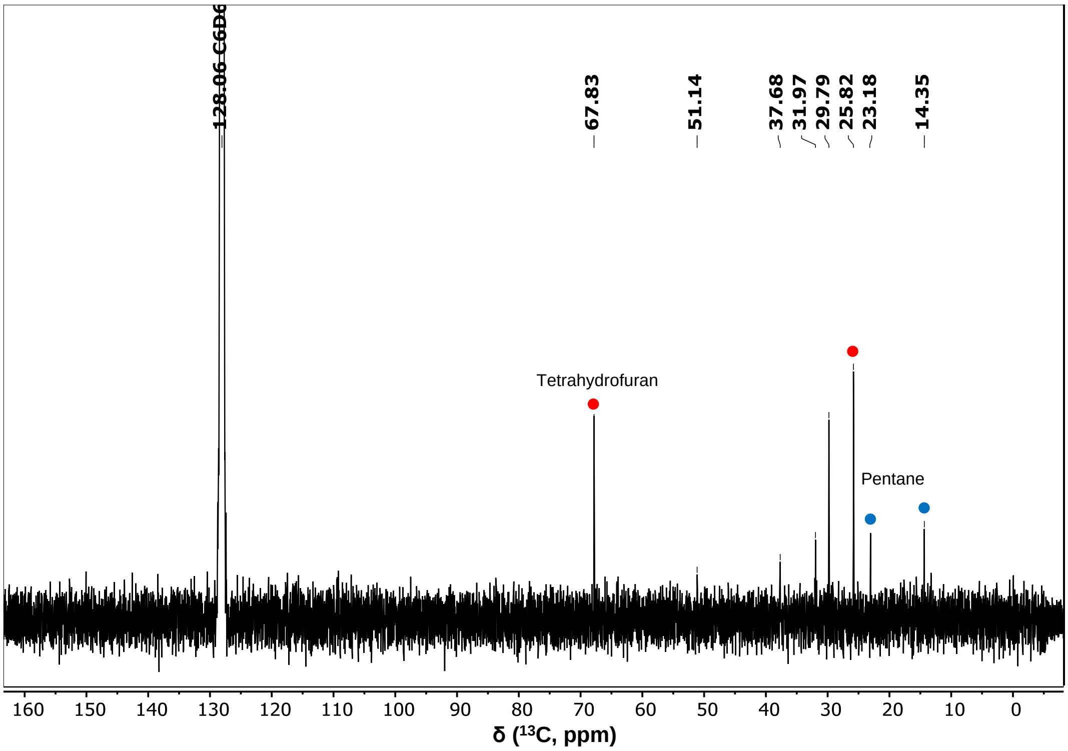

Figure S32. ^{13}C-NMR spectrum of [(PCP)Ir(CO)](PCO) in C_6D_6 (101 MHz) with traces of THF and pentane.

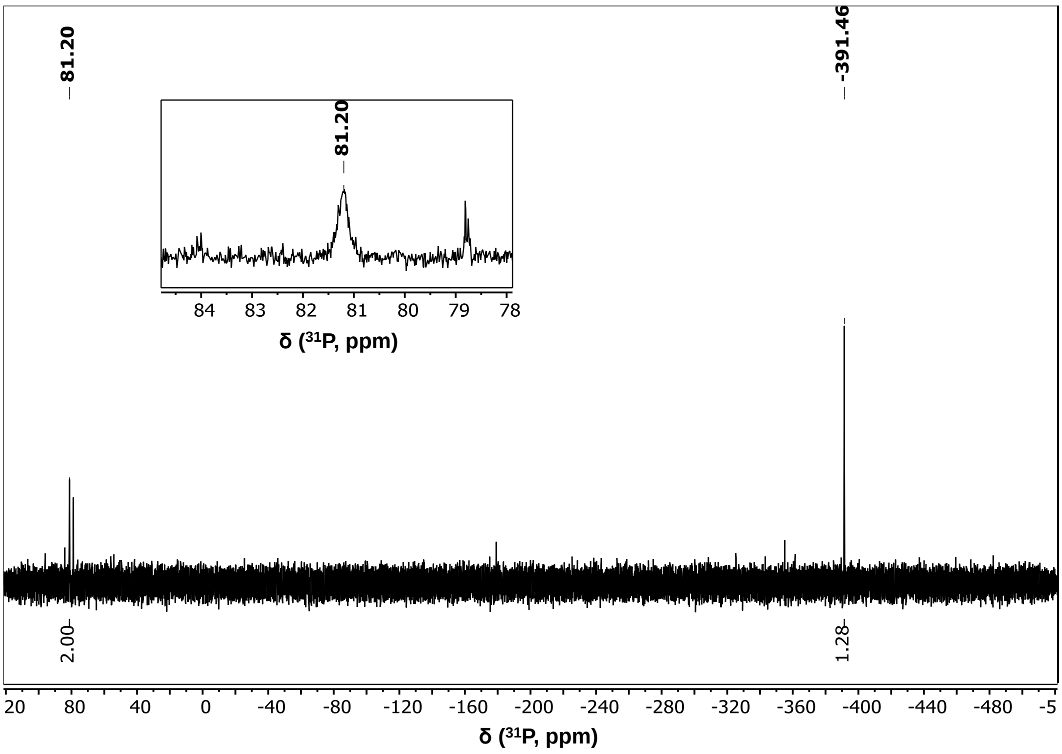

Figure S33. ^{31}P-NMR spectrum of [(PCP)Ir(CO)](PCO) in C_6D_6 (162 MHz).

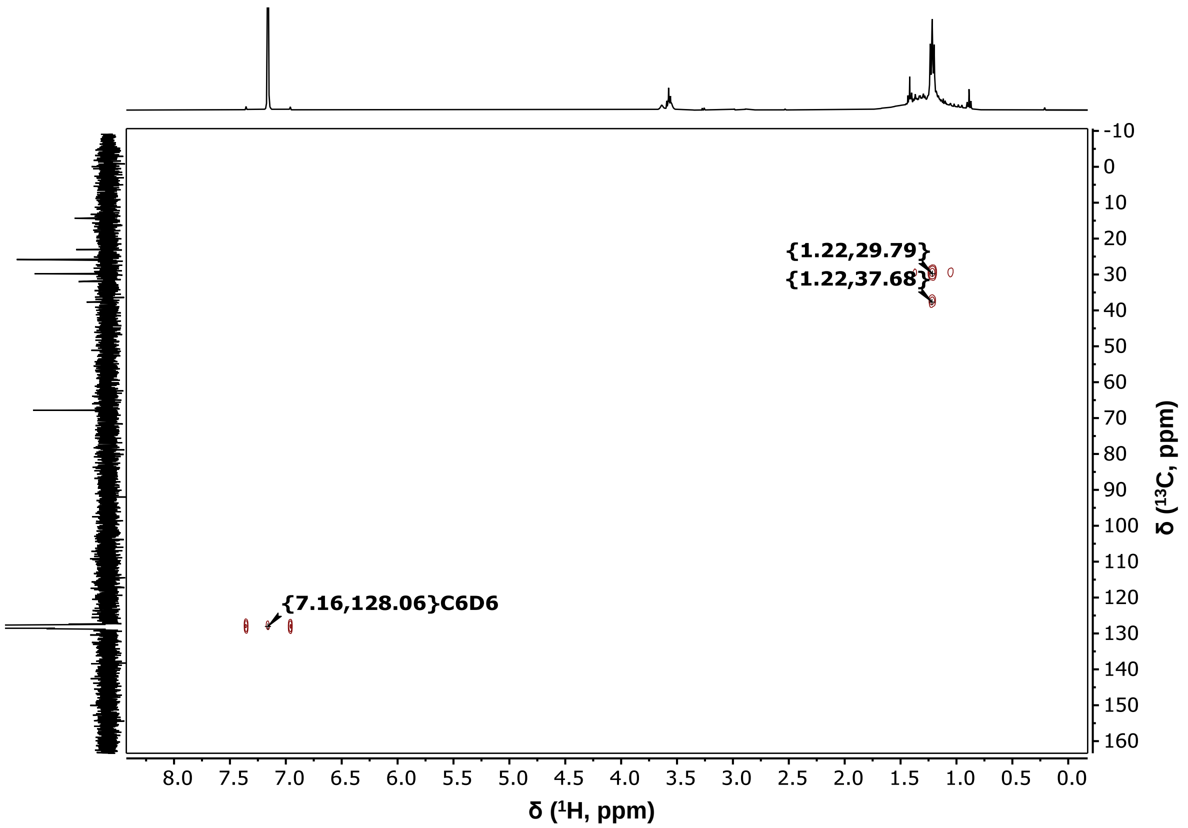

Figure S34. ^{1}H-^{13}C HMBC spectrum of [(PCP)Ir(CO)](PCO) in C_6D_6 (162 MHz).

4.4.3 NMR Spectral Data for Reaction Mixtures Forming [(PCP)Ir(P=CH_2)] (4); Methods A (Photolysis, $PhMe_2PCH_2$), B (Heating, $PhMe_2PCH_2$), and C (Photolysis, Ph_3PCH_2)

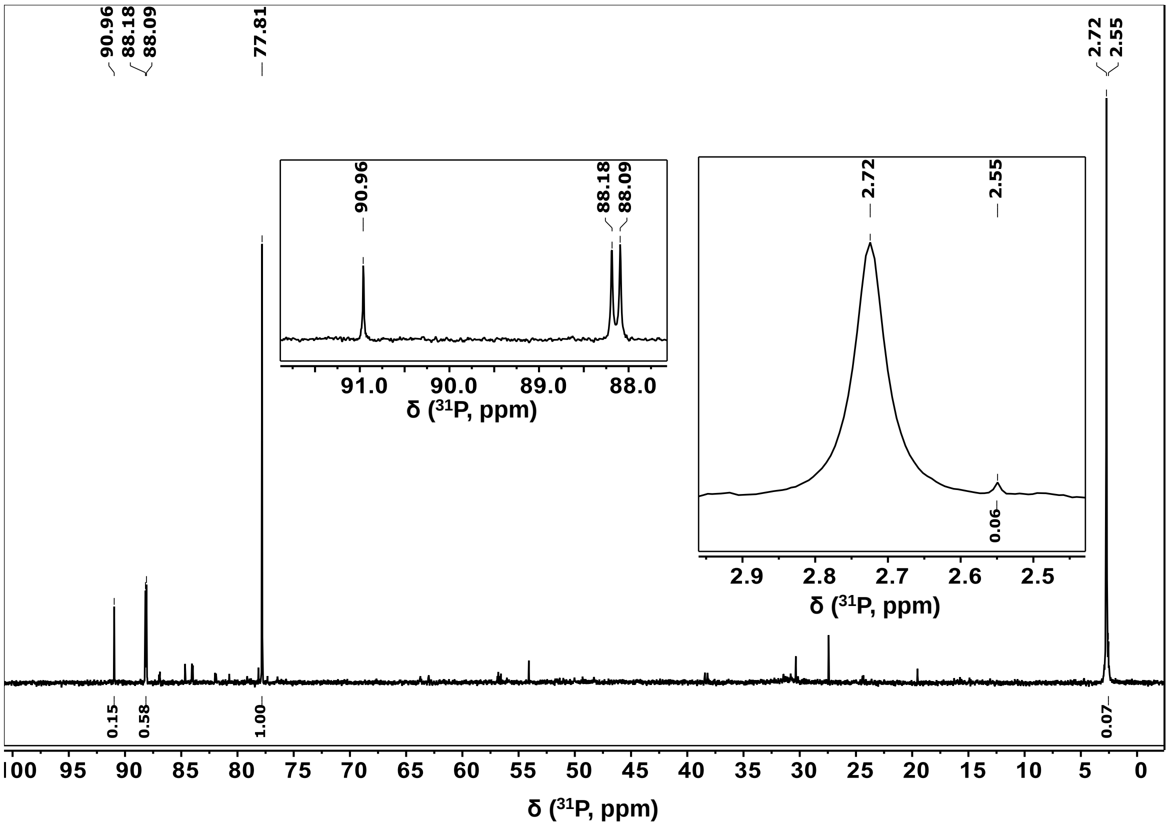

Figure S35. $^{31}P\{^1H\}$ NMR spectrum of reaction mixture from Method A (photolysis, **$PhMe_2PCH_2$**) (400 MHz, C_6D_6).

· [(PCP)IrCl], observed at 77.81 ppm was added as an internal reference (integral set to 1.00).

· [(PCP)IrCO](PCO), observed at 90.96 ppm (integrating to 0.15),

· [(PCP)Ir(P=CH_2)], observed at 88.09 ppm (integrating to 0.58),

· unconverted $PhMe_2P=CH_2$, observed at 2.72 ppm (integrating to 3.4),

· $PhMe_2P=CHCOMe$, observed at 2.55 ppm (integrating to 0.06).

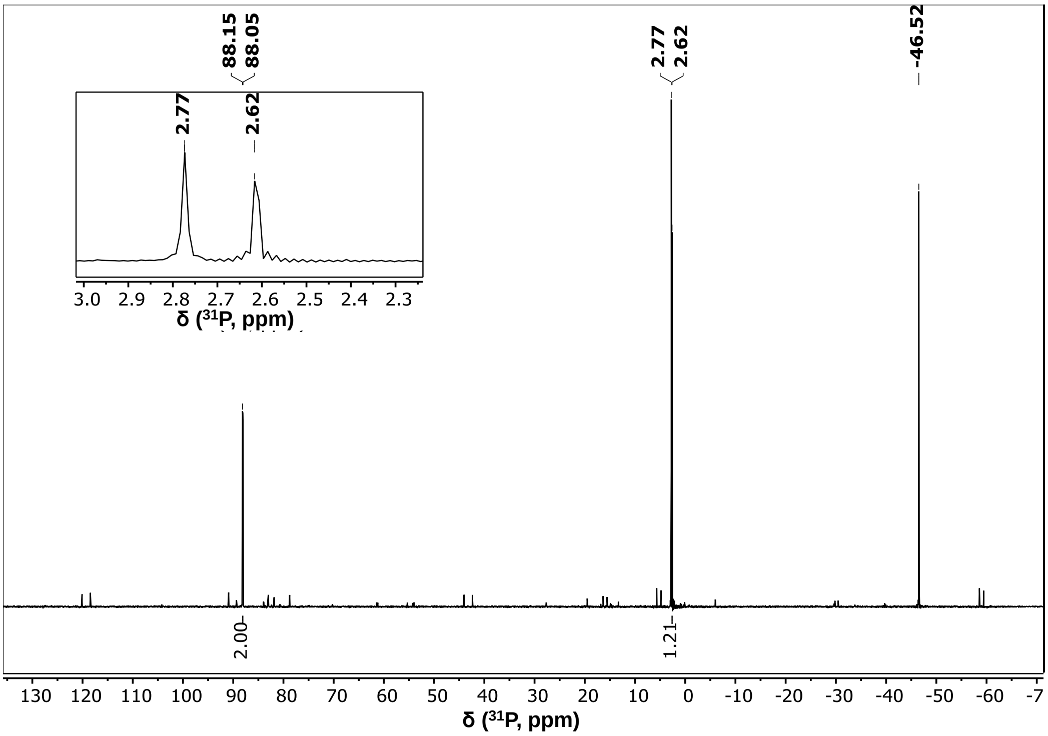

Figure S36. $^{31}P\{^{1}H\}$ NMR spectrum of reaction mixture from Method B (heating, **$PhMe_2PCH_2$**) (400 MHz, C_6D_6).

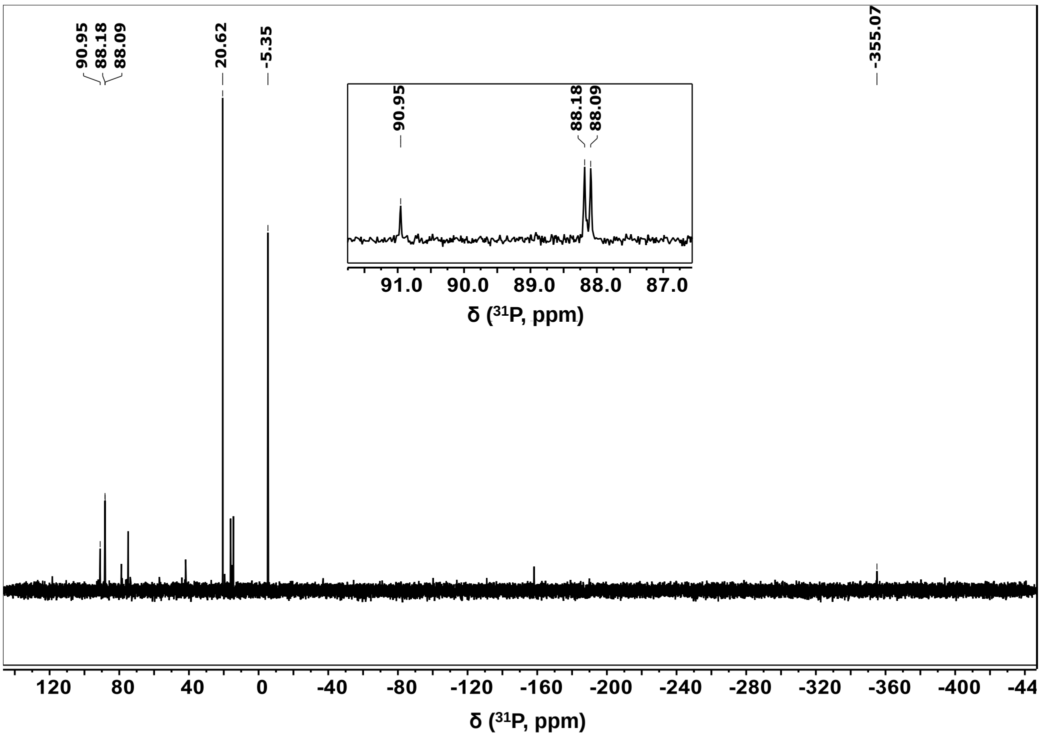

Figure S37. $^{31}P\{^{1}H\}$ NMR spectrum of reaction mixture from Method C (photolysis, **Ph_3PCH_2**) (400 MHz, C_6D_6), the corresponding [(PCP)IrCO](PCO) byproduct at 90.95 and -355.07 ppm, unconverted $Ph_3P{=}CH_2$ at 20.62 ppm and Ph_3P at -5.35 ppm .

4.5 NMR Spectral Data for $PhMe_2P{=}CHCOMe$

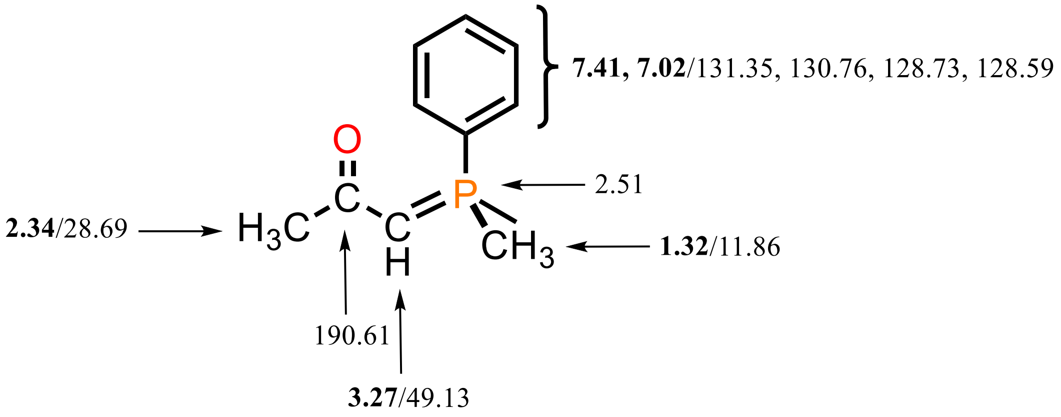

Figure S38. $PhMe_2P{=}CHCOMe$ **¹H-**, ^{13}C-, and ^{31}P-NMR shifts.

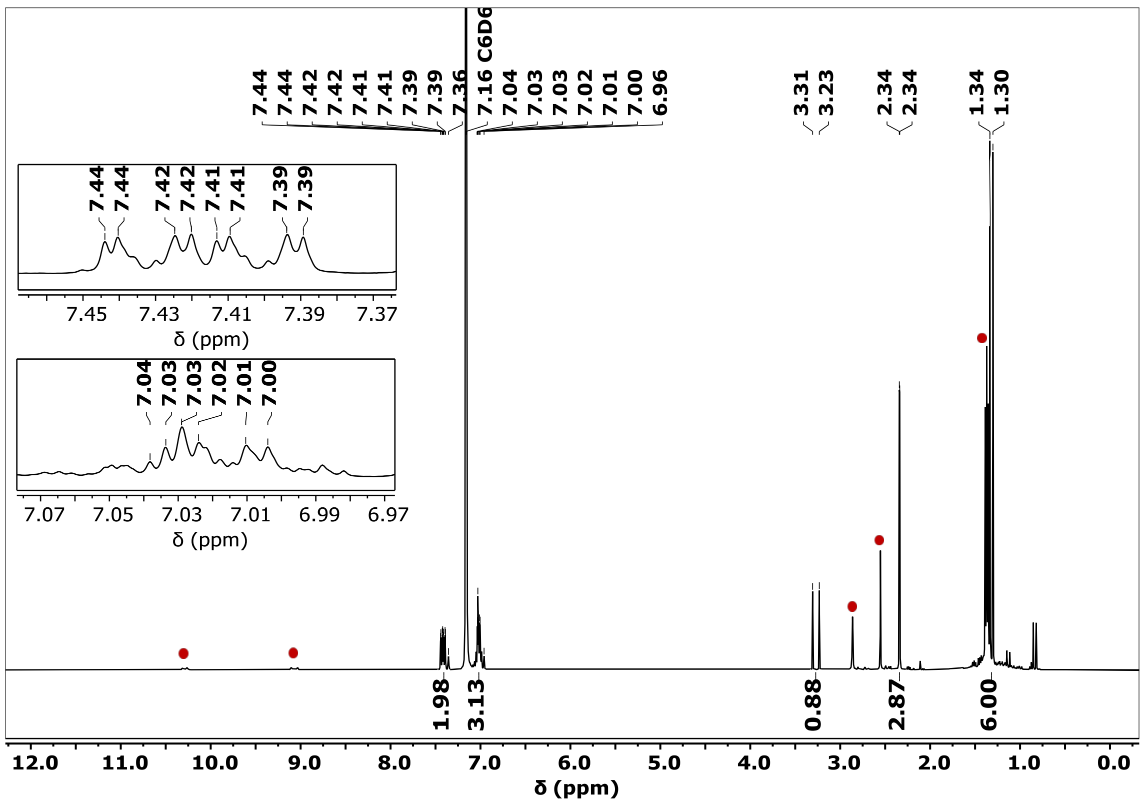

Figure S39. ^{1}H NMR spectrum of $PhMe_2P{=}CHCOMe$ in C_6D_6 (400 MHz) with traces of complex (**4**) as red dots.

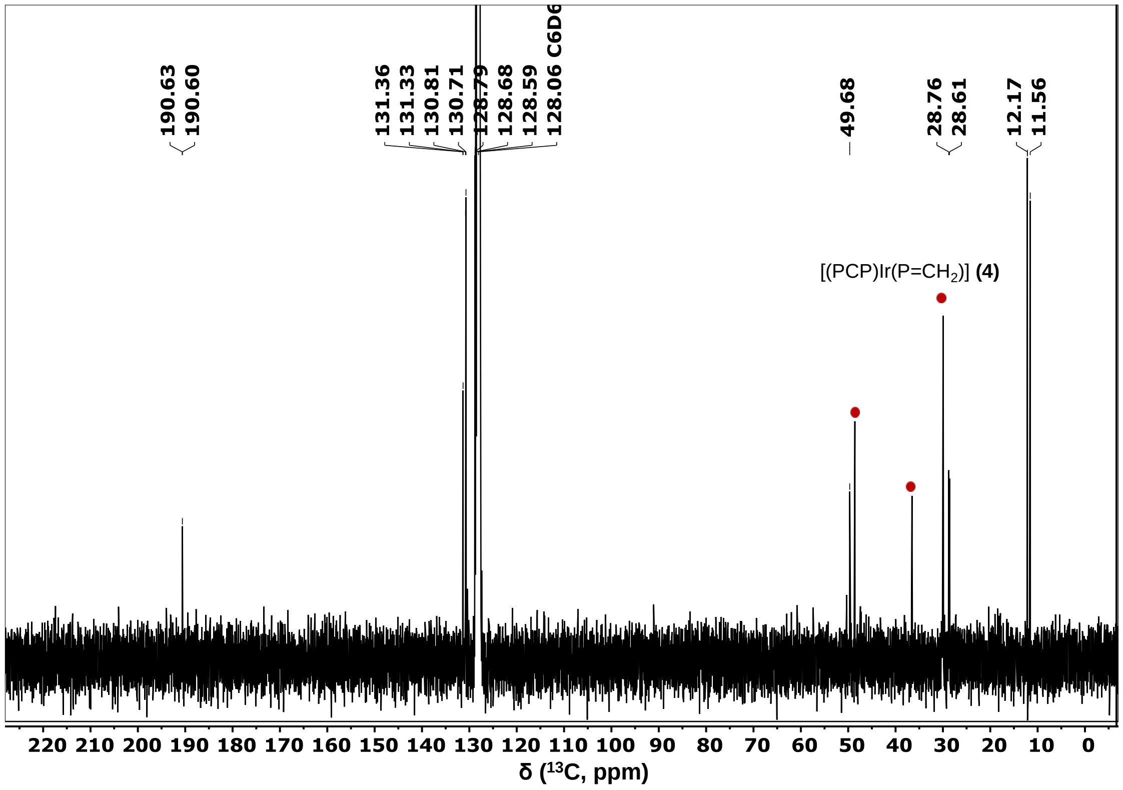

Figure S40. $^{13}C\{^1H\}$ NMR spectrum of $PhMe_2P{=}CHCOMe$ in C_6D_6 (101 MHz).

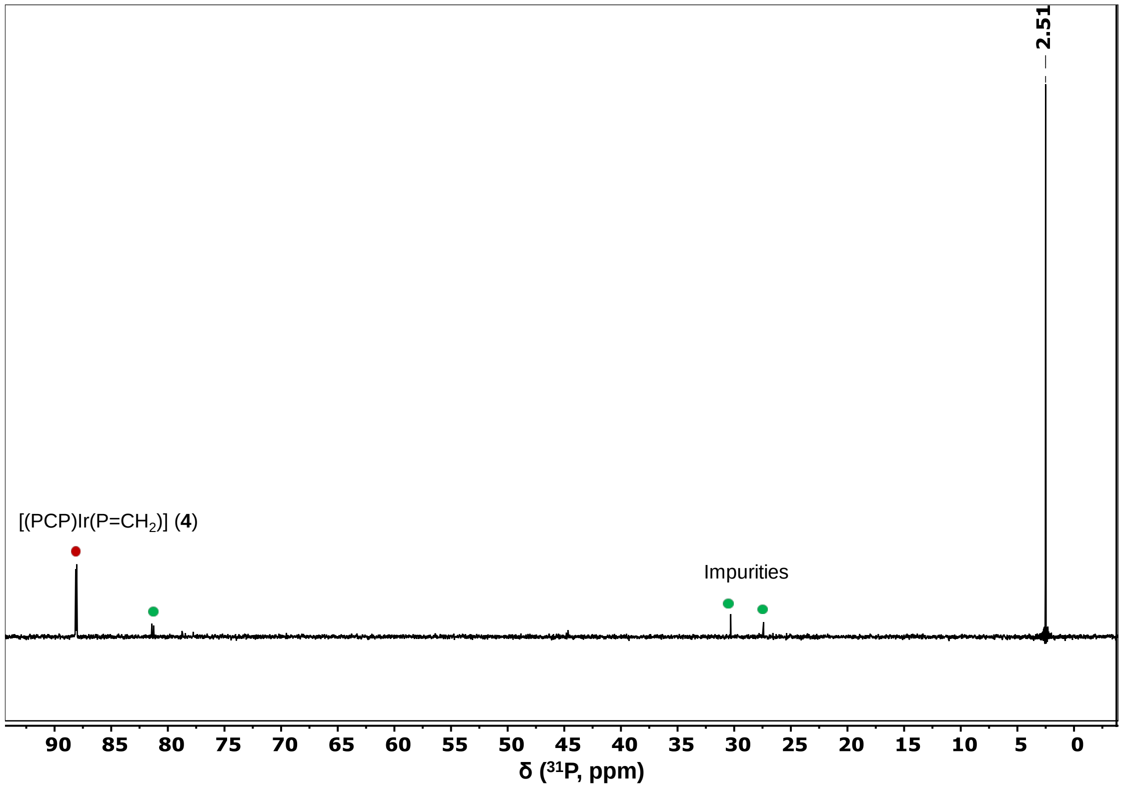

Figure S41. $^{31}P\{^1H\}$-NMR spectrum of $PhMe_2P{=}CHCOMe$ in C_6D_6 (162 MHz).

4.5.1 NMR Spectral Data for 33% ^{13}C-enriched Ph($^{13}CH_3$)$_2$P=^{13}CHCO$^{13}CH_3$

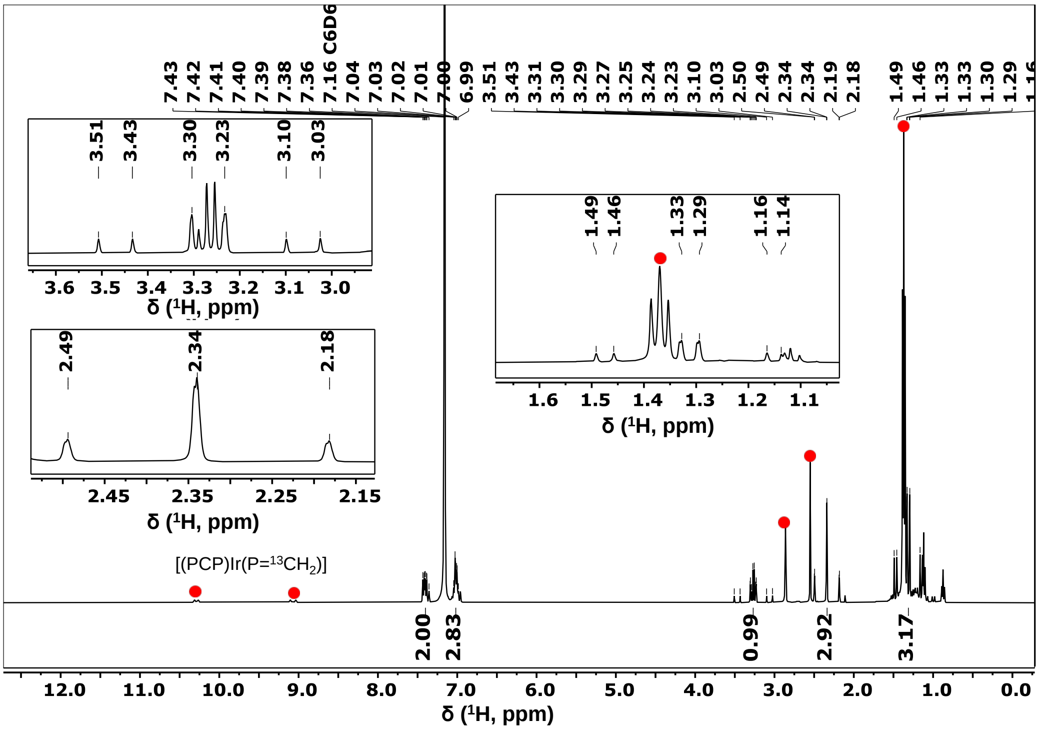

Figure S42. ^{1}H NMR spectrum of 33% ^{13}C-enriched Ph($^{13}CH_3$)$_2$P=^{13}CHCO$^{13}CH_3$ in C_6D_6 (400 MHz). The resonances at 3.26 and 1.12 ppm (diethyl ether) and at 0.87 and 1.22 ppm (pentane) stem from a trace of solvent.

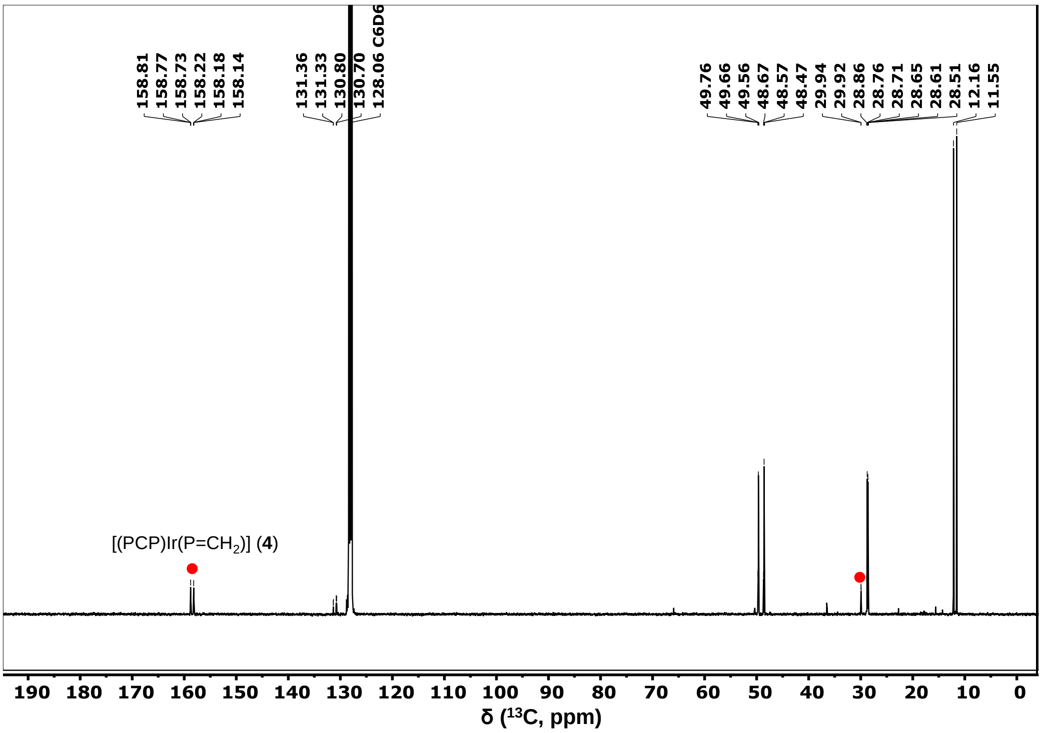

Figure S43. ^{13}C{^{1}H} NMR spectrum of 33% ^{13}C-enriched Ph($^{13}CH_3$)$_2$P=^{13}CHCO$^{13}CH_3$ in C_6D_6 (101 MHz). The resonances at 65.92 and 15.60 ppm (diethyl ether) and at 34.44, 22.73 and, 14.28 ppm (pentane) stem from a trace of solvent.

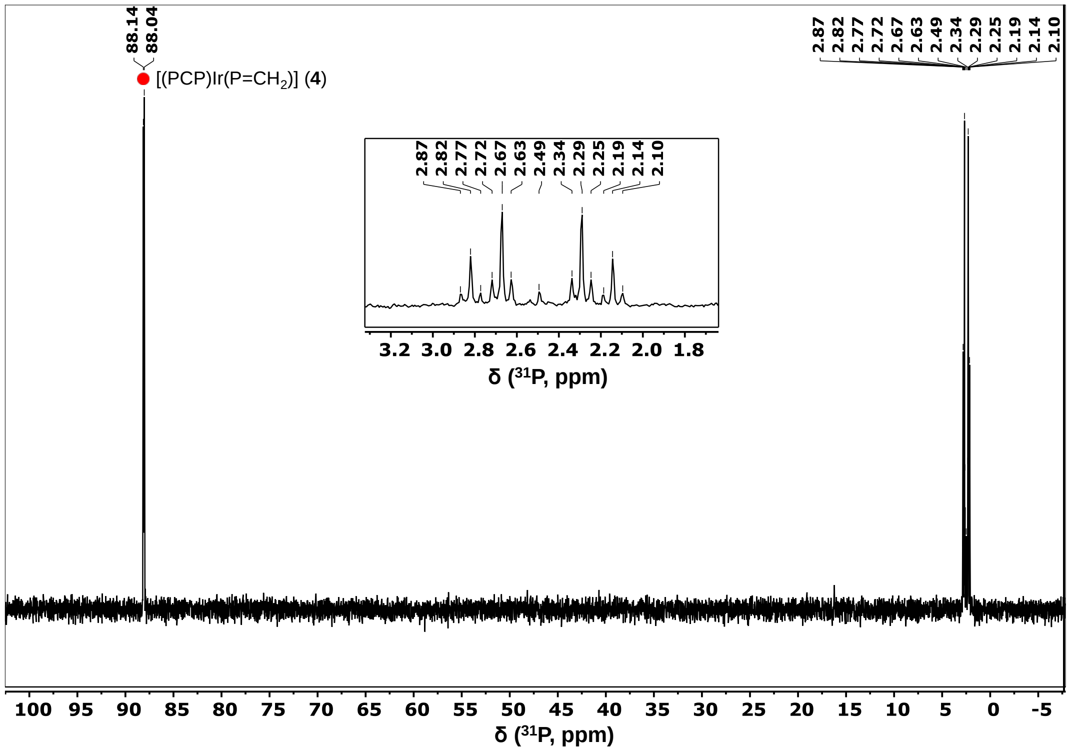

Figure S44. $^{31}P\{^1H\}$ NMR spectrum of 33% ^{13}C-enriched $Ph(^{13}CH_3)_2P{=}^{13}CHCO^{13}CH_3$ in C_6D_6 (162 MHz). A trace of **4**-^{13}C is observed at 88.09 ppm.

4.6 NMR Spectral Data for [(PCP)Ir{P(=CH$_2$)(NAd)}] (5)

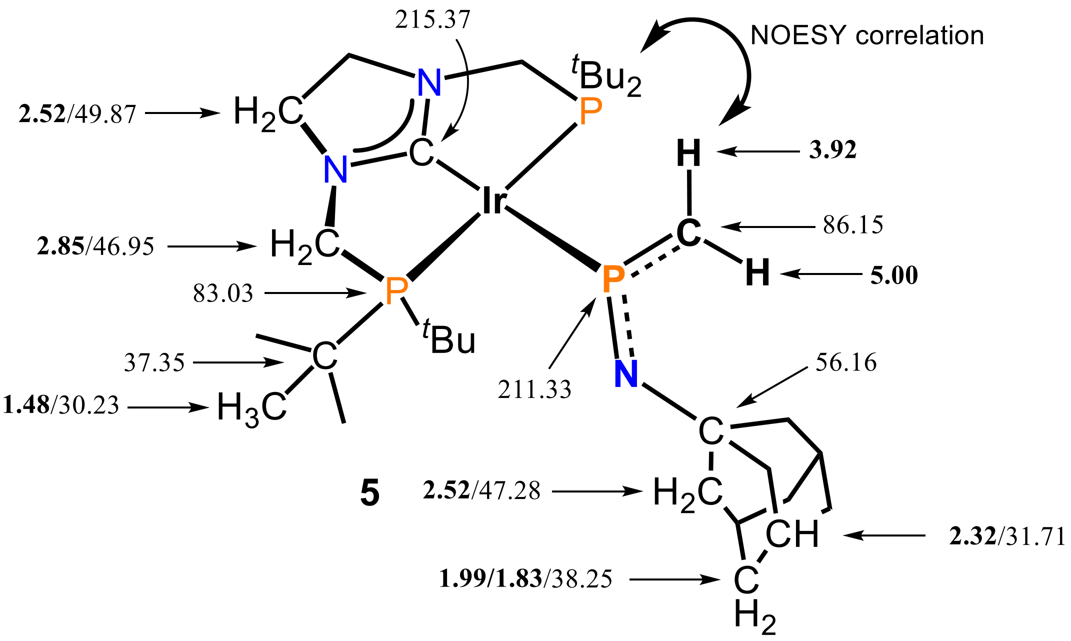

Figure S45. [(PCP)Ir{P(=CH$_2$)(NAd)}] (**5**) **^{1}H-**, ^{13}C-, and ^{31}P-NMR shifts.

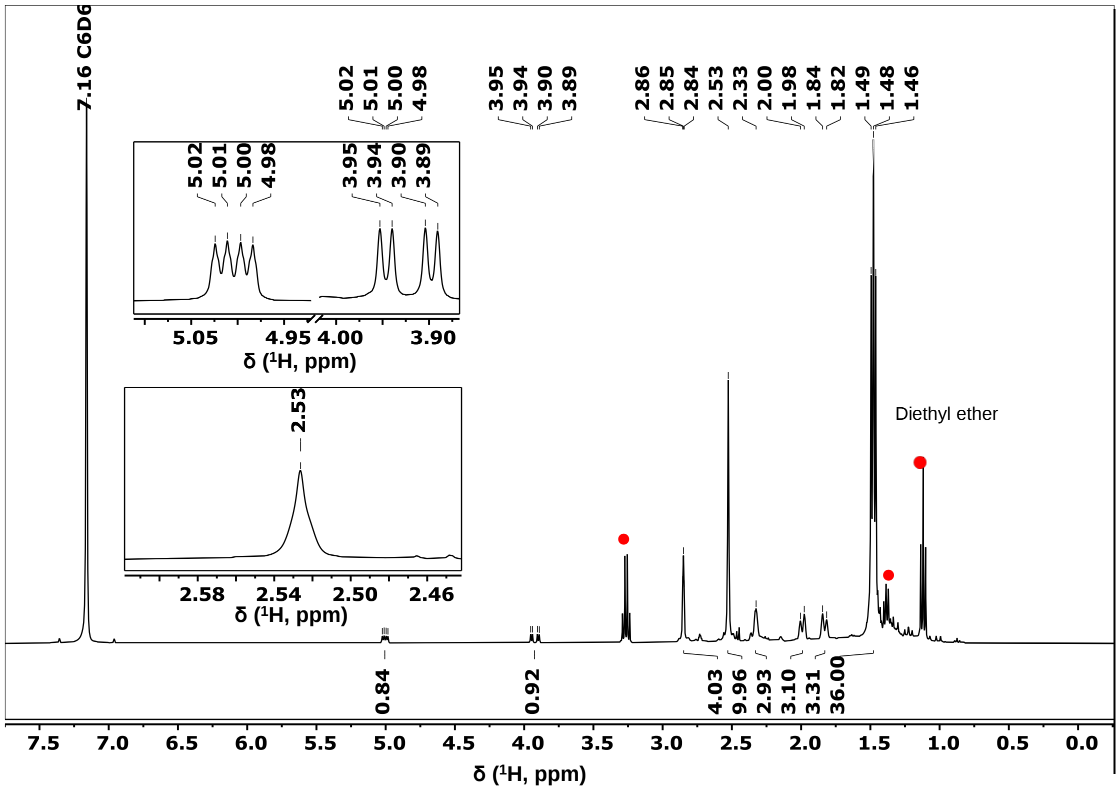

Figure S46. ^{1}H-NMR spectrum of [(PCP)Ir{P(=CH$_2$)(NAd)}] (**5**) in C_6D_6 (500 MHz). The resonances at 3.26 and 1.12 ppm (diethyl ether) stem from a trace of solvent.

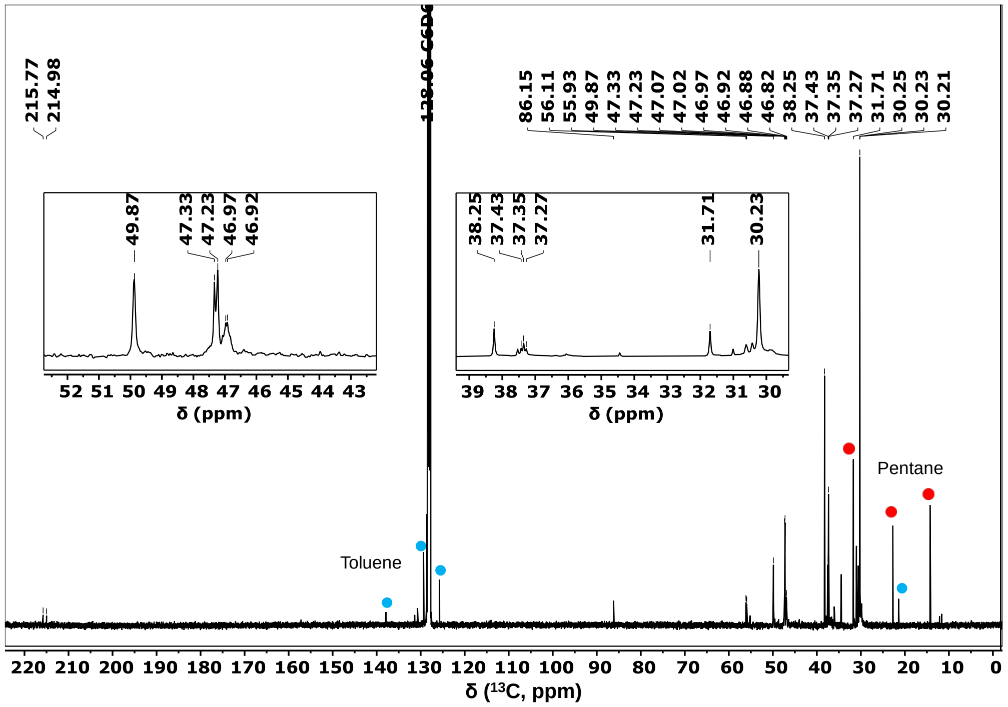

Figure S47. $^{13}C\{^1H\}$-NMR spectrum of [(PCP)Ir{P(=CH_2)(NAd)}] (**5**) in C_6D_6 (126 MHz). The resonances at 139.9, 129.3, 128.5, 125.7 and 21.43 ppm (toluene) and at 34.44, 22.73 and, 14.28 ppm (pentane) stem from a trace of solvent.

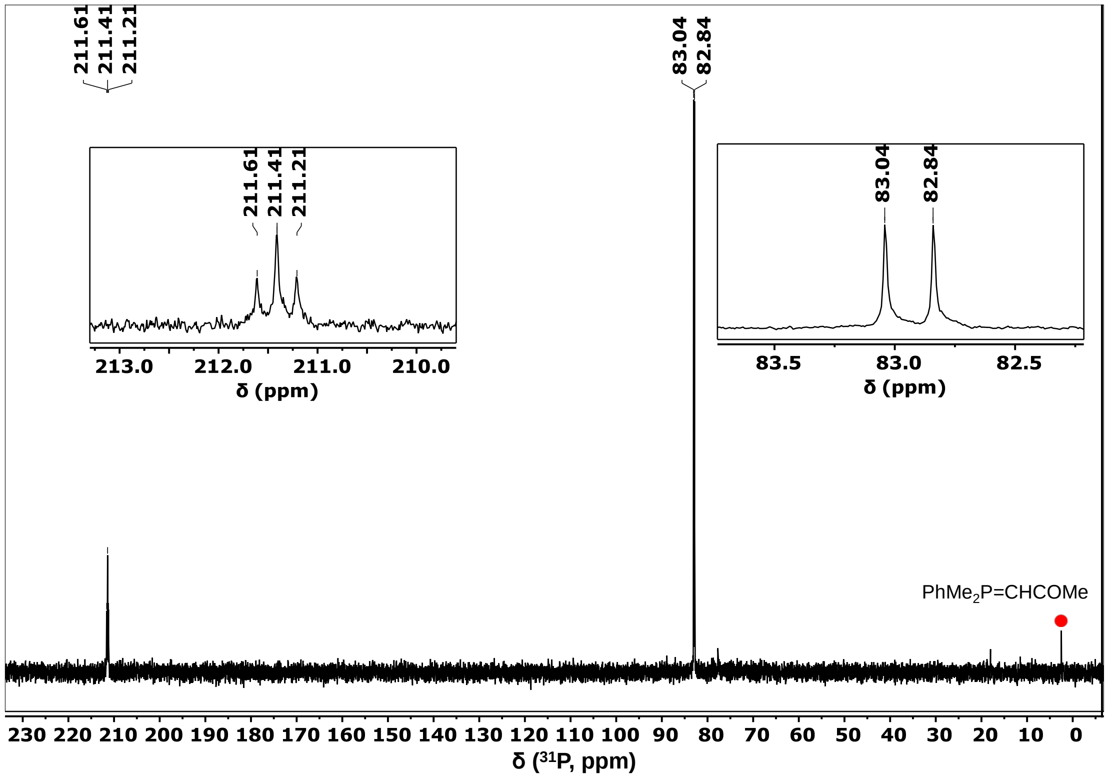

Figure S48. $^{31}P\{^1H\}$ NMR spectrum of [(PCP)Ir{P(=CH_2)(NAd)}] (**5**) in C_6D_6 (203 MHz).

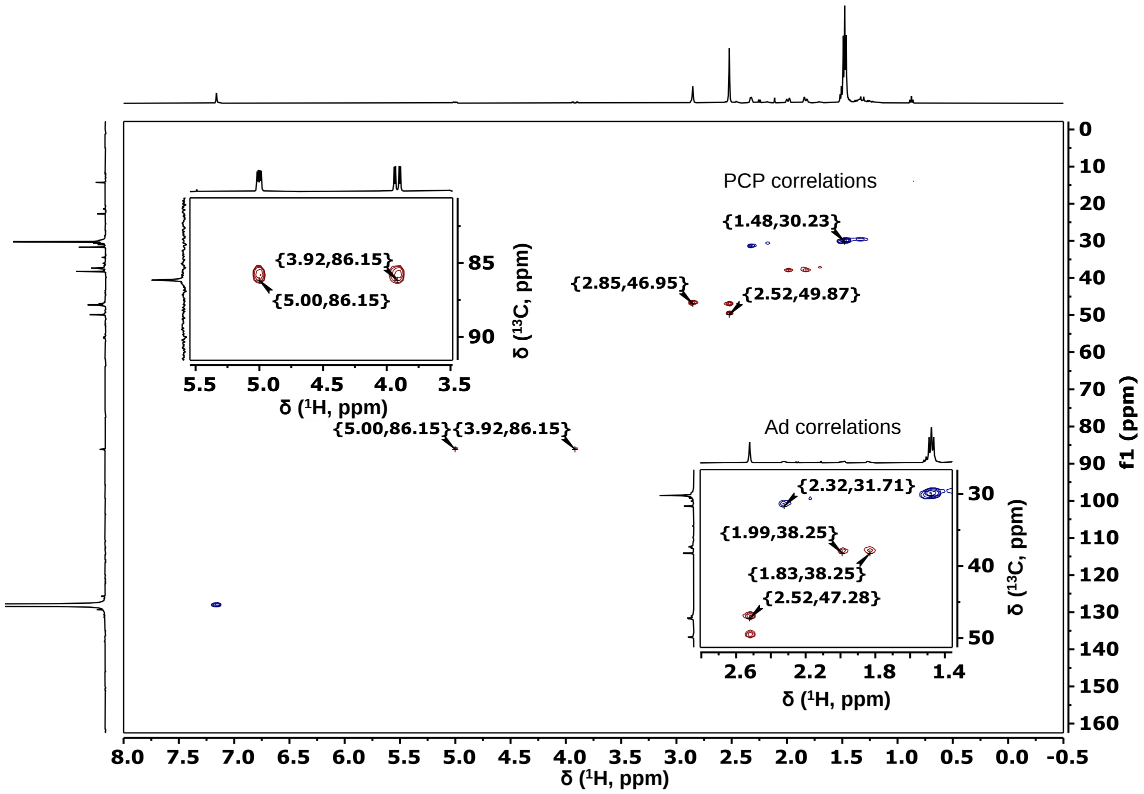

Figure S49. ^{1}H-^{13}C HSQC NMR spectrum of [(PCP)Ir{P(=CH_2)(NAd)}] (**5**) in C_6D_6 (500 MHz).

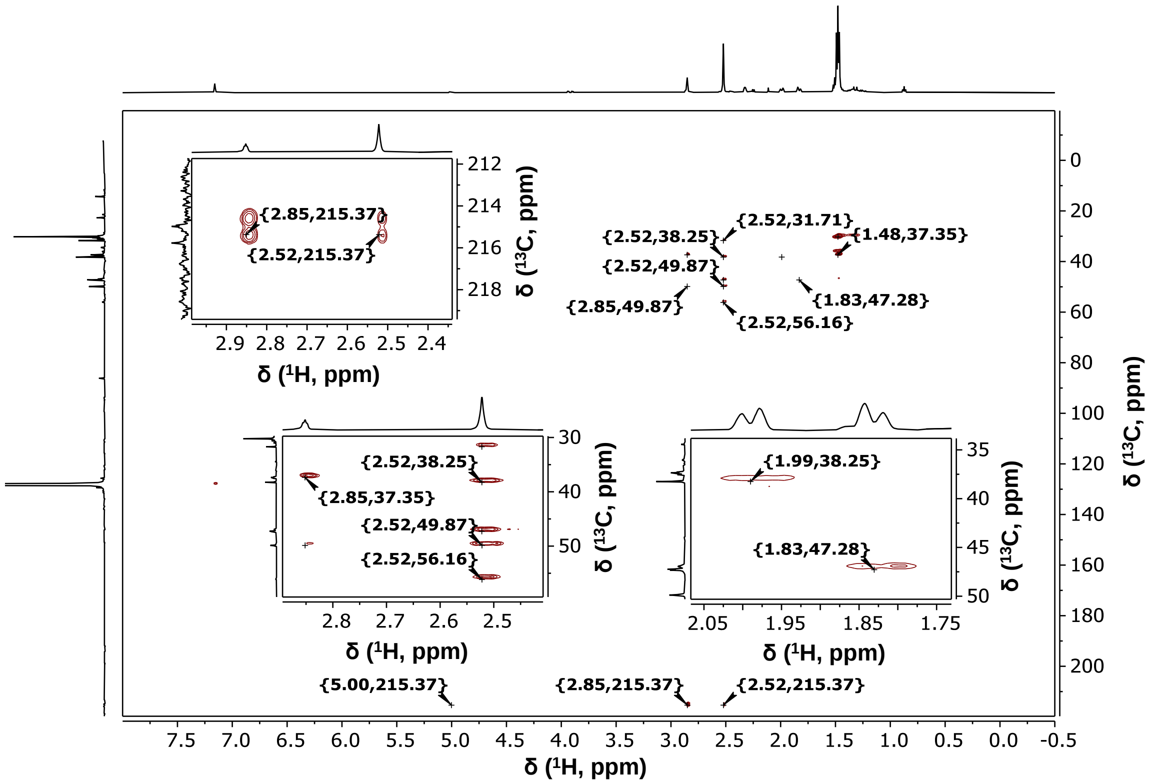

Figure S50. ^{1}H-^{13}C HMBC NMR spectrum of [(PCP)Ir{P(=CH_2)(NAd)}] (**5**) in C_6D_6 (500 MHz).

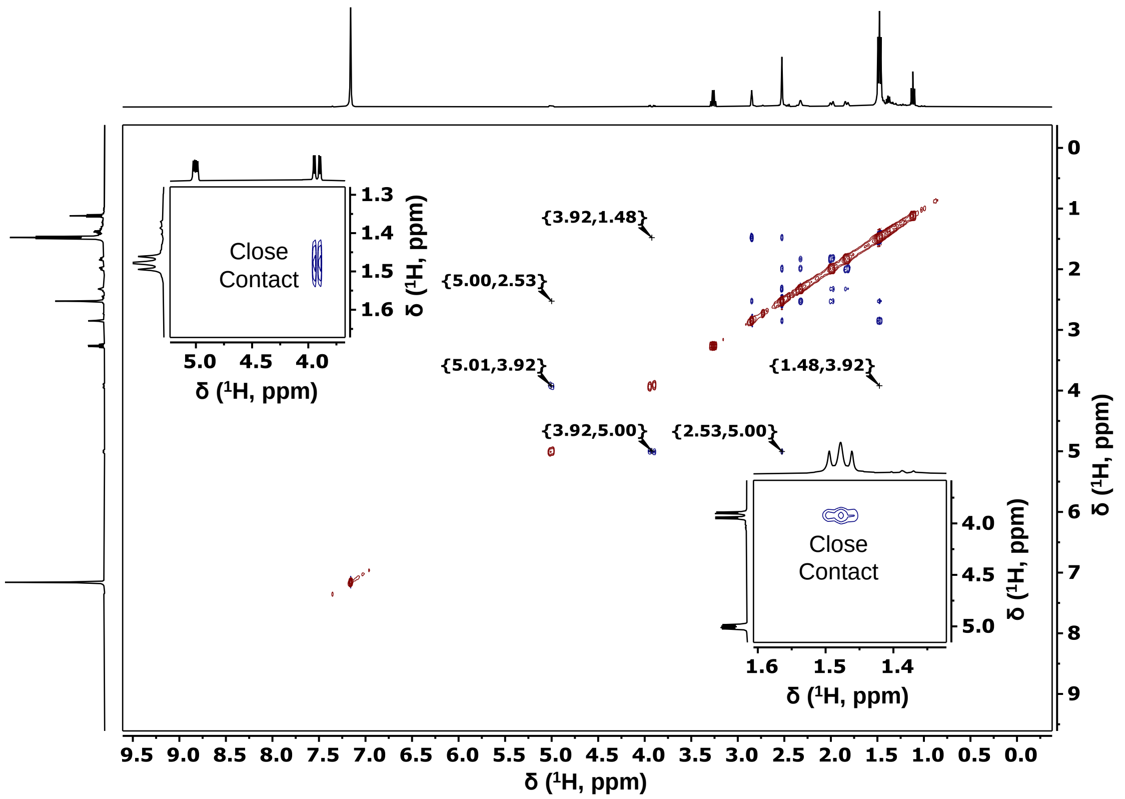

Figure S51. NOESY NMR spectrum of [(PCP)Ir{P(=CH_2)(NAd)}] (**5**) in C_6D_6 (400 MHz).

4.6.1 NMR Spectral Data for 33% ^{13}C-enriched [(PCP)Ir{P(=^{13}CH$_2$)(NAd)}] (5-^{13}C)

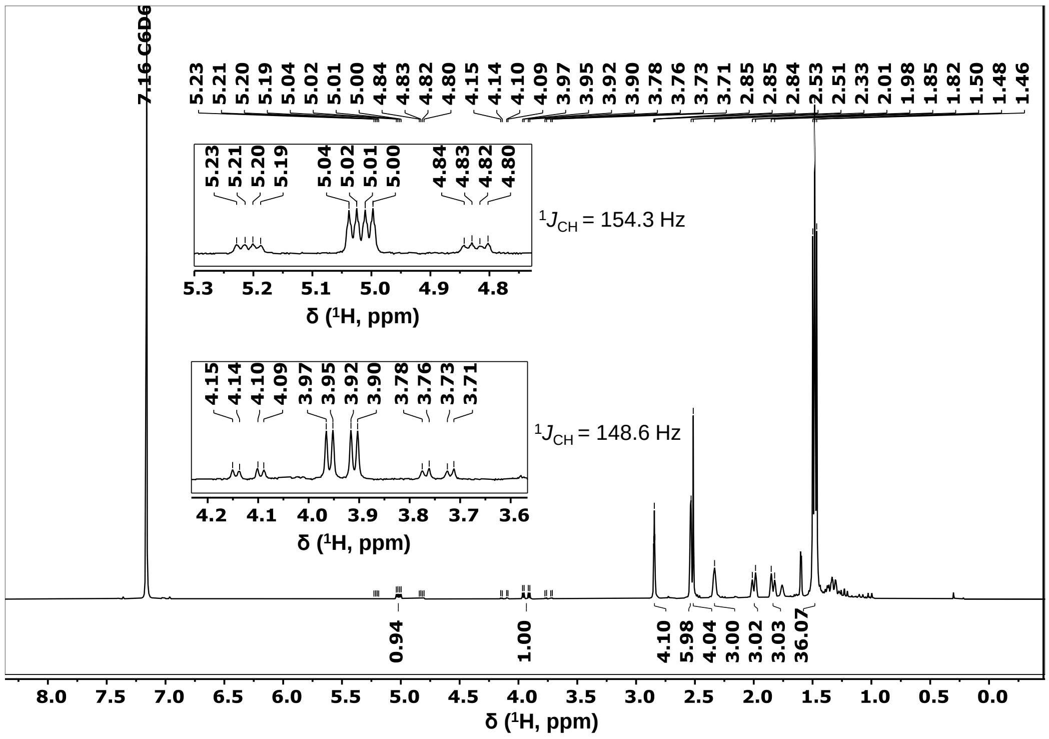

Figure S52. ^{1}H NMR spectrum of 33% ^{13}C-enriched [(PCP)Ir{P(=^{13}CH$_2$)(NAd)}] (**5-^{13}C**) in C_6D_6 (400 MHz).

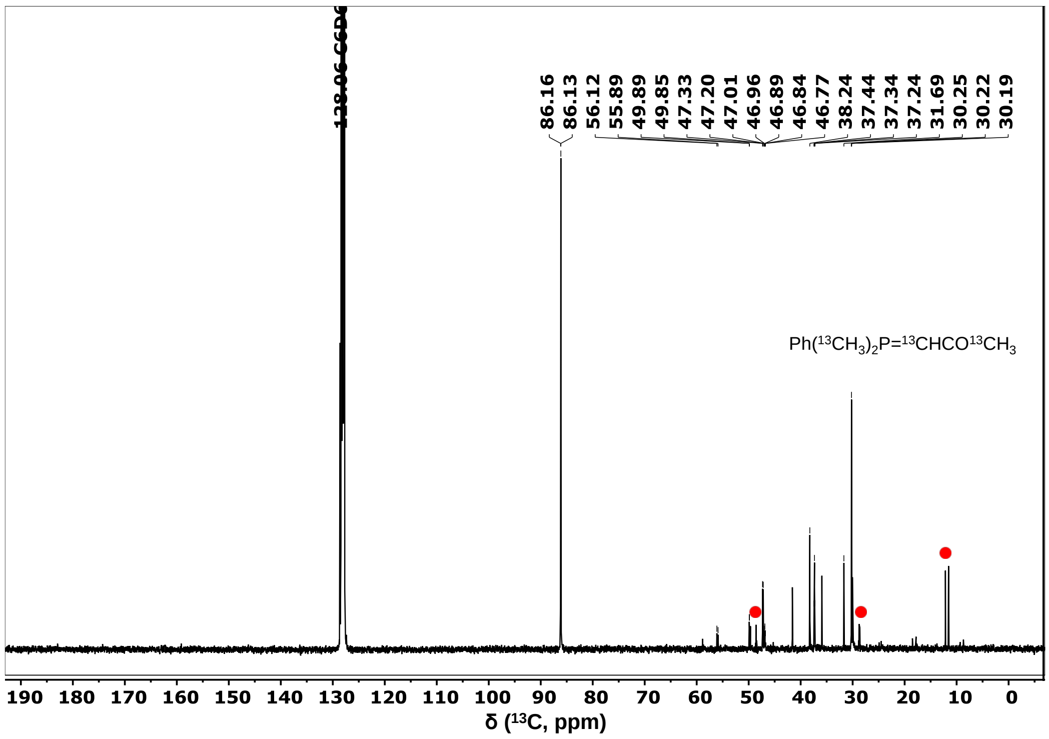

Figure S53. ^{13}C{^{1}H} NMR spectrum of 33% ^{13}C-enriched [(PCP)Ir{P(=^{13}CH$_2$)(NAd)}] (**5-^{13}C**) in C_6D_6 (101 MHz)

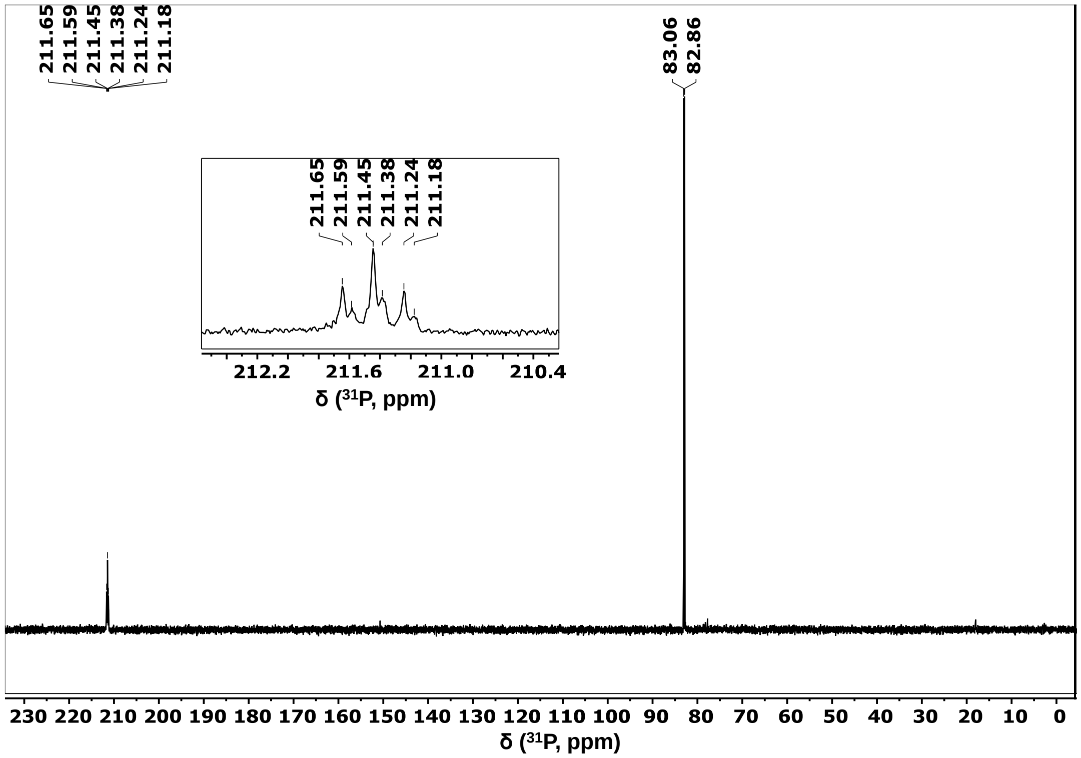

Figure S54. $^{31}P\{^{1}H\}$ NMR spectrum of 33% ^{13}C-enriched [(PCP)Ir{P(=$^{13}CH_2$)(NAd)}] (**5-^{13}C**) in C_6D_6 (162 MHz). The central peak overlaps with the ^{13}C satelites, due to the small P–C coupling constant ($^{1}J_{PC}$ = 3.4 Hz).

5 IR Spectroscopy

5.1 IR Spectral Data for [(PCP)IrCl] (1).

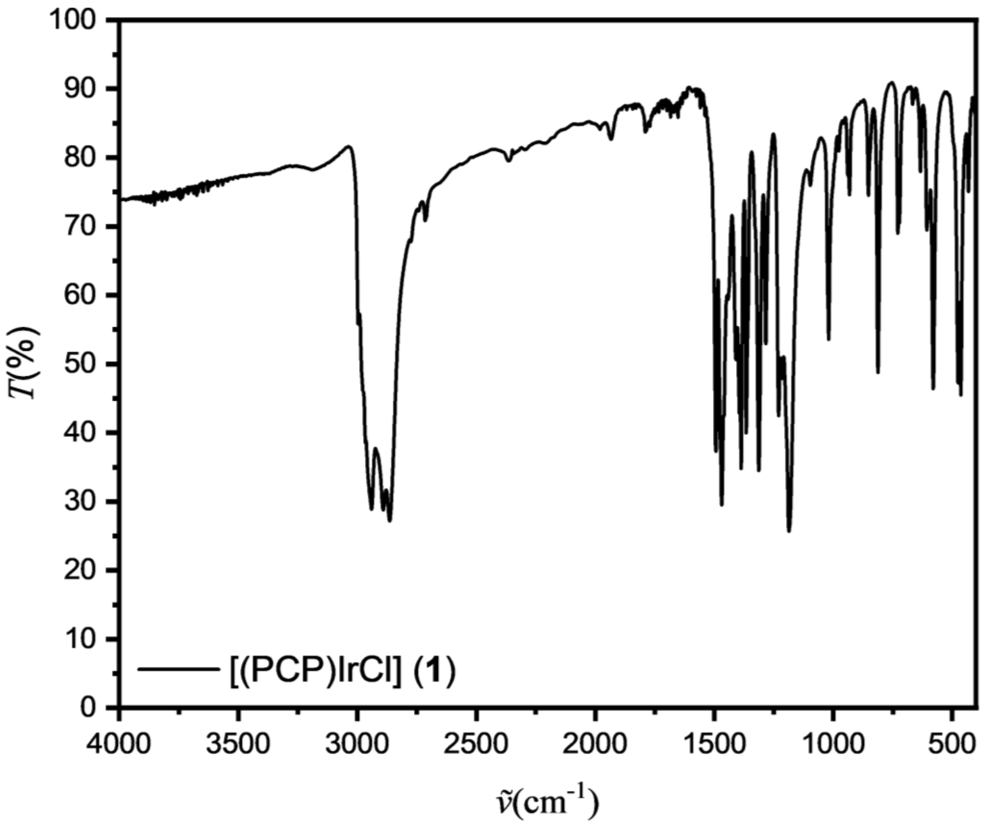

Figure S55. IR spectrum of solid [(PCP)IrCl] (**1**) pressed into KBr matrix.

5.2 IR Spectral Data for [(PCP)Ir(PCO)] (2).

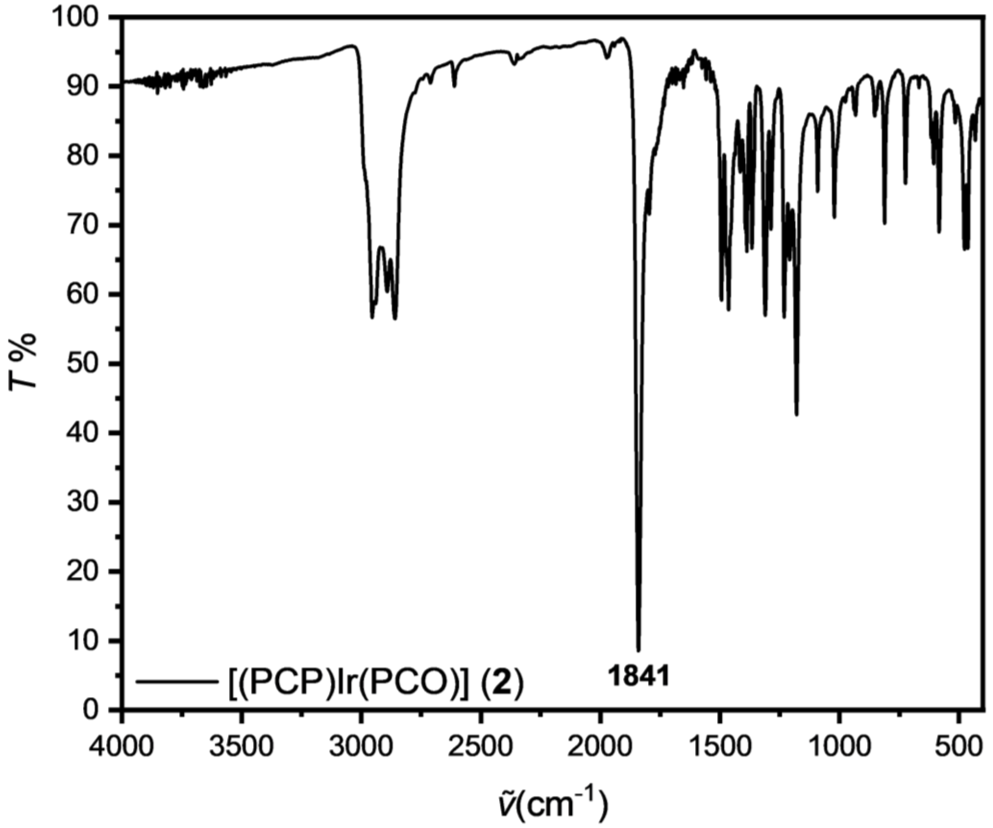

Figure S56. IR spectrum of solid [(PCP)Ir(PCO)] (**2**) pressed into KBr matrix.

5.3 IR Spectral Data comparison between [(PCP)IrCl] (1) and [(PCP)Ir(PCO)] (2)

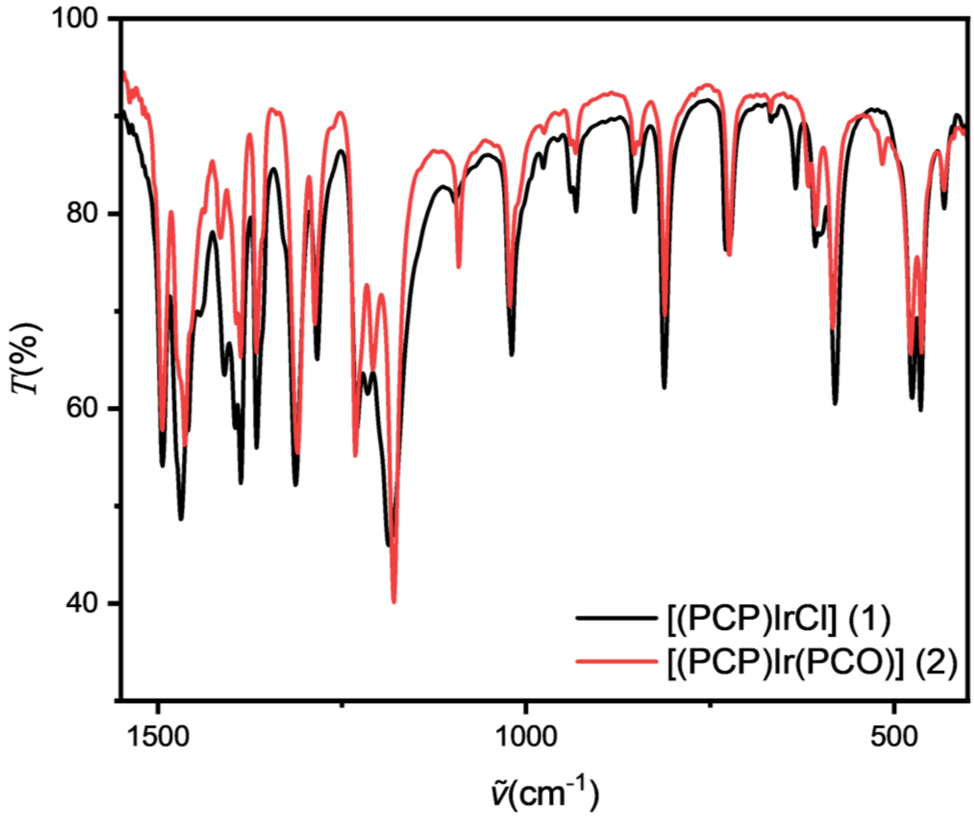

Figure S57. Overlay of IR spectra of solid [(PCP)IrCl] (**1**) and [(PCP)Ir(PCO)] (**2**) pressed into KBr matrices and zoomed in on the fingerprint region. A resonance at 1092 cm^{-1} for **2** can be assigned as a symmetrical PCO mode.

5.4 IR Spectral Data for [{(PCP)(OC)Ir}$_2$(η^2,η^2;μ_2-P_2)] (3)

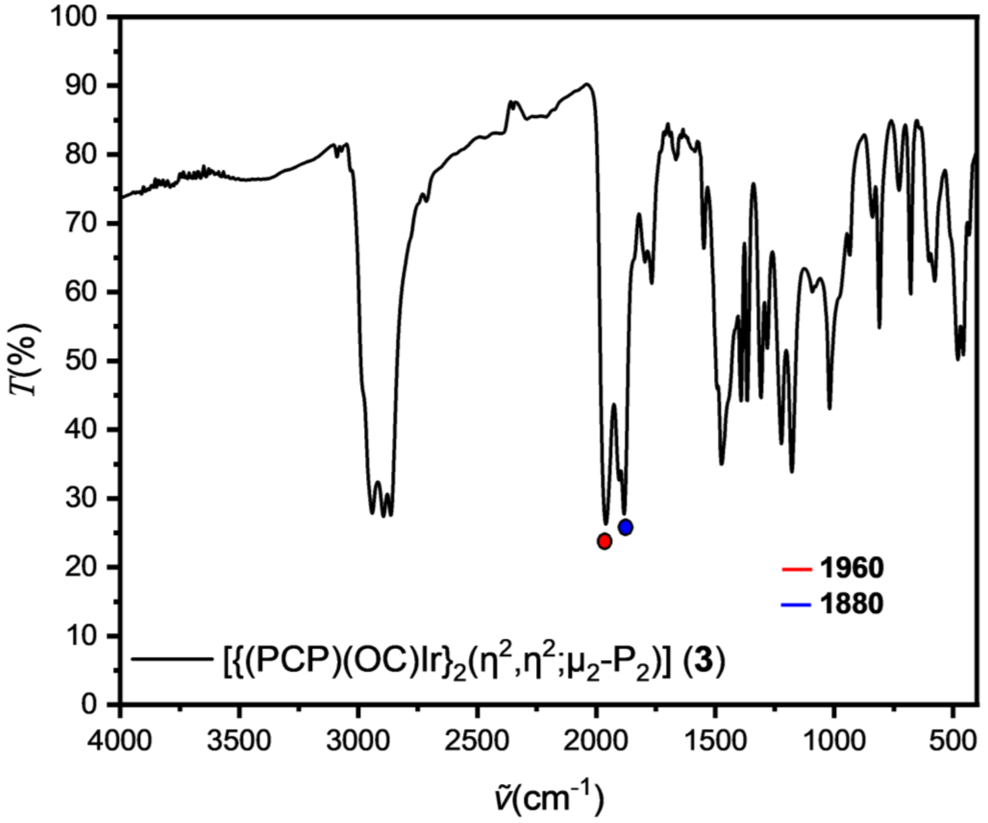

Figure S58. IR spectrum of solid [{(PCP)(OC)Ir}$_2$(η^2,η^2;μ_2-P_2)] (**3**) pressed into KBr matrix.

5.5 IR Spectral Data for [(PCP)Ir(P=CH$_2$)] (4)

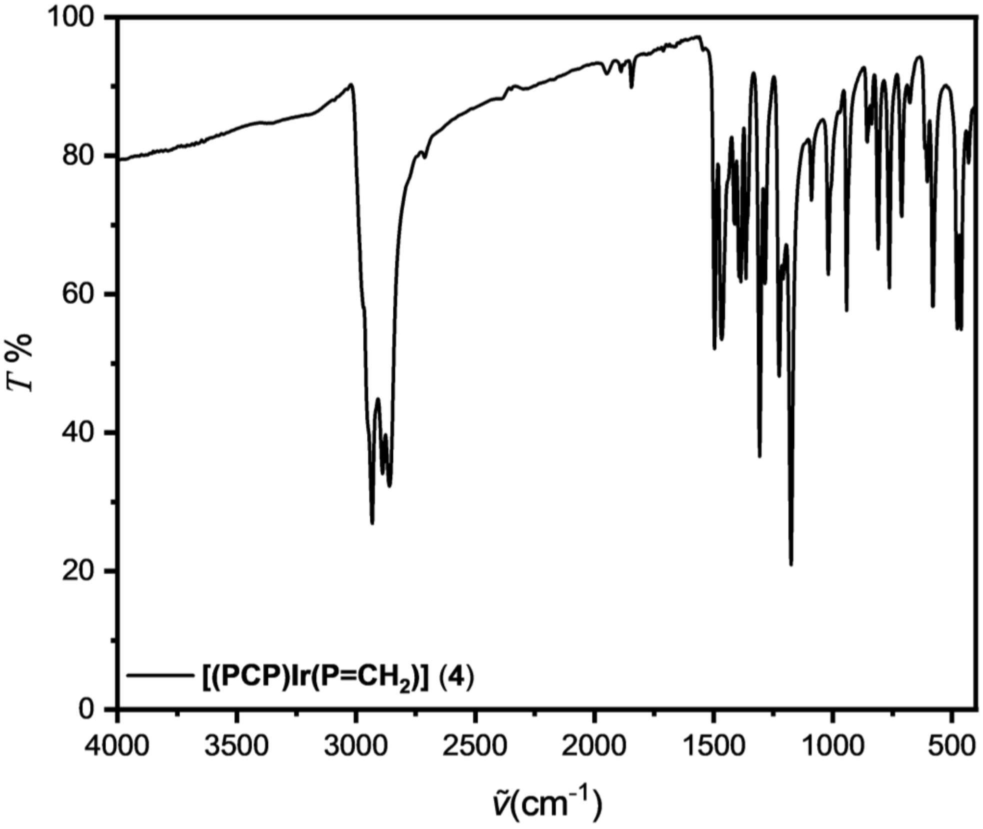

Figure S59. IR spectrum of solid [(PCP)Ir(P=CH$_2$)] (**4**) pressed into KBr matrix.

5.5.1 IR Spectral Data for [(PCP)Ir(P=CH_2)] (4) and 33% ^{13}C-enriched [(PCP)Ir(P=$^{13}CH_2$)] (4-^{13}C).

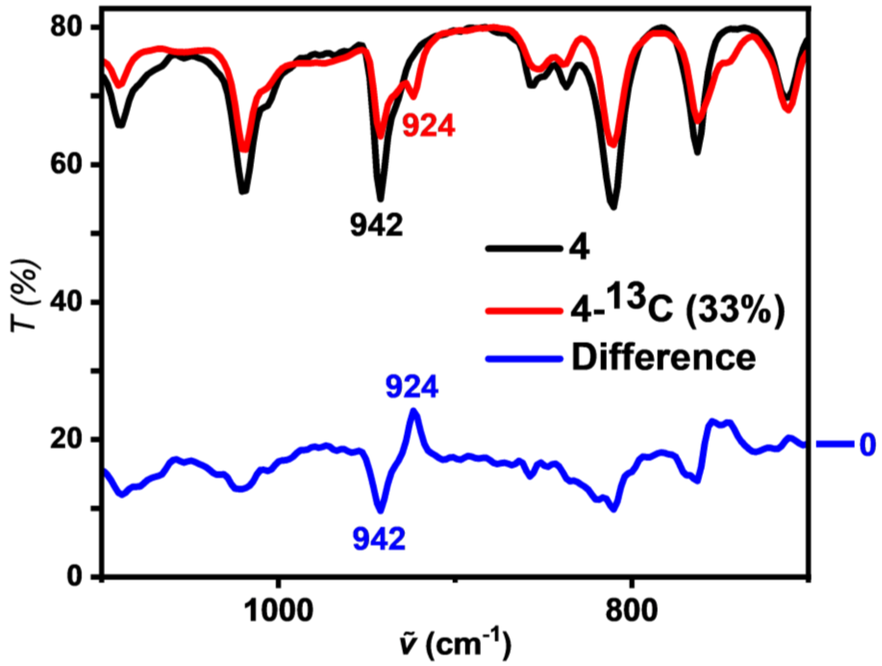

Figure S60. Comparison of IR spectra of solid [(PCP)Ir(P=CH_2)] (**4**) and 33% ^{13}C-enriched [(PCP)Ir(P=$^{13}CH_2$)] (**4-^{13}C**), pressed into KBr matrices.

5.6 IR Spectral Data for [(PCP)Ir(CO)](PCO)

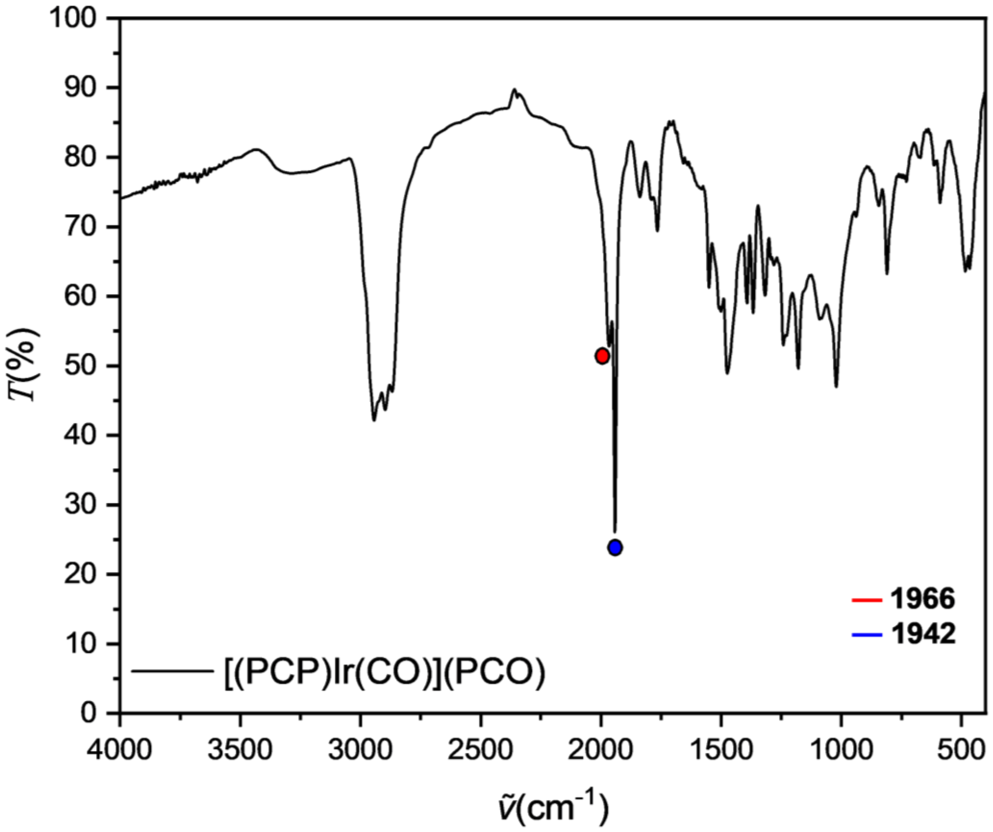

Figure S61. IR spectrum of solid [(PCP)Ir(CO)](PCO) pressed into KBr matrix.

5.7 IR Spectral Data for [(PCP)Ir{P(=CH_2)(NAd)}] (5).

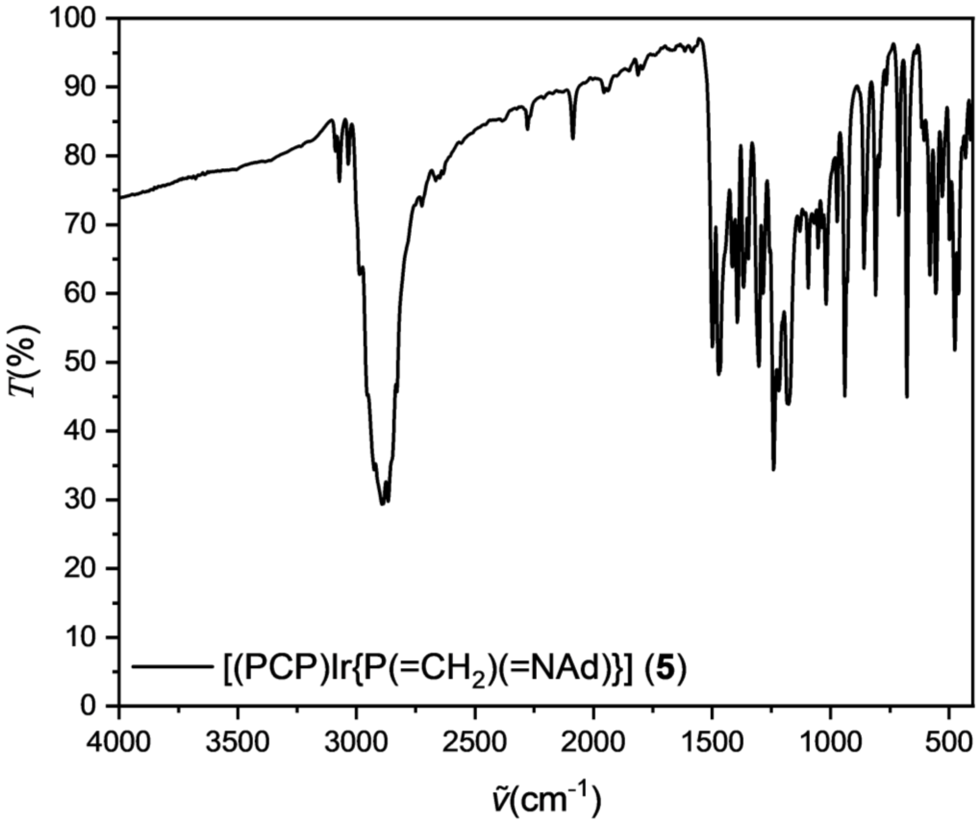

Figure S62. IR spectrum of solid [(PCP)Ir{P(=CH_2)(NAd)}] (**5**) pressed into KBr matrix.

6 UV-vis Spectroscopy

6.1 UV-vis Spectral Data for [(PCP)IrCl] (1).

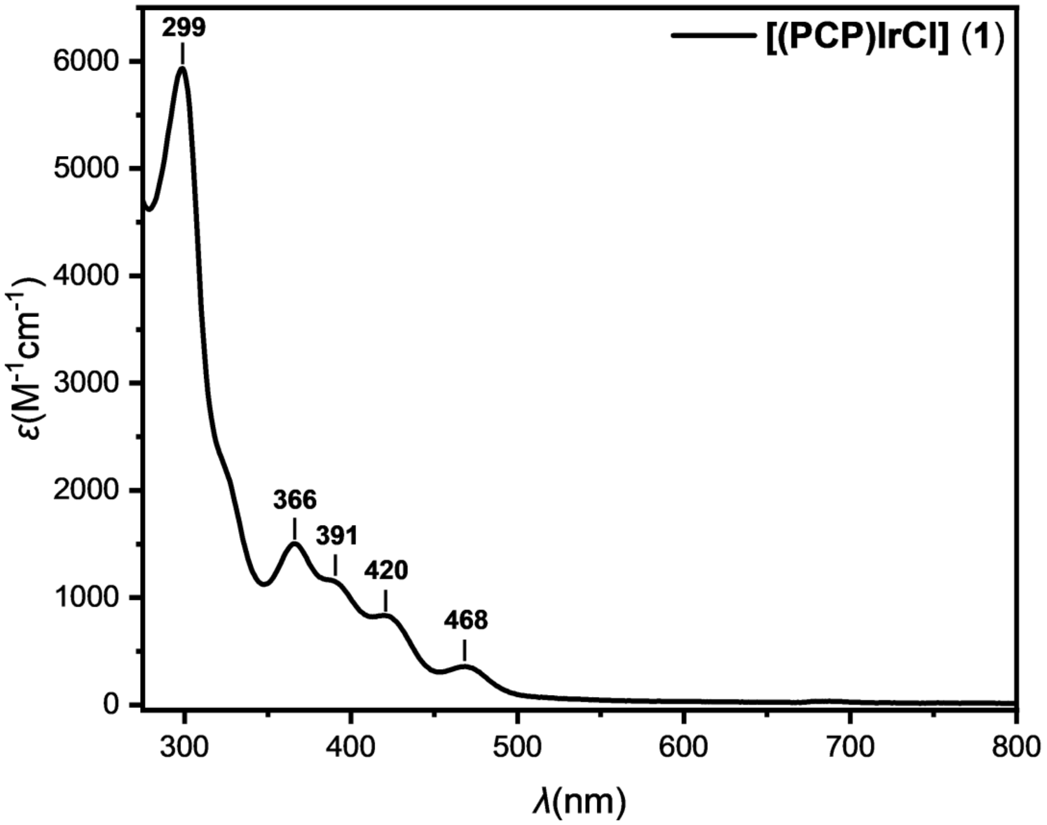

Figure S63. UV-vis spectrum of [(PCP)IrCl] (**1**) in THF. The above spectrum consists of data recorded at $2.02 \cdot 10^{-4}$ M.

6.2 UV-vis Spectral Data for [(PCP)Ir(PCO)] (2).

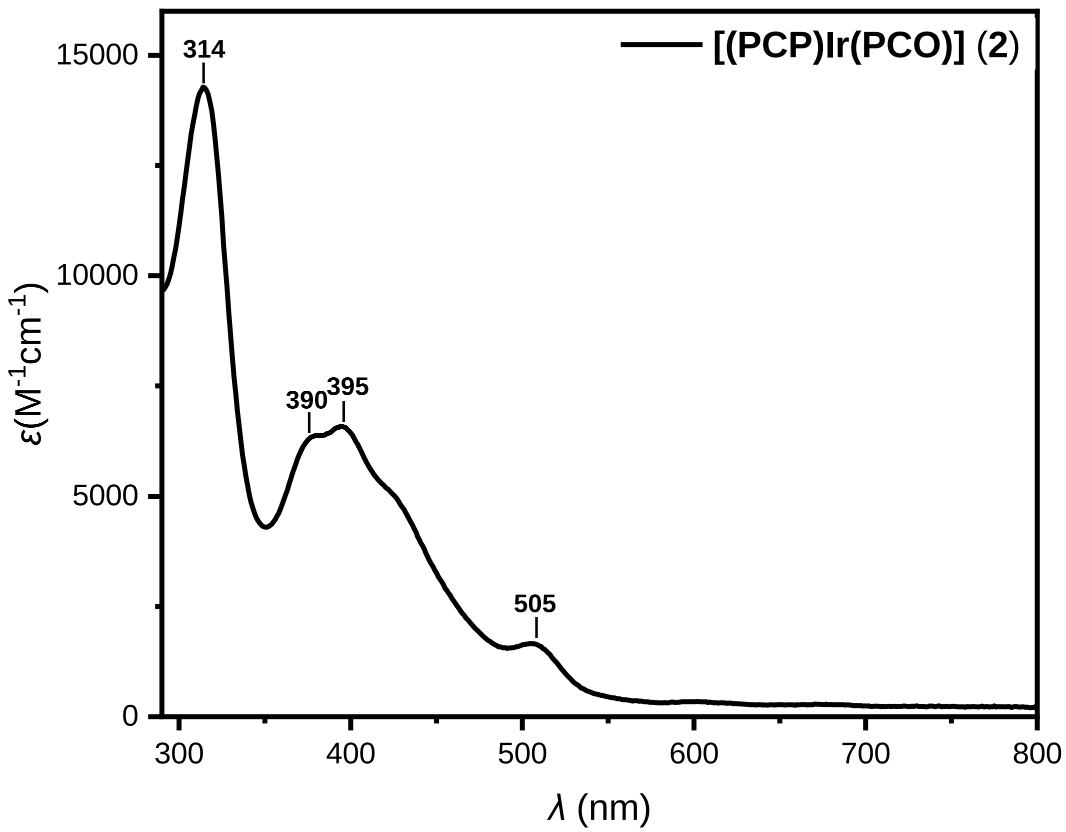

Figure S64. UV-vis spectrum of a 7.80 · 10^{-5} M solution of [(PCP)Ir(PCO)] (**2**) in THF.

6.3 UV-vis Spectral Data for [(PCP)Ir(P=CH$_2$] (4).

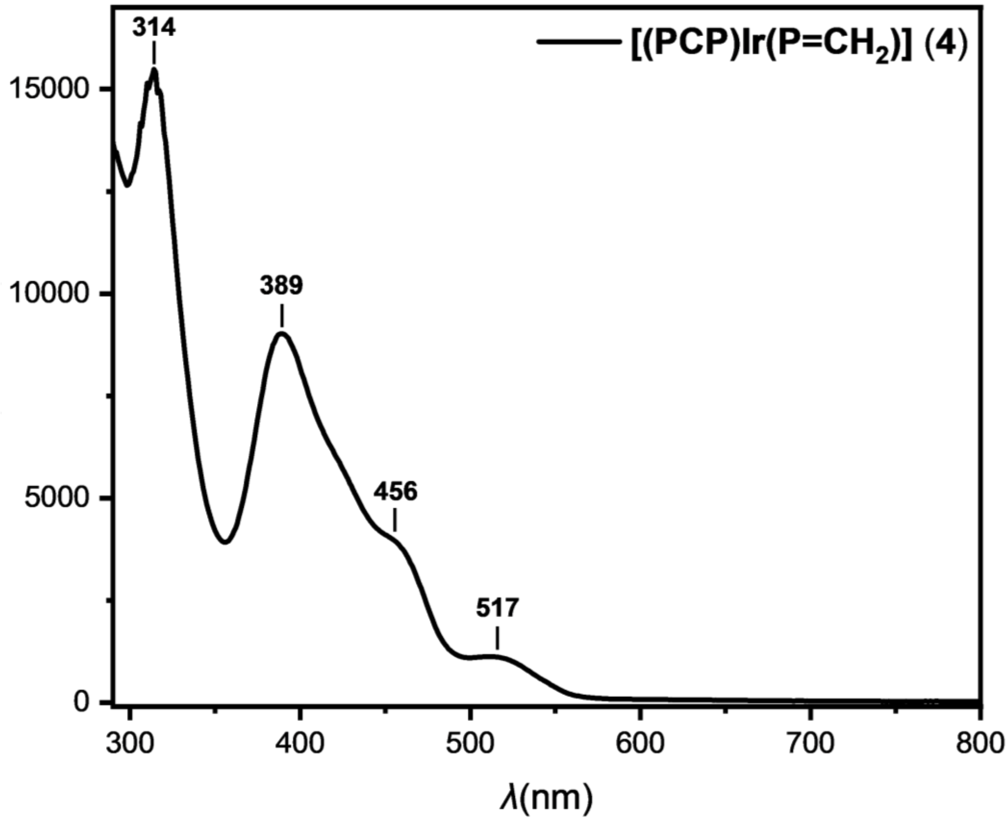

Figure S65. UV-vis spectrum of a 1.95 · 10^{-4} M solution of [(PCP)Ir(PCH$_2$)] (**4**) in THF.

6.4 UV-vis Spectral Data for [(PCP)Ir{P(=CH$_2$)(NAd)}] (5).

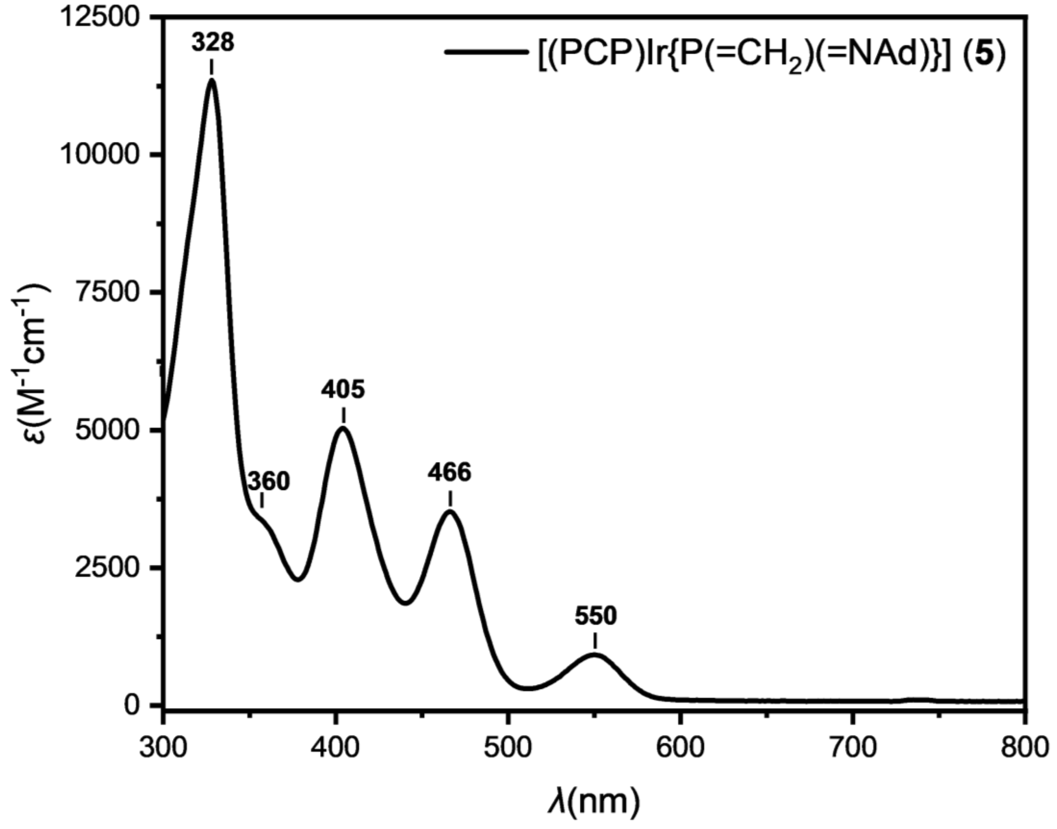

Figure S66. UV-vis spectrum of a 1.44 · 10^{-4} M solution of [(PCP)Ir{P(=CH$_2$)(NAd)}] (**5**) in THF.

7 Crystallographic Data

7.1 Crystallographic Tables

Table S1. Crystallographic data for complexes **1** and **2**.

**Complex** **CCDC entry**	[(PCP)IrCl] (**1**) 2536546	[(PCP)Ir(PCO)] (**2**) 2536547
Empirical formula	$C_{21}H_{44}ClIrN_2P_2$	$C_{22}H_{44}IrN_2OP_3$
Formula weight	614.17	637.70
Temperature / K	100(2)	100(2)
Crystal system	monoclinic	orthorhombic
Spacegroup	$P2_1/n$	*Pbca*
a / Å	22.0457(6)	16.8180(2)
b / Å	14.74730(10)	16.4207(2)
c / Å	12.2060(3)	18.8481(2)
α / °	90	90
β / °	140.468(5)	90
γ / °	90	90
V / Å^3	2525.89(19)	5205.15(10)
Z	4	8
ρ_{calc} g/cm^3	1.615	1.628
μ / mm^{-1}	5.528	5.330
F(000)	1232.0	2560.0
Crystal size / mm^3	0.45 × 0.32 × 0.29	0.607 × 0.227 × 0.17
Radiation (Å)	Mo K$_\alpha$ (λ = 0.71073)	Mo K$_\alpha$ (λ = 0.71073)
2θ range / °	6.556 to 50.122	6.952 to 49.998
Index ranges	$-26 \leq h \leq 26$, $-17 \leq k \leq 17$, $-14 \leq l \leq 14$	$-20 \leq h \leq 20$, $-19 \leq k \leq 15$, $-22 \leq l \leq 18$
Reflections collected	122613	29115
Independent reflections	4466 [R_{int} = 0.0338, R_{sigma} = 0.0084]	4560 [R_{int} = 0.0325, R_{sigma} = 0.0223]
Data / restraints / parameters	4466/0/256	4560/0/274
Goodness-of-fit on F^2	1.185	1.092
Final R indexes [$I \geq 2\sigma(I)$]	R_1 = 0.0142, wR_2 = 0.0362	R_1 = 0.0209, wR_2 = 0.0440
Final R indexes [all data]	R_1 = 0.0152, wR_2 = 0.0369	R_1 = 0.0268, wR_2 = 0.0467
Largest diff. peak/hole / e Å^{-3}	0.89/-0.38	1.04/-0.77

Table S2. Crystallographic data for complexes **3** and **4**

**Complex** **CCDC entry**	[{(PCP)(OC)Ir}$_2$(η^2,η^2;μ_2-P_2)] (**3**) 2536548	[(PCP)Ir(P=CH$_2$)] (**4**) 2536549
Empirical formula	$C_{44}H_{88}Ir_2N_4O_2P_6$	$C_{22}H_{46}IrN_2P_3$
Formula weight	1275.490	623.72
Temperature / K	100(2)	100(2)
Crystal system	triclinic	triclinic
Spacegroup	$P\bar{1}$	$P1$
a / Å	14.1953(4)	7.85450(10)
b / Å	14.2420(3)	8.44610(10)
c / Å	16.6630(5)	10.5425(2)
α / °	104.996(2)	98.9560(10)
β / °	111.447(3)	104.207(2)
γ / °	95.599(2)	103.575(2)
V / Å^3	2958.70(16)	641.954(18)
Z	2	1
ρ_{calc} g/cm^3	1.432	1.613
μ / mm^{-1}	4.702	5.398
F(000)	1278.1	314.0
Crystal size / mm^3	0.217 × 0.105 × 0.05	0.21 × 0.14 × 0.13
Radiation (Å)	Mo Kα (λ = 0.71073)	Mo Kα (λ = 0.71073)
2θ range / °	6.7 to 50.06	7.222 to 61.624
Index ranges	$-16 \leq h \leq 16$, $-16 \leq k \leq 16$, $-19 \leq l \leq 19$	$-10 \leq h \leq 10$, $-12 \leq k \leq 12$, $-15 \leq l \leq 15$
Reflections collected	67465	37702
Independent reflections	10417 [R_{int} = 0.1474, R_{sigma} = 0.1175]	7216 [R_{int} = 0.0372, R_{sigma} = 0.0428]
Data / restraints / parameters	10417/0/597	7216/3/265
Goodness-of-fit on F^2	0.982	1.001
Final R indexes [$I \geq 2\sigma(I)$]	R_1 = 0.0470, wR_2 = 0.0659	R_1 = 0.0185, wR_2 = 0.0341
Final R indexes [all data]	R_1 = 0.0905, wR_2 = 0.0775	R_1 = 0.0186, wR_2 = 0.0341
Largest diff. peak/hole / e Å^{-3}	3.79/-1.62	1.01/-0.62

Table S3. Crystallographic data for [(PCP)Ir(CO)](PCO) and $PhMe_2P$=CHCOMe.

**Complex**<br>**CCDC entry**	[(PCP)Ir(CO)](PCO)<br>2536550	$PhMe_2P$=CHCOMe<br>2536551
Empirical formula	$C_{23}H_{44}IrN_2O_2P_3$	$C_{11}H_{15}OP$
Formula weight	665.71	194.20
Temperature / K	100(2)	100(2)
Crystal system	monoclinic	tetragonal
Spacegroup	$P2_1$/c	$P\bar{4}2_1c$
a / Å	15.5661(3)	15.89610(10)
b / Å	15.0391(3)	15.89610(10)
c / Å	12.3593(2)	8.66660(10)
α / °	90	90
β / °	105.026(2)	90
γ / °	90	90
V / Å^3	2794.39(9)	2189.93(4)
Z	4	8
ρ_{calc} g/cm^3	1.582	1.178
μ / mm^{-1}	4.971	0.211
F(000)	1336.0	832.0
Crystal size / mm^3	0.234 × 0.132 × 0.081	0.38 × 0.14 × 0.12
Radiation (Å)	Mo Kα (λ = 0.71073)	Mo Kα (λ = 0.71073)
2θ range / °	7.19 to 49.994	6.956 to 50.068
Index ranges	$-18 \le h \le 18$, $-17 \le k \le 12$, $-14 \le l \le 14$	$-18 \le h \le 18$, $-18 \le k \le 18$, $-10 \le l \le 10$
Reflections collected	27637	59972
Independent reflections	4915 [R_{int} = 0.0224, R_{sigma} = 0.0163]	1936 [R_{int} = 0.0618, R_{sigma} = 0.0165]
Data / restraints / parameters	4915/0/292	1936/0/121
Goodness-of-fit on F^2	1.146	1.072
Final R indexes [$I \ge 2\sigma(I)$]	R_1 = 0.0171, wR_2 = 0.0375	R_1 = 0.0246, wR_2 = 0.0595
Final R indexes [all data]	R_1 = 0.0219, wR_2 = 0.0393	R_1 = 0.0266, wR_2 = 0.0604
Largest diff. peak/hole / e Å^{-3}	0.87/-0.45	1.43 / –0.93

Table S4. Crystallographic data for complex **5**.

**Complex**<br>**CCDC entry**	[(PCP)Ir{P(=CH_2)(NAd)}] (**5**)<br>2536552
Empirical formula	$C_{39}H_{69}IrN_3P_3$
Formula weight	865.08
Temperature / K	100(2)
Crystal system	monoclinic
Spacegroup	$P2_1/n$
a / Å	12.65970(10)
b / Å	20.1860(2)
c / Å	15.84520(10)
α / °	90
β / °	92.0110(10)
γ / °	90
V / Å^3	4046.73(6)
Z	4
ρ_{calc} g/cm^3	1.420
μ / mm^{-1}	3.448
F(000)	1784.0
Crystal size / mm^3	0.23 × 0.14 × 0.11
Radiation (Å)	Mo Kα (λ = 0.71073)
2θ range / °	6.54 to 50.114
Index ranges	$-15 \le h \le 15$, $-24 \le k \le 24$, $-18 \le l \le 18$
Reflections collected	143611
Independent reflections	7157 [R_{int} = 0.0776, R_{sigma} = 0.0261]
Data / restraints / parameters	7157/45/510
Goodness-of-fit on F^2	1.260
Final R indexes [$I \ge 2\sigma(I)$]	R_1 = 0.0316, wR_2 = 0.0561
Final R indexes [all data]	R_1 = 0.0365, wR_2 = 0.0572
Largest diff. peak/hole / e Å^{-3}	0.66/-0.78

7.2 Thermal Ellipsoid Plot of [(PCP)IrCl] (1)

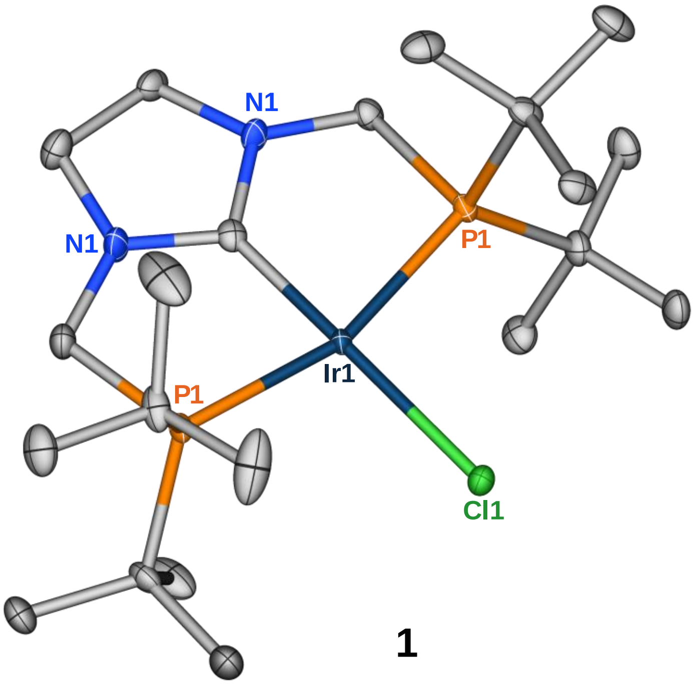

1

Figure S67. ORTEP plot of [(PCP)IrCl] (**1**), recorded at 100(2) K. The thermal ellipsoids are set to 50% probability and the H-atoms are omitted.

7.3 Thermal Ellipsoid Plot of [(PCP)Ir(PCO)] (2)

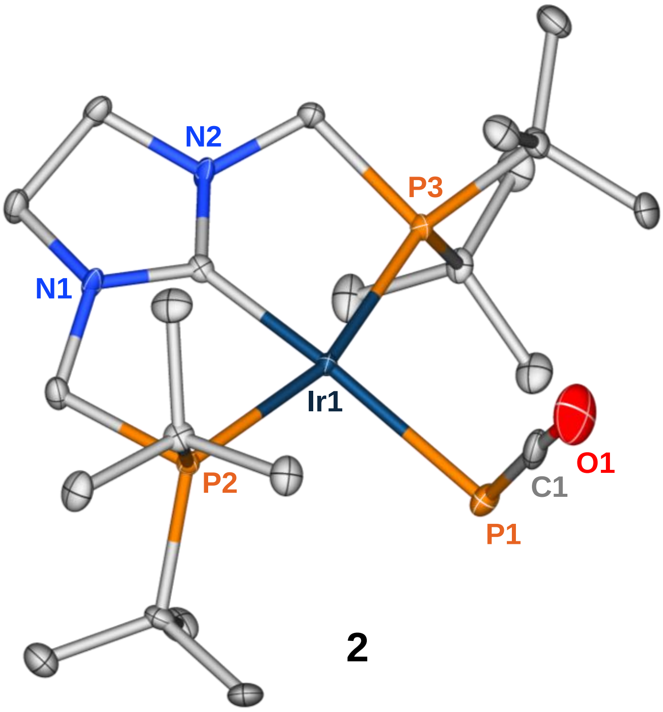

2

Figure S68. ORTEP plot of [(PCP)Ir(PCO)] (**2**), recorded at 100(2) K. The thermal ellipsoids are set to 50% probability and the H-atoms are omitted.

7.4 Thermal Ellipsoid Plot of [{(PCP)(OC)Ir}$_2$(η^2,η^2;μ_2-P_2)] (3)

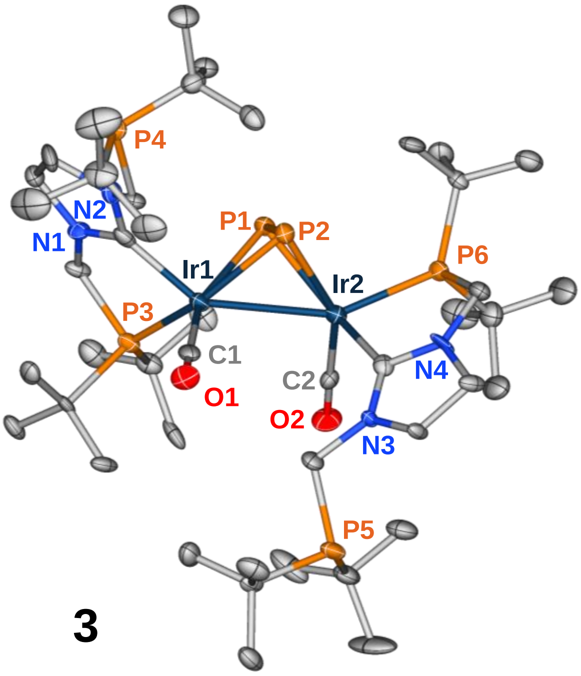

Figure S69. ORTEP plot of [{(PCP)(OC)Ir}$_2$(η^2,η^2;μ_2-P_2)] (**3**), recorded at 100(2) K. The thermal ellipsoids are set to 50% probability and the H-atoms are omitted.

7.5 Thermal Ellipsoid Plot of [(PCP)Ir(P=CH_2)] (4)

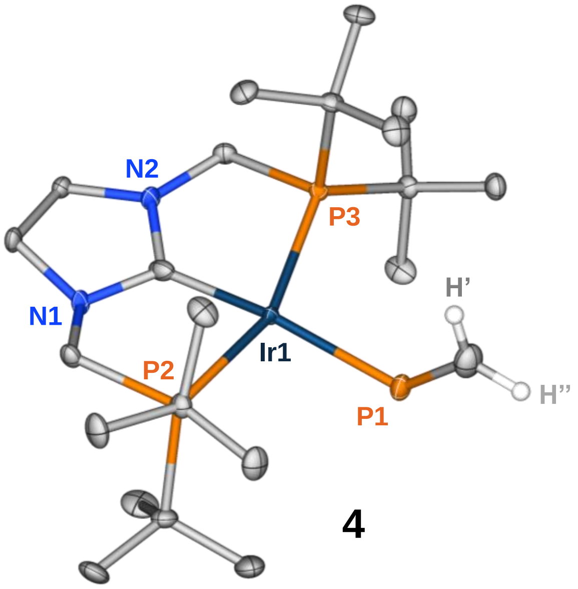

Figure S70. ORTEP plot of [(PCP)Ir(P=CH_2)] (**4**), recorded at 100(2) K. The thermal ellipsoids are set to 50% probability, and the H-atoms are omitted, except on the P=CH_2^- group.

7.6 Thermal Ellipsoid Plot of [(PCP)Ir(CO)](PCO)

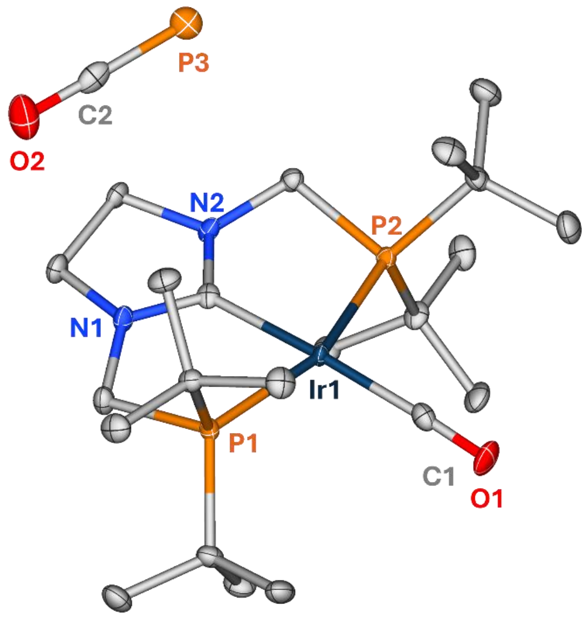

Figure S71. ORTEP plot of [(PCP)Ir(CO)](PCO), recorded at 100(2) K. The thermal ellipsoids are set to 50% probability, and the H atoms are omitted.

7.7 Thermal Ellipsoid Plot of $PhMe_2P=CHCOMe$

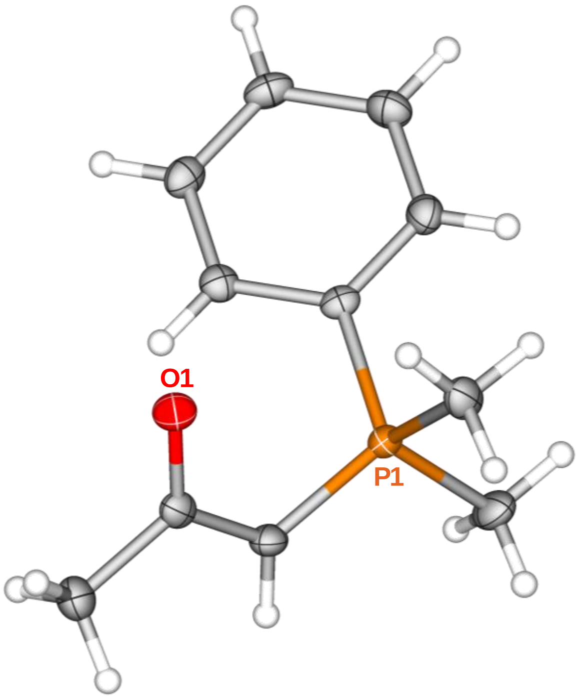

Figure S72. ORTEP plot of $PhMe_2P=CHCOMe$, recorded at 100(2) K. The thermal ellipsoids are set to 50% probability.

7.8 Thermal Ellipsoid Plot of [(PCP)Ir{P(=CH_2)(NAd)}] (5)

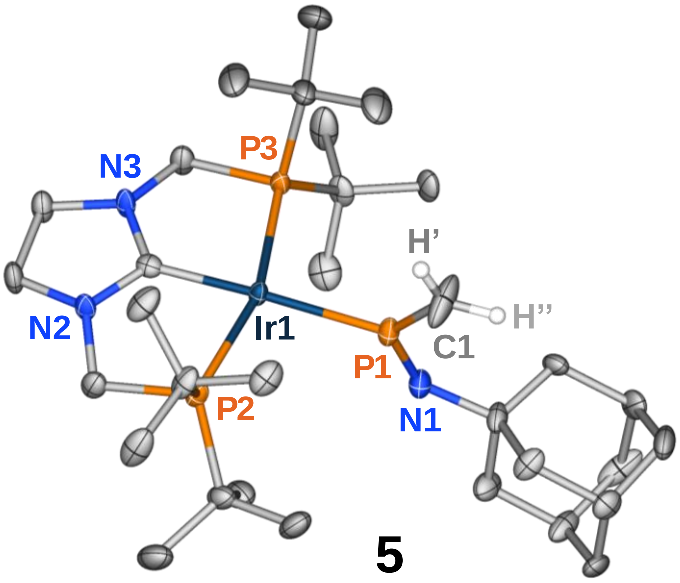

5

Figure S73. ORTEP plot of [(PCP)Ir{P(=CH_2)(NAd)}] (**5**) recorded at 100(2) K. The thermal ellipsoids are set to 50% probability and the H-atoms are omitted, except on the P=CH_2^- group. Rotational disorder of the adamantyl group is omitted for clarity.

8 Kinetic Studies by NMR Spectroscopy

For the temperature-dependent kinetic experiments, monitoring the interchange between the two protons of the $[P{=}CH_2]^-$ group in **4**, a toluene-d_8 solution was prepared and transferred to a J. Young NMR tube. The tube was inserted into the NMR spectrometer, heated to the desired temperature, and allowed to thermally equilibrate for 10-15 minutes before the beginning of each measurement. The actual temperature of the sample was calculated by monitoring an external reference sample of ethylene glycol and constructing a linear correlation (integrated in Bruker software) to correct the temperature at each power setting of the NMR probe. Before each kinetic measurement, automatic shimming with convection compensation was carried out to improve data quality.

In the low-temperature regime of the experiments (–5 °C to +25 °C), rate constants for interchange between the two protons of the $[P{=}CH_2]^-$ group were estimated through NOESY build-up using the Peak Amplitude Normalization for Improved Cross-relaxation (PANIC) approach.[11] In the high-temperature regime of the experiments (+40 °C to +105 °C), rate constants were estimated through line-width analysis of the 1D ^{1}H NMR spectra using Topspin's DNMR module.

Table S5. Exchange rate data for interchange of $[P{=}CH_2]^-$ protons in **4**.

T **(°C)**	$\boldsymbol{k_{ex}}$ **(Hz)**
–5	0.7
15	4.7
25	15.4
41.4	44.0
47	113.2
52.3	158
57.7	211
63	373
68.4	548
73.3	766
79.1	1132
84.4	2267
89.8	2571
95.1	3071
100.5	4144
105.8	7191

9 Computational Studies

9.1. Computational methodology

We performed density functional theory calculations using the ORCA program v.6.0.0.[12-14] Geometry optimizations were performed on non-truncated models of the complexes using the hybrid PBE0[15] functional and the def2-TZVP(-f) basis set[16, 17] combined with the auxiliary basis set def2-J.[16] The convergence criteria for the calculations were set up to TightSCF, and the convergence strategy to SlowConv. The RI-J approximation for the Coulomb integrals and the COSX numerical chain-of-sphere integration for the HF exchange integrals (RIJCOSX) were employed to accelerate DFT calculations.[18] The SHARK integral generation and digestion engine was employed to accelerate numerical integration.[19] Grimme's D3 method, combined with the Becke-Johnson (D3BJ) damping scheme, was employed to account for dispersion effects.[20, 21]

Analytical frequency calculations at the same level of theory as the optimizations (PBE0-D3/def2-TZVP(-f)/defgrid2) were performed to confirm that the equilibrium structures were minima on the potential energy surface. The latter calculations were also used to obtain the zero-point energy (ZPE), thermal, and entropic corrections at 298.15 K ($\Delta G^{0\rightarrow 298\ K}$) for each species, assuming ideal-gas behavior and the rigid-rotor-harmonic-oscillator approximations. The wavefunctions and gas-phase energies for all species were refined through subsequent single-point energy calculations on the optimized structures. For single-point calculations, we employed the meta-GGA hybrid TPSSh functional, in combination with the def2-TZVP basis set.[22-24] The solution-state energy (toluene) of the structures was calculated as single-point energies using the universal solvation model based on solute electron density (SMD) at the TPSSh-D3/def2-TZVP level of theory.[25] The GEPOL algorithm[26-28] was used to generate the solvent cavity as a solvent-excluded surface. The electronic structures of the optimized equilibrium species were scrutinized, including quasi-restricted orbitals[29, 30] and Mayer bond orders at the TPSSh-D3/def2-TZVP level of theory. Wiberg bond orders and QTAIM analyses were performed using Multiwfn 3.8.[31]

Transition states were located via relaxed potential-energy surface scans along the relevant reaction coordinates, followed by transition-state optimizations in ORCA 6.0.0. Each transition state was validated by the presence of a single imaginary vibrational frequency corresponding to motion along the reaction coordinate. Electronic energies were subsequently refined at the TPSSh-D3/def2-TZVP level in the gas phase and with implicit solvation using the SMD model. Nudged elastic band (NEB) calculations[32] were additionally performed and yielded reaction pathways consistent with the optimized transition states and associated energy barriers.

^{31}P NMR shielding tensors were calculated in ORCA using the TPSSh functional with the ZORA scalar relativistic approximation. ZORA-def2-TZVP basis sets were used together with a SARC-ZORA-TZVP basis set for Ir. Gauge-including atomic orbitals (GIAOs) were employed for the shielding calculations. The diamagnetic (δ^{DSO}), paramagnetic (δ^{PSO}), and total calculated ^{31}P chemical-shift contributions were obtained by referencing the corresponding isotropic shielding contributions of [(PCP)Ir(PCH$_2$)] to those calculated for H_3PO_4 at the same level of theory, according to the equation $\delta = \sigma_{ref} - \sigma_{sample}$.

9.2 Computational data

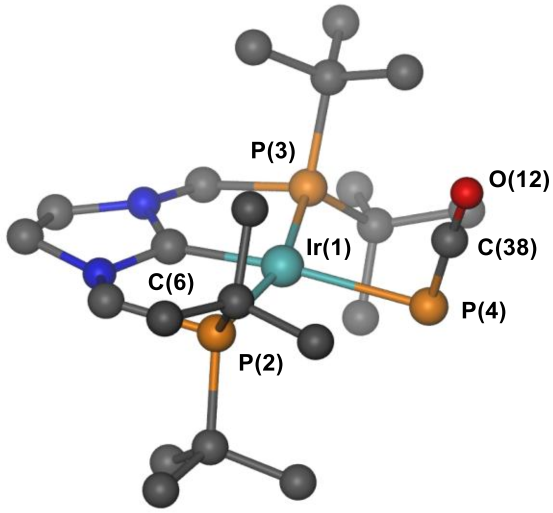

Table S6. Calculated distances (Å) and angles (◦) of [(PCP)Ir(PCO)] (**2**). The schematic picture corresponds to the optimized singlet (*S* = 0) equilibrium structure. Hydrogen atoms are omitted for clarity. Geometries were optimized at the PBE0-D3/def2-TZVP(-f) level of theory

Bond	XRD	Singlet (*S* = 0)		Triplet (*S* = 1)	
		Calculated	Δ	Calculated	Δ
Ir(1)–P(2)	2.284	2.284	0.000	2.310	0.026
Ir(1)–P(3)	2.282	2.281	0.001	2.304	0.023
Ir(1)–P(4)	2.481	2.485	0.004	2.414	0.067
Ir(1)–C(6)	1.932	1.923	0.009	1.970	0.037
P(4)–C(38)	1.621	1.637	0.017	1.805	0.185
C(38)–O(12)	1.185	1.173	0.012	1.194	0.009
P(2)–Ir(1)–P(3)	162	163	0	156	6
P(2)–Ir(1)–P(4)	98	100	2	101	2
P(2)–Ir(1)–C(6)	81	81	0	80	2
P(3)–Ir(1)–P(4)	99	97	2	99	0
P(3)–Ir(1)–C(6)	81	81	0	80	1
P(4)–Ir(1)–C(6)	173	172	1	178	5
Ir(1)–P(4)–C(38)	100	98	1	56	44
P(4)–C(38)–O(12)	178	178	0	144	34

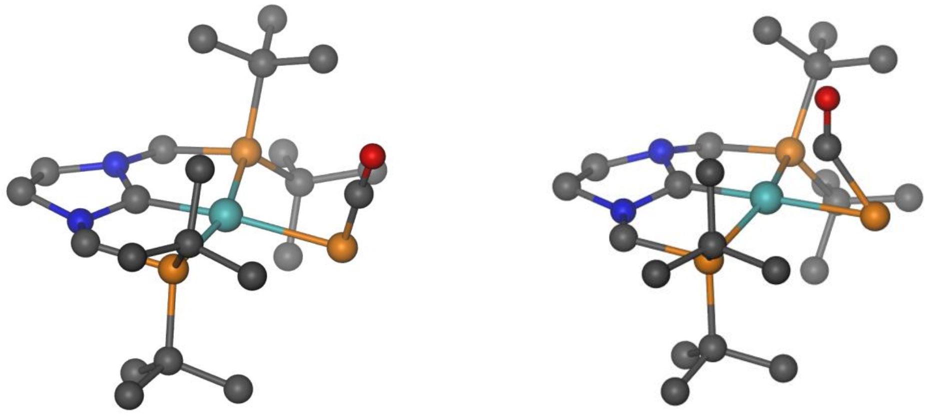

Figure S74. Calculated equilibrium geometries for singlet (S = 0) and triplet (S = 1) states of [(PCP)Ir(PCO)] (**2**) together with their gas phase relative energies calculated at the TPSSh-D3/def2-TZVP level of theory.

Table S7. Calculated Mayer and Wiberg bond orders of [(PCP)Ir(PCO)] (**2**) (S = 0) at the TPSSh-D3/def2-TZVP level of theory

Bond	MBO	WBO
Ir(1)–P(2)	0.931	1.069
Ir(1)–P(3)	0.925	1.075
Ir(1)–P(4)	0.715	0.902
Ir(1)–C(6)	1.217	1.395
P(4)–C(38)	1.883	2.395
C(38)–O(12)	2.008	2.820

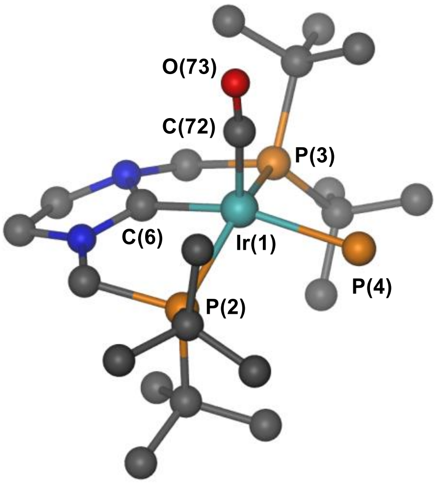

Table S8. Calculated distances (Å) and angles (◦) of [(PCP)Ir(P)(CO)] (**A**). The schematic picture corresponds to the optimized triplet (S = 1) equilibrium structure. Hydrogen atoms are omitted for clarity. Geometries were optimized at the PBE0-D3/def2-TZVP(-f) level of theory

Bond	Singlet	Triplet
	Calculated	Calculated
Ir(1)–P(2)	2.346	2.320
Ir(1)–P(3)	2.396	2.317
Ir(1)–P(4)	2.269	2.387
Ir(1)–C(6)	1.974	2.000
Ir(1)–C(72)	1.868	1.893
C(72)–O(73)	1.154	1.152
P(2)–Ir(1)–P(4)	59	94
P(2)–Ir(1)–P(3)	146	141
P(2)–Ir(1)–C(6)	87	79
P(2)–Ir(1)–C(72)	105	108
P(3)–Ir(1)–P(4)	109	96
P(3)–Ir(1)–C(6)	80	80
P(3)–Ir(1)–C(72)	107	109
P(4)–Ir(1)–C(6)	133	161
P(4)–Ir(1)–C(72)	123	93
C(6)–Ir(1)–C(72)	95	106
Ir(1)–C(72)–O(73)	175	176

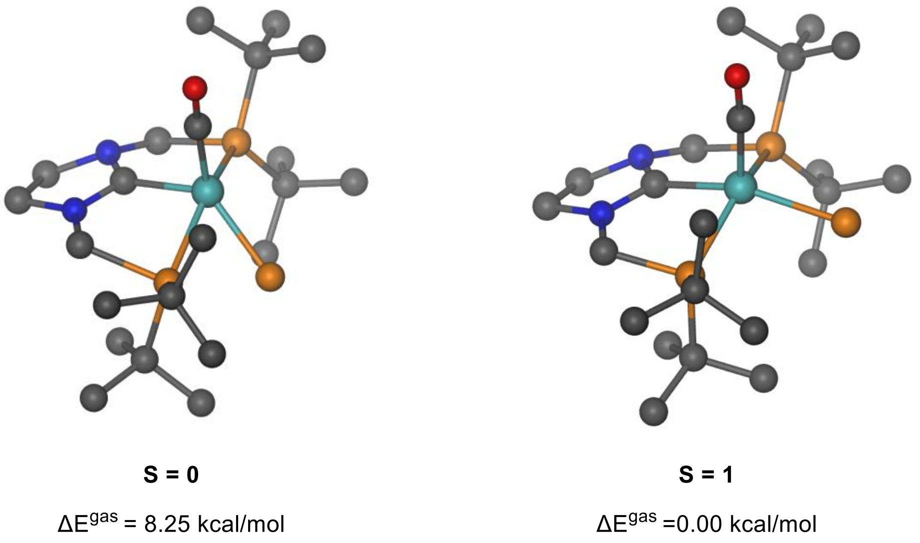

Figure S75. Calculated equilibrium geometries for singlet (S = 0) and triplet (S = 1) states of [(PCP)Ir(P)(CO)] (**A**) together with their gas phase relative energies calculated at the TPSSh-D3/def2-TZVP level of theory.

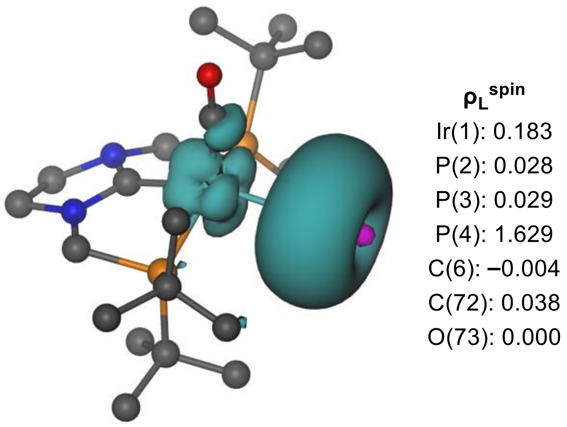

Figure S76. Löwdin spin populations for selected atoms and spin density distribution of the optimized structure of [(PCP)Ir(P)(CO)] (**A**) (S = 1) calculated at the TPSSh-D3/def2-TZVP level of theory. Isovalue is set to ± 0.003 a.u.

Table S9. Calculated Mayer and Wiberg bond orders of [(PCP)Ir(P)(CO)] (**A**) (S = 1) at the TPSSh-D3/def2-TZVP level of theory

Bond	MBO	WBO
Ir(1)–P(2)	0.969	0.979
Ir(1)–P(3)	0.931	0.995
Ir(1)–P(4)	1.049	1.344
Ir(1)–C(6)	0.805	1.072
Ir(1)–C(72)	1.264	1.613
C(72)–O(73)	2.120	3.004

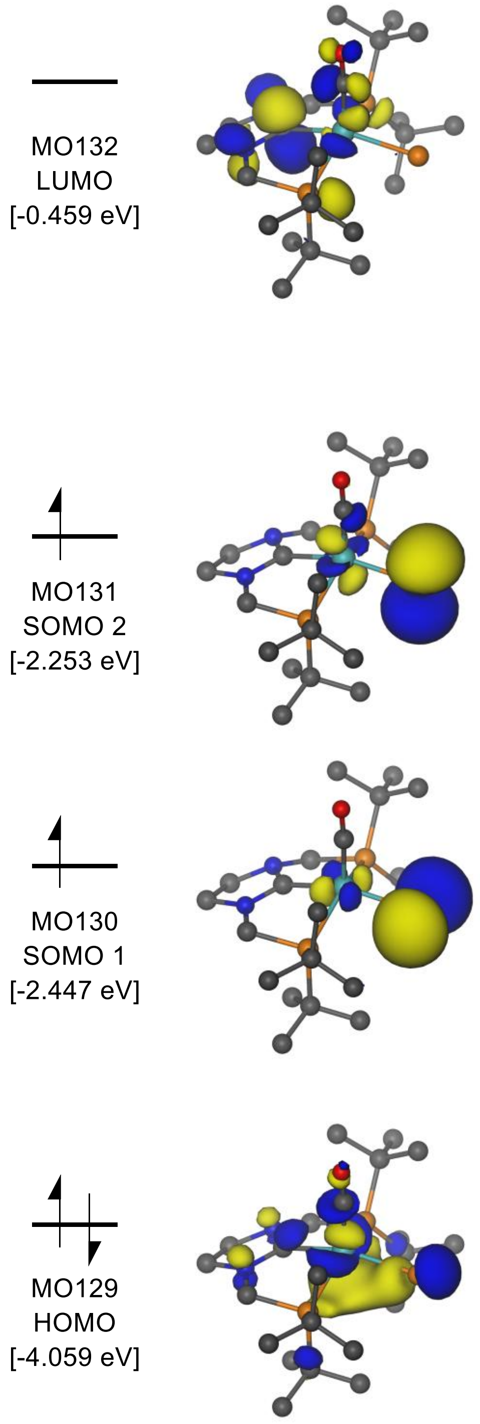

Figure S77. Frontier quasi-restricted orbitals (QROs) of [(PCP)Ir(P)(CO)] (**A**) (*S* = 1) calculated at the TPSSh-D3/def2-TZVP level of theory. Isovalue is set to ± 0.05 a.u.

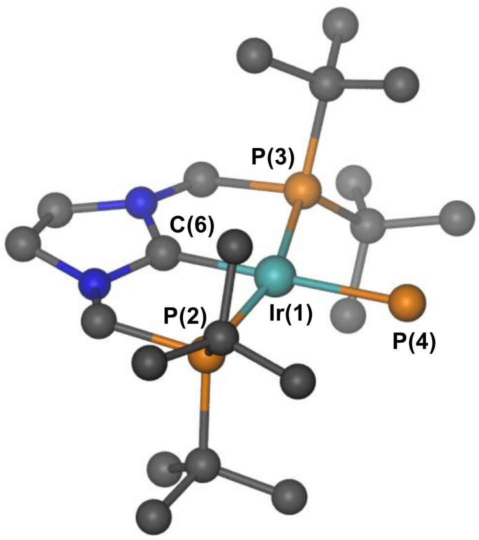

Table S10. Calculated distances (Å) and angles (◦) of [(PCP)Ir(P)] (**B**). The schematic picture corresponds to the optimized triplet (S = 1) equilibrium structure. Hydrogen atoms are omitted for clarity. Geometries were optimized at the PBE0-D3/def2-TZVP(-f) level of theory

**Bond**	**Singlet**	**Triplet**
	**Calculated**	**Calculated**
Ir(1)–P(2)	2.276	2.275
Ir(1)–P(3)	2.275	2.273
Ir(1)–P(4)	2.177	2.329
Ir(1)–C(6)	1.989	1.971
P(2)–Ir(1)–P(3)	159	162
P(2)–Ir(1)–P(4)	101	99
P(2)–Ir(1)–C(6)	80	81
P(3)–Ir(1)–P(4)	101	99
P(3)–Ir(1)–C(6)	80	81
P(4)–Ir(1)–C(6)	178	180

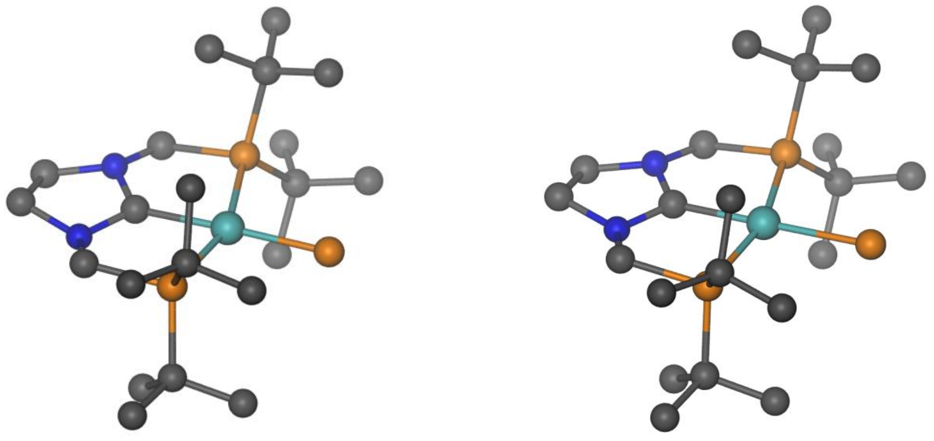

Figure S78. Calculated equilibrium geometries for singlet (*S* = 0) and triplet (*S* = 1) states of [(PCP)Ir(P)] (**B**) together with their gas phase relative energies calculated at the TPSSh-D3/def2-TZVP level of theory.

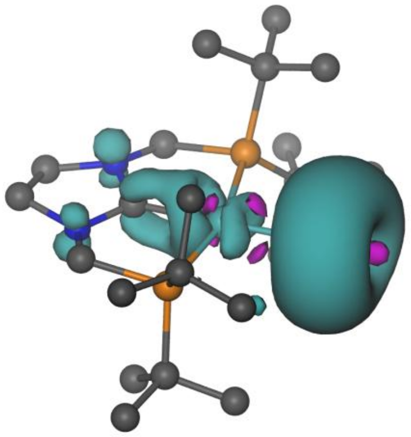

Figure S79. Löwdin spin populations for selected atoms and spin density distribution of the optimized structure of [(PCP)Ir(P)] (**B**) (*S* = 1) calculated at the TPSSh-D3/def2-TZVP level of theory. Isovalue is set to ± 0.003 a.u.

Table S11. Calculated Mayer and Wiberg bond orders of [(PCP)Ir(P)] (**B**) (*S* = 0 and *S* =1) at the TPSSh-D3/def2-TZVP level of theory

**Bond**	***S* = 0**		***S* = 1**	
	**MBO**	**WBO**	**MBO**	**WBO**
Ir(1)–P(2)	0.915	1.048	0.936	1.073
Ir(1)–P(3)	0.912	1.048	0.941	1.076
Ir(1)–P(4)	1.553	2.134	1.084	1.527
Ir(1)–C(6)	0.944	1.183	0.959	1.183

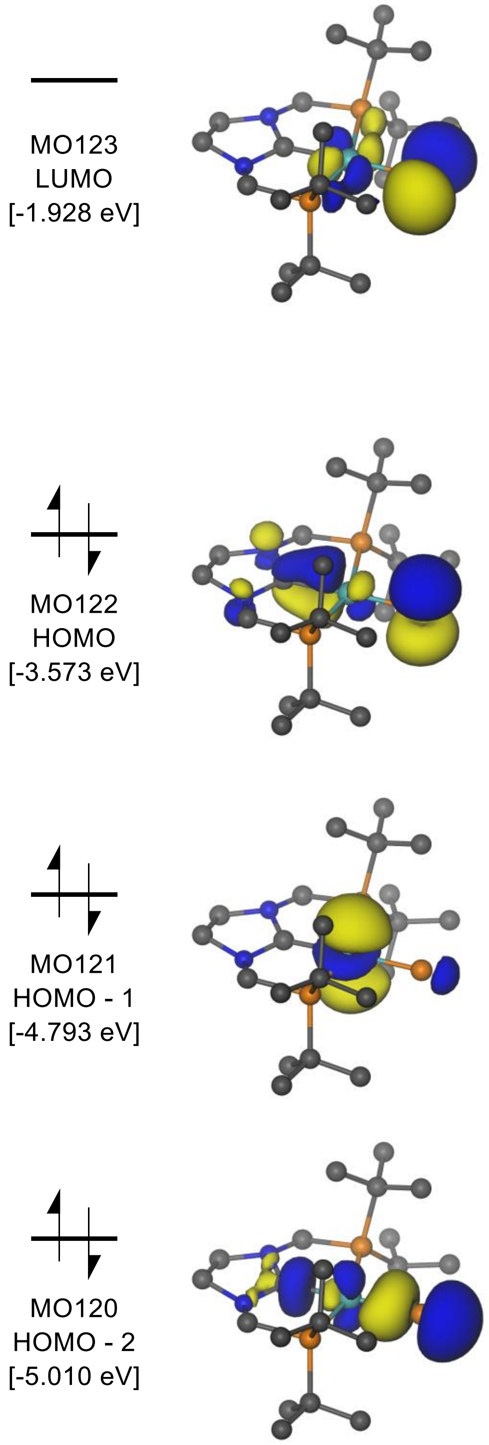

Figure S80. Frontier molecular orbitals (MOs) of [(PCP)Ir(P)] (**B**) (S = 0) calculated at the TPSSh-D3/def2-TZVP level of theory. Isovalue is set to ± 0.05 a.u.

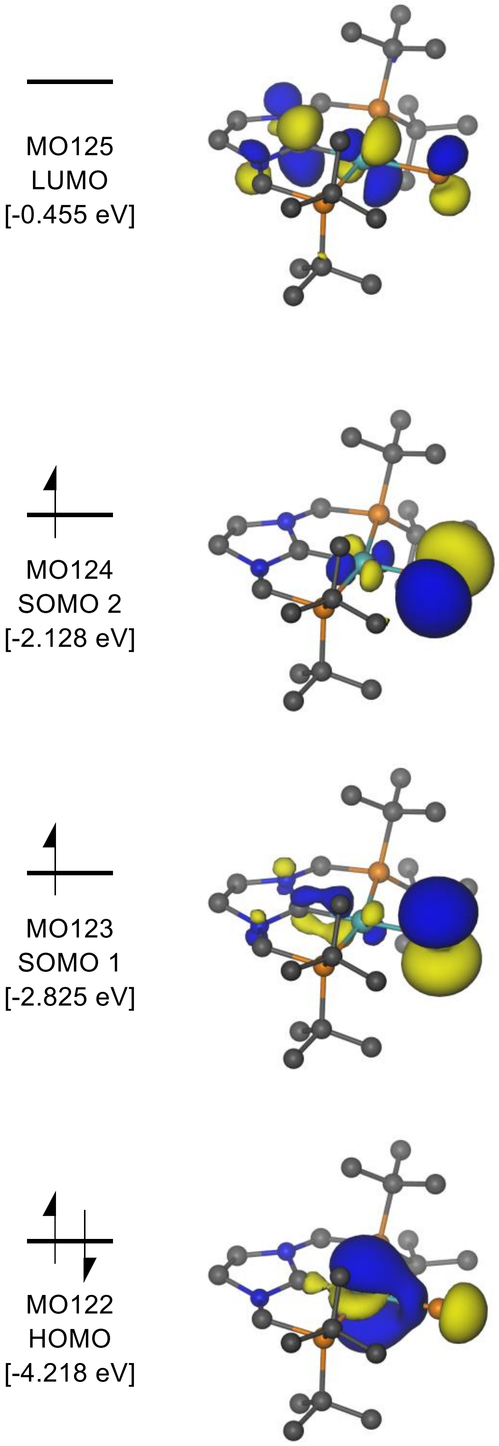

Figure S81. Frontier quasi-restricted orbitals (QROs) of [(PCP)Ir(P)] (**B**) (*S* = 1) calculated at the TPSSh-D3/def2-TZVP level of theory. Isovalue is set to ± 0.05 a.u.

Table S12. Calculated relative energies of [(PCP)Ir(P)(CO)] (**A**) and [(PCP)Ir(P)] (**B**) + CO in both singlet state (S = 0) and triplet state (S = 1).

Species	ΔG^{gas}-TPSSh	ΔG^{TOL}-TPSSh
**[(PCP)Ir(P)(CO)] (*S* = 0)**	**8.99**	**10.35**
**[(PCP)Ir(P)(CO)] (*S* = 1)**	**0.00**	**0.00**
**[(PCP)Ir(P)] (*S* = 0) + CO**	**12.19**	**14.79**
**[(PCP)Ir(P)] (*S* = 1) + CO**	**9.33**	**12.02**

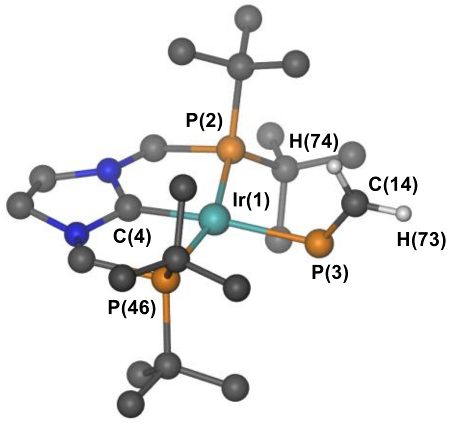

Ellipticity of electron density for P(3)–C(14) bond in [(PCP)Ir(P=CH_2)] (**4**) is 0.3159.

Table S13. Calculated distances (Å) and angles (◦) of [(PCP)Ir(P=CH_2)] (**4**). The schematic picture corresponds to the optimized singlet (S = 0) equilibrium structure. Hydrogen atoms are omitted for clarity. Geometries were optimized at the PBE0-D3/def2-TZVP(-f) level of theory

Bond	XRD	Singlet		Triplet	
		Calculated	Δ	Calculated	Δ
Ir(1)–P(2)	2.271	2.274	0.003	2.276	0.005
Ir(1)–P(3)	2.333	2.358	0.025	2.413	0.081
Ir(1)–C(4)	1.958	1.958	0.000	1.944	0.014
Ir(1)–P(46)	2.274	2.272	0.002	2.276	0.002
P(3)–C(14)	1.663	1.669	0.007	1.754	0.091
C(14)–H(74)	0.964	1.088	0.124	1.086	0.122
C(14)–H(73)	0.966	1.087	0.121	1.087	0.120
P(2)–Ir(1)–P(3)	100	99	2	99	1
P(2)–Ir(1)–C(4)	80	81	0	81	0
P(2)–Ir(1)–P(46)	158	161	4	162	4
P(3)–Ir(1)–C(4)	166	172	6	176	10
P(3)–Ir(1)–P(46)	101	100	1	99	2
C(4)–Ir(1)–P(46)	81	81	0	81	0
Ir(1)–P(3)–C(14)	122	118	3	109	13
P(3)–C(14)–H(74)	129	126	3	121	7
P(3)–C(14)–H(73)	117	120	2	122	5
H(74)–C(14)–H(73)	114	115	1	115	1

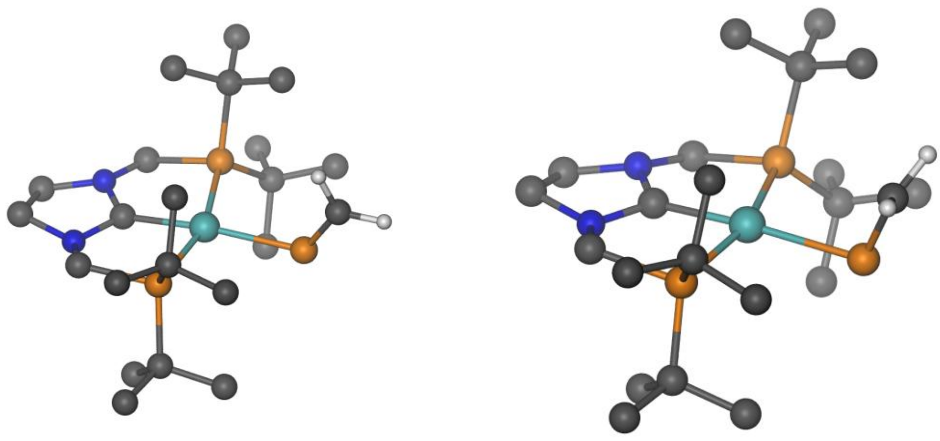

S = 0

ΔE^{gas} = 0.00 kcal/mol

S = 1

ΔE^{gas} = 31.72 kcal/mol

Figure S82. Calculated equilibrium geometries for singlet (*S* = 0) and triplet (*S* = 1) states of [(PCP)Ir(P=CH_2)] (**4**) together with their gas phase relative energies calculated at the TPSSh-D3/def2-TZVP level of theory.

Table S14. Calculated Mayer and Wiberg bond orders of [(PCP)Ir(P=CH_2)] (**4**) (*S* = 0) at the TPSSh-D3/def2-TZVP level of theory

Bond	MBO	WBO
Ir(1)–P(2)	0.921	1.081
Ir(1)–P(3)	0.924	1.228
Ir(1)–C(4)	1.101	1.273
Ir(1)–P(46)	0.943	1.086
P(3)–C(14)	1.828	2.264
C(14)–H(74)	0.930	0.933
C(14)–H(73)	0.943	0.935

Table S15. Mulliken orbital composition analysis for HOMO, LUMO, and LUMO+1 of [(PCP)Ir(PCH_2)] (**4**) (*S* = 0, displayed in the figure below) calculated at the TPSSh-D3/def2-TZVP level of theory.

**HOMO**		**LUMO**		**LUMO + 1**	
**Atom**	**Composition (%)**	**Atom**	**Composition (%)**	**Atom**	**Composition (%)**
Ir(1)	22.70	Ir(1)	28.77	Ir(1)	4.27
P(2)	0.50	P(2)	12.31	P(2)	0.00
P(3)	62.50	P(3)	7.69	P(3)	51.32
P(46)	0.31	P(46)	12.24	P(46)	1.19
C(4)	4.17	C(4)	23.04	C(4)	1.18
C(14)	1.92	C(14)	0.00	C(14)	32.78
H(73)	1.11	H(73)	0.84	H(73)	0.31
H(74)	2.70	H(74)	0.86	H(74)	0.36

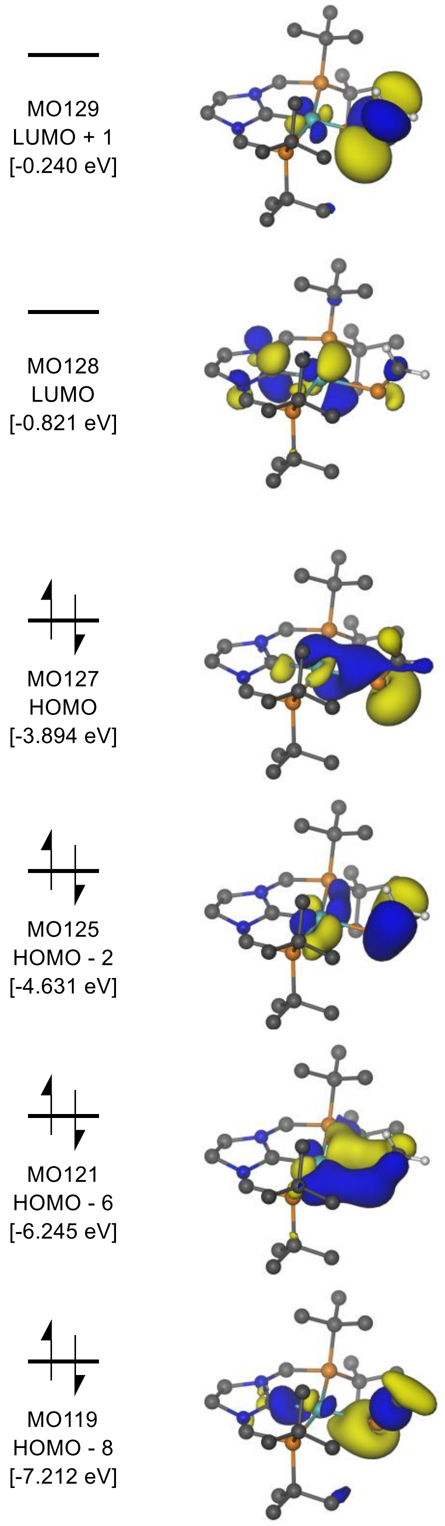

Figure S83. Relevant molecular orbitals (MOs) of [(PCP)Ir(P=CH_2)] (**4**) (*S* = 0) calculated at the TPSSh-D3/def2-TZVP level of theory. Isovalue is set to ± 0.05 a.u.

Table S16. Diamagnetic (δ^{DSO}), paramagnetic (δ^{PSO}), and total calculated ^{31}P chemical-shift contributions for the $[P{=}CH_2]^-$ phosphorus nucleus in $[(PCP)Ir(PCH_2)]$ (**4**), referenced relative to H_3PO_4 (δ = 0 ppm), and calculated at the TPSSh/ZORA/ZORA-def2-TZVP level with a SARC-ZORA-TZVP basis set on Ir.

**Nucleus**	**δ^{DSO} (ppm)**	**δ^{PSO} (ppm)**	**δ^{calc} (ppm)**	**δ^{exp} (ppm)**
P(2)	-11.59	574.30	562.71	536

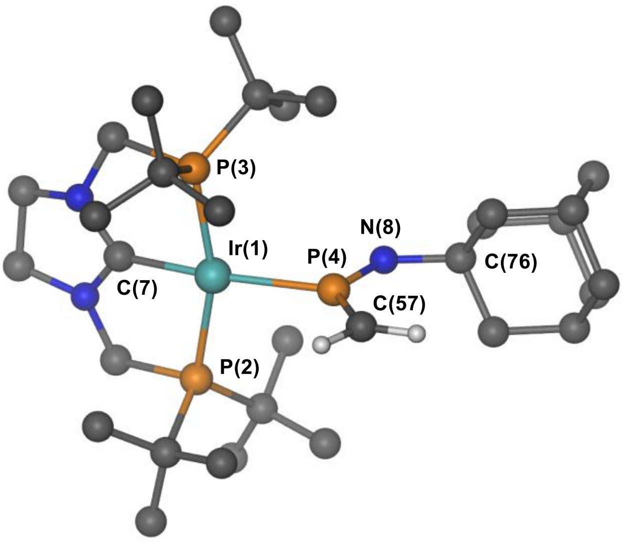

Ellipticity of electron density for P(4)–N(8) bond in [(PCP)Ir{P(=CH_2)(NAd)}] (**5**) is 0.2829, and 0.5710 for P(4)–C(57) bond.

Table S17. Calculated distances (Å) and angles (◦) of [(PCP)Ir{P(=CH_2)(NAd)}] (**5**). The schematic picture corresponds to the optimized singlet (S = 0) equilibrium structure. Hydrogen atoms are omitted for clarity. Geometries were optimized at the PBE0-D3/def2-TZVP(-f) level of theory.

**Bond**	**XRD**	**Singlet**	
		**Calculated**	**Δ**
Ir(1)–P(2)	2.281	2.279	0.002
Ir(1)–P(3)	2.282	2.277	0.005
Ir(1)–P(4)	2.344	2.359	0.015
Ir(1)–C(7)	1.969	1.951	0.018
P(4)–N(8)	1.562	1.576	0.014
P(4)–C(57)	1.664	1.663	0.002
N(8)–C(76)	1.460	1.441	0.019
P(2)–Ir(1)–P(3)	162	162	0
P(2)–Ir(1)–P(4)	100	99	1
P(2)–Ir(1)–C(7)	81	81	0
P(3)–Ir(1)–P(4)	98	99	1
P(3)–Ir(1)–C(7)	81	81	0
P(4)–Ir(1)–C(7)	174	171	4
Ir(1)–P(4)–N(8)	113	111	2
Ir(1)–P(4)–C(57)	123	124	2

Table S18. Calculated Mayer and Wiberg bond orders of [(PCP)Ir{P(=CH_2)(NAd)}] (**5**) (S = 0) at the TPSSh-D3/def2-TZVP level of theory

Bond	MBO	WBO
Ir(1)–P(2)	0.9002	1.070
Ir(1)–P(3)	0.9135	1.075
Ir(1)–P(4)	0.7829	0.973
Ir(1)–C(7)	1.0590	1.277
P(4)–N(8)	1.6428	1.928
P(4)–C(57)	1.5885	1.918
N(8)–C(76)	0.8607	1.340

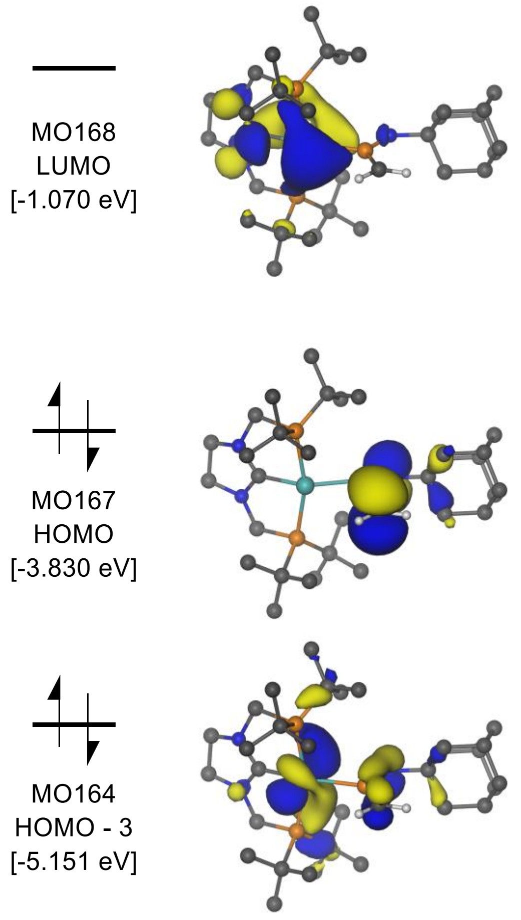

Figure S84. Relevant molecular orbitals (MOs) of [(PCP)Ir{P(=CH_2)(NAd)}] (**5**) (S = 0) calculated at the TPSSh-D3/def2-TZVP level of theory. Isovalue is set to ± 0.04 a.u.

9.3 Scenario A – Rotation about P=C bond

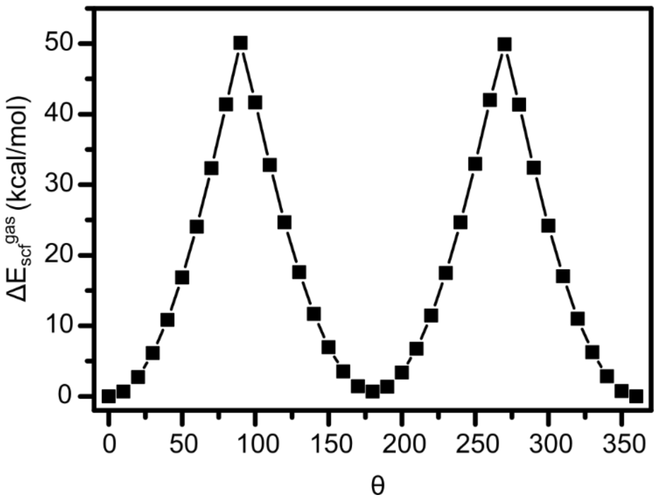

Figure S85. Calculated potential energy surface for Scenario A, rotation about P=C bond, in the gas phase. Scan was generated at the TPSSh-D3/def2-TZVP level of theory. For each dihedral angle, the geometry was manually constructed, and the energy was evaluated as a single-point calculation, without geometry optimization.

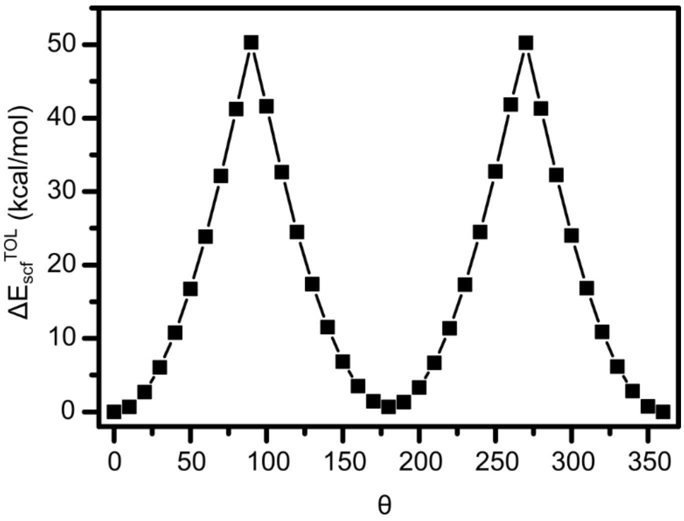

Figure S86. Calculated potential energy surface for Scenario A, rotation about P=C bond, in the solvent phase (toluene). Scan was generated at the TPSSh-D3/def2-TZVP/SMD level of theory. For each dihedral angle, the geometry was manually constructed, and the energy was evaluated as a single-point calculation, without geometry optimization.

9.4 Scenario B – π-bonded intermediate

Table S19. Calculated relative energies (kcal/mol) for **Scenario B**, formation of a side-on bound π-complex, at the TPSSh-D3/def2-TZVP level of theory in gas and toluene phase relative to the energy of [(PCP)Ir(P=CH_2)] (**4**). Transition states were located using the Nudged Elastic Band (NEB) method. Calculated barriers ($\Delta G^{\ddagger}$) are provided in parentheses.

Species	ΔG^{gas}-TPSSh	ΔG^{TOL}-TPSSh
[(PCP)Ir(P=CH_2)]	0.00	0.00
TSB_a	20.14 (20.14)	21.26 (21.26)
I_B	18.47	15.54
TSB_b	20.23 (1.76)	21.30 (5.76)

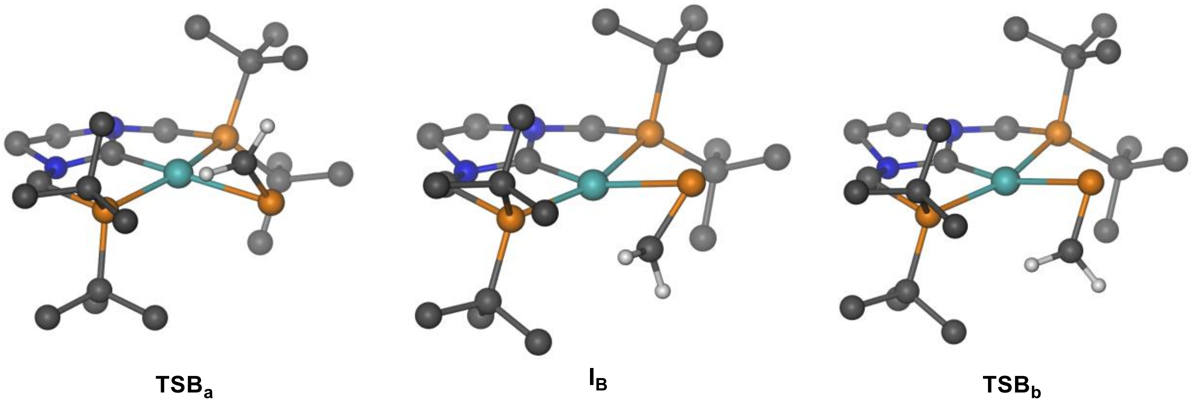

Figure S87. Calculated equilibrium geometries for TSB_a (S = 0), I_B (S = 0), and TSB_b (S = 0)

9.5 Scenario C – Cumulenic Intermediate

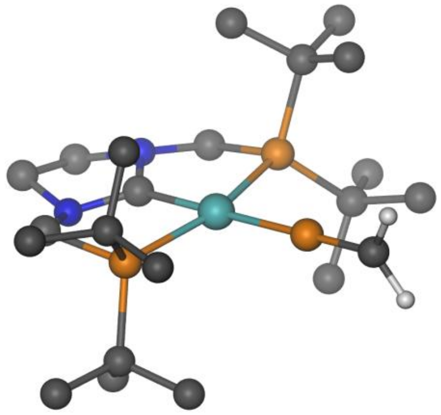

TSC

$\Delta G^{\ddagger}_{gas}$ = 14.11 kcal/mol

$\Delta G^{\ddagger}_{TOL}$ = 15.50 kcal/mol

Figure S88. Calculated equilibrium geometry for **Scenario C** (**TSC**), linear {Ir=P=CH_2} structure, together with the calculated barriers ($\Delta G^{\ddagger}$) relative to [(PCP)Ir(P=CH_2)] in gas and toluene phase (kcal/mol). Energies are refined at the TPSSh-D3/def2-TZVP level of theory. The transition state was located via transition-state optimization following a relaxed potential-energy surface scan along the relevant reaction coordinate.

9.6 Scenario D – Rotation about Ir–P σ-bond

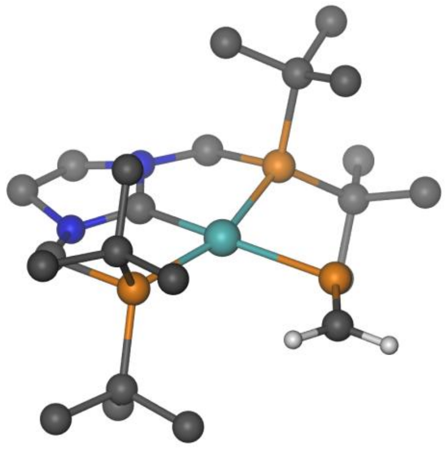

TSD

$\Delta G^{\ddagger}{}_{gas}$ = 9.30 kcal/mol

$\Delta G^{\ddagger}{}_{TOL}$ = 9.15 kcal/mol

Figure S89. Calculated equilibrium geometry for **Scenario D** (**TSD**), rotation about Ir–P σ-bond, together with the calculated barriers ($\Delta G^{\ddagger}$) relative to [(PCP)Ir(P=CH_2)] in gas and toluene phase (kcal/mol). Energies are refined at the TPSSh-D3/def2-TZVP level of theory. The transition state was located via transition-state optimization following a relaxed potential-energy surface scan along the relevant reaction coordinate.

9.7 Cartesian Coordinates

Cartesian coordinates of optimized structures (in Å) at PBE0-D3/def2-TZVP(-f)/defgrid2 level of theory. *S* is the spin multiplicity ($M = 2S+1$).

H_3PO_4 (S = 0)

```
Eh = -643.890713163739

P    -0.03083198120523    0.12237675909085    0.11646979066182
O     1.53630725425217    0.15000688307278    0.35793193224071
O    -0.41649994433169    1.65421512433909    0.26002283840129
O    -0.56998679458424   -0.48772268501183    1.47772192661546
O    -0.48046461737043   -0.54164643962953   -1.10519232596080
H     2.01192084883965    0.21361249795614   -0.47736403860640
H    -1.27548659266408    1.83199166772512   -0.13821995507036
H    -0.67495817293615   -1.44283380754263    1.40862983171828
```

[(PCP)Ir(PCO)] (2) (*S* = 0)

```
Eh = -2176.960396662998

Ir    6.90107709822962   10.93752859454421   11.77106688086251
P     5.55625788764075    9.42708235930568   12.83343655068871
P     7.89054105623219   12.21797042208722   10.16333030934087
P     8.22002634325697   11.77850584750245   13.70185839294623
N     5.19342726563733    9.11048130261413   10.21750634625927
C     6.09355669855337   10.13059380154152   10.22347753191765
N     6.32058413776574   10.46761306696671    8.92711740292011
C     4.48237218435682    8.81955907235842   11.42854344997628
H     4.26361169408989    7.75166170750748   11.50648534445968
H     3.53001865947882    9.36221927041794   11.45868391779844
C     9.75904215927654   12.34327385731695   10.03210408448155
O     6.38507098782274   13.64652327499243   14.72219831423583
C     7.05727339570943   13.88014799430028    9.88814587652294
C     6.40277754144064    7.85244455088885   13.41526809338619
C     3.82260482459985   11.39500151914261   13.51754827609398
H     4.65697339467443   12.09103296061270   13.44046563133260
H     3.07813319711118   11.81796451055996   14.19942843864173
H     3.36243612509394   11.31029288634922   12.52962979046639
C     7.46677630582376   11.26729733289596    8.61062683895693
H     7.26151806885044   11.92753560813441    7.76382107895518
H     8.32399088308418   10.63595997531300    8.34834747112061
C     4.27678407429422   10.03772958727970   14.05997072774255
C     7.08789116086462   14.66526260783169   11.19774619524195
```

H	6.62172466894044	14.09758058517138	12.00249995711936
H	6.53011862220028	15.59755135703397	11.06365751239553
H	8.09800638669701	14.92034215249966	11.51509196456097
C	5.59269590214595	13.57184911013338	9.56832465082306
H	5.47189657950950	13.06079274585466	8.61101591583943
H	5.04156517801000	14.51584860672701	9.51564812472916
H	5.14929128410612	12.95056381153227	10.34926849189963
C	4.63205382545589	8.90848197767608	8.89570093360441
H	3.68307375625919	9.45245320517420	8.78879851533836
H	4.45330577500852	7.85248832458391	8.68642212521740
C	7.50941531015517	8.21250251827433	14.40305422956241
H	8.21096467362204	8.92103227329754	13.96290516397914
H	8.05402486553928	7.30077896057408	14.66939890942991
H	7.12773821370593	8.65708163766818	15.32111219773314
C	7.13676788308862	12.86091351743789	14.28130546232128
C	10.28712005119383	10.98028928998717	10.48508489479162
H	9.99839624607037	10.77026638906604	11.51510746528649
H	11.37987595126951	10.98265182900175	10.41803333401116
H	9.91448098961706	10.16371234095143	9.86165023240186
C	5.72216960879968	9.51349296832838	8.01461971794060
H	6.45521144353737	8.75519294755825	7.70615565764649
H	5.32638609493030	9.99897232543018	7.12093588947913
C	10.27503795883105	13.40661148099830	10.99808715062359
H	10.03980011974533	14.41661094357245	10.65916156254640
H	11.36507079138060	13.32715709221686	11.06014795742151
H	9.86823756208472	13.26667072661895	12.00293523341822
C	7.06621439580768	7.25377259415448	12.17253096020488
H	6.33872926332625	6.90053091980253	11.43925366233043
H	7.67520618479815	6.39761488865340	12.47888179959205
H	7.71301152593094	7.98744515881258	11.68598045934155
C	4.94239228437402	10.24866527591177	15.41800381104444
H	5.14568386692224	9.30107692291374	15.92008051243083
H	4.26880268489453	10.82474846168505	16.05932855519835
H	5.87846392105474	10.80344056518107	15.32585450346738
C	10.27639304241835	12.62956700748807	8.62426414020118
H	9.99150148667962	11.85559733759168	7.90866408044176
H	11.37057239137926	12.65019201009156	8.65367783837793
H	9.94186345736403	13.59083900971646	8.23840382104082
C	5.47177185403343	6.81948088463476	14.03569307680105
H	5.09050686186525	7.14345537892988	15.00441676734694
H	6.03088350232137	5.89270819159066	14.20042824360888
H	4.62227940679060	6.57735855657493	13.39241300925504

```
C     3.05285137269189    9.13909058362851   14.21838736450279
H     2.51485391399803    8.99316748780019   13.27934288590715
H     2.35738090065292    9.62166896135649   14.91255986113178
H     3.29549715427357    8.16042153993633   14.62795736071586
C     7.64734652684991   14.71771511018590    8.76129575464578
H     8.64731467758607   15.08147126314458    8.99885757295214
H     7.01180582588331   15.59532159439044    8.60527019930812
H     7.69135861231218   14.17539806799237    7.81348756568357
```

[(PCP)Ir(PCO)] (2) (*S* = 1)

```
Eh = -2176.907522655391
Ir    6.59246457513142   11.25779762425761   11.87344703902768
P     5.47008469054404    9.50147009296582   12.86953205386158
P     7.78643150260798   12.32443913825122   10.21599415844337
P     7.61825629542220   12.28100295702104   13.80440822496899
N     5.03475472654651    9.26804384937921   10.26816185007670
C     5.80694400805592   10.39679329367223   10.28574723331443
N     6.01363047342958   10.74660645260881    8.96420736771308
C     4.43019061203978    8.82320905305063   11.48074467426290
H     4.37606679241829    7.73130343477347   11.51458666922534
H     3.41177997121439    9.21463617944068   11.59271282406499
C     9.65336661954776   12.06706562332348   10.18874020949049
O     5.21277738720896   13.67045358734384   13.14083780132752
C     7.32528073084533   14.11286303509998    9.83835021022735
C     6.61425620380331    8.06441520441398   13.32057954282739
C     3.31938070461622   10.97731793867261   13.74014979788073
H     3.90273724805115   11.88263330142676   13.60368720080804
H     2.56144271337772   11.16773828687983   14.50675481797592
H     2.79295710853677   10.77826792750170   12.80431074936696
C     7.24203446201842   11.42904031956599    8.67790918755764
H     7.13010223429827   12.11021461748234    7.83166375471328
H     8.02429845099487   10.70114721614547    8.42367993060778
C     4.16509793006059    9.78686917257406   14.19570222179567
C     7.62458718443798   15.02518028937301   11.02532452307915
H     7.20548381555316   14.64930696001765   11.95582367331985
H     7.16715799591420   16.00074044044970   10.83278983282542
H     8.69208186361346   15.18462443233675   11.16810605041898
C     5.80928989468899   14.08602246025561    9.62067800570954
H     5.52143495696356   13.46396045003947    8.77030686453607
H     5.47091099283365   15.10721839167770    9.42007193388163
H     5.28862526468890   13.71677589371606   10.50445433593288
```

C	4.52172659734348	8.98896604090404	8.94758882948084
H	3.53003603092957	9.44020584800265	8.79561823784828
H	4.44856368636960	7.91580723712780	8.75834661399836
C	7.72239347541170	8.58853199177579	14.22986183682021
H	8.24241831560324	9.42379895651374	13.75888711867230
H	8.43963388423568	7.78285106509062	14.41630853133595
H	7.34675357217264	8.93197548754305	15.19390278333766
C	6.07680109719624	12.85076816274538	13.05696278922410
C	9.88610005603315	10.63043587517938	10.65704175317275
H	9.48026871398551	10.47700985001940	11.65803206182413
H	10.96312216715302	10.43237501147798	10.66614854768712
H	9.41447417747838	9.89985994394748	9.99501237126066
C	5.57999367702197	9.67012042803271	8.09035994148109
H	6.41242043186697	8.98106392018438	7.87746235960491
H	5.18824823912936	10.04768233403879	7.14456776116056
C	10.31418575812531	13.01309662181303	11.18625412162764
H	10.34160572119478	14.03861430468123	10.81719766312330
H	11.34829685898211	12.69368149191289	11.34790571285271
H	9.80426517613187	13.00082602685315	12.15294250306164
C	7.25924568790369	7.61046482572556	12.00980361013113
H	6.54506214740968	7.12542132025209	11.34116763842922
H	8.04589008320838	6.88610387976986	12.24142240987474
H	7.70506387995368	8.45762843114010	11.48370406929630
C	4.85508224190057	10.14357892136004	15.50853916139240
H	5.36509873880810	9.28518725142631	15.94879401639703
H	4.10297204705477	10.48451421691597	16.22623153877078
H	5.58327550481272	10.94857074876445	15.37826590880022
C	10.29436545352852	12.24182050856622	8.81380970628542
H	9.88105243378060	11.55900448418065	8.06908010480290
H	11.36172020114786	12.01450762904720	8.89818794128499
H	10.20568931302894	13.25672005640768	8.43252094984358
C	5.93470377565685	6.86820602494111	13.97385344072861
H	5.61009068889736	7.08680848557410	14.99123420322406
H	6.65507849296548	6.04615203086743	14.03440232227928
H	5.07629658704027	6.50722578255430	13.40279693203430
C	3.21779421642046	8.60537670064178	14.40509446308191
H	2.72039960147687	8.30566294483333	13.48002808708647
H	2.43391297806031	8.91804145374099	15.10194386992137
H	3.70337099301800	7.73228620649921	14.83191697633831
C	8.00257189055623	14.68972684363785	8.59894275892385
H	9.06309236817299	14.87982404201003	8.76549775749072
H	7.53525856387473	15.65155509051523	8.36579801900807

H	7.89755706549404	14.05710289906684	7.71506383775718

[(PCP)Ir(P)(CO)] (A) (*S* = 0)

Eh = -2176.899277808446

Ir	6.14224298534685	11.63452735044423	11.77727389283734
P	5.56148231769053	9.79576526320688	13.11363829754614
P	7.66312323569776	12.50000321400970	10.14024850607480
P	7.28824251968047	11.16912997370825	13.67926588031920
N	4.84961249612241	9.48332412879370	10.37740188862070
C	5.62356148505502	10.54025494275402	10.21790335147649
N	6.08244236814897	10.61337051289611	8.94820362147628
C	4.26107823397277	9.18712726567779	11.66368196421978
H	3.96449196716914	8.13707924287995	11.67564765293248
H	3.36824826292189	9.80181694448356	11.80714815185194
C	9.53309280713531	12.30838617162869	10.30540337222778
C	7.20672169288507	14.18848286866963	9.42369887918653
C	6.51659898841328	8.11524950486778	13.14174063218009
C	3.81495775939961	11.33950721157031	14.61956543851380
H	4.64197004564863	12.04659728371951	14.69444963199238
H	3.18353658065113	11.44765059692633	15.50768217275742
H	3.21601225432306	11.60697008587701	13.74834544257929
C	7.31614740048005	11.32955659611730	8.71054619169849
H	7.28309827218366	11.85539456643697	7.75359218133362
H	8.14853740252377	10.61763494789210	8.66649700754724
C	4.31794649922696	9.90264460934319	14.54579276454477
C	7.09364574114530	15.17204151925652	10.58740312929312
H	6.37911262433861	14.82125180448543	11.33253446134040
H	6.75253998832641	16.14022467650448	10.20758774287718
H	8.04877408764670	15.32670841185791	11.09000888662263
C	5.82975098670827	14.04485159250955	8.77504142711849
H	5.86511813906375	13.45791644226231	7.85511836110113
H	5.46103609550392	15.04159303073367	8.51559992708876
H	5.10654187671848	13.58442386617586	9.44630718968790
C	4.67157445496036	8.76069317633868	9.12843817809375
H	3.67648686677043	8.95198693978948	8.71244186693207
H	4.78292076629607	7.68372630047668	9.27058256510934
C	7.69335368358044	8.15834788451502	14.11214500193897
H	8.43839015530932	8.89656830050373	13.81050371982823
H	8.17539106942445	7.17385811922651	14.11601254432057
H	7.39080490262463	8.39835546463205	15.13000764304418
C	9.76867341766837	10.91821441176279	10.90002363441987

H	9.26966220774399	10.80838897691620	11.86329250226811
H	10.84553499593456	10.78083673521389	11.04069774432096
H	9.42228489214949	10.11716306622748	10.24174522310880
C	5.78362843591898	9.36080152890770	8.25629491450859
H	6.67472795788647	8.71965443862274	8.24237918856191
H	5.46554411150055	9.53348024650886	7.22748147578042
C	10.00464999491438	13.34120682616132	11.32597808368496
H	9.97588195182852	14.35662955742281	10.92719127660959
H	11.04051861806737	13.12636985172697	11.60599958285889
H	9.39490350175551	13.29850913399819	12.23239374828764
C	7.09361690216759	7.86320585805488	11.74825690156087
H	6.32374672366883	7.59673044623418	11.02440959788266
H	7.79437288895279	7.02365037917216	11.80530165180051
H	7.63941363925885	8.73382985030475	11.37869203189578
C	5.01735495192864	9.57502189429076	15.86411890551118
H	5.32502867192686	8.53053120167748	15.92370825785902
H	4.31802058809929	9.75768835559810	16.68634358066203
H	5.89653877766477	10.20270583955472	16.00705678562350
C	10.34469385222187	12.41317343672818	9.01601995254814
H	10.00536427140327	11.70548314634747	8.25575789407340
H	11.38705003971380	12.16337309243821	9.24041497833366
H	10.33484721662762	13.40926950116324	8.58138211629978
C	5.64664476811047	6.91833512769205	13.52020310557737
H	5.32167969877784	6.95531297200351	14.56000675755824
H	6.24211023819643	6.00649630199287	13.40287328415136
H	4.76639349841248	6.80885371735576	12.88747574552600
C	3.10978543623668	8.97798676504175	14.39281245062938
H	2.47432594748938	9.25649977906143	13.55259484358333
H	2.49846294111815	9.06228861660994	15.29697340861550
H	3.38605606269811	7.93067935026692	14.28464870036752
C	8.17074343115729	14.74100884261550	8.38163590916642
H	9.13034430366209	15.01841026047866	8.81775056470296
H	7.73809969903183	15.64788390383287	7.94614327735123
H	8.34805168296347	14.03957029809356	7.56334172087276
C	4.62075185505021	12.71758528204044	11.74430226817242
O	3.69555506899888	13.40292888270938	11.81919886495055

[(PCP)Ir(P)(CO)] (A) (S = 1)

Eh = -2176.916828986534

Ir	6.08562118049942	11.63613682252129	11.80992263827242

P	5.34475511022955	9.69941797349675	12.85026396734065
P	7.62117246086447	12.36723354993283	10.23567829904456
P	7.33857005720558	12.36251551103363	13.70688043986563
N	4.67855014456069	9.50507441733267	10.28651039501584
C	5.50624476300577	10.55821629745334	10.22855814481644
N	5.95507468096759	10.67265825163020	8.94674293474724
C	4.15040067473995	9.07892926982945	11.55045028859320
H	4.04319468805438	7.99132230954776	11.56305191915881
H	3.16857244742926	9.52136619795143	11.75008367369107
C	9.48854354346903	12.11308622507387	10.40762401010335
C	7.27596230230311	14.11222191005509	9.58702337957070
C	6.55176468346690	8.24504661143652	13.03506135592325
C	3.46290888892042	11.10491940552574	14.30546123265655
H	4.12831328568970	11.96385161094320	14.37622093719681
H	2.76377405391631	11.13638260051958	15.14709246593684
H	2.88315652904267	11.20118678780586	13.38513973316600
C	7.23779262889004	11.29889637123460	8.75435187383296
H	7.27116989516526	11.86351241002395	7.81981249994609
H	8.01070916983281	10.52154963681034	8.70124479449282
C	4.24427005778815	9.79561019804286	14.37912195027386
C	7.25833399303673	15.08062277730677	10.76763285956897
H	6.56363805259560	14.75291001440188	11.53999077309831
H	6.94166688758401	16.06696141045436	10.41460079180179
H	8.23919539454712	15.18887681064375	11.22920787312007
C	5.88029456171485	14.07999379314339	8.96198284479141
H	5.85741490112476	13.50734272492316	8.03234355983376
H	5.58133253695184	15.10514544931817	8.72489256564488
H	5.13977432830626	13.65989998746837	9.64062821308997
C	4.48266289339304	8.86470713818353	9.00361441599372
H	3.50174981109006	9.11443017445832	8.58293409669533
H	4.55464016058996	7.77690564833382	9.08304845466816
C	7.73430323555238	8.69373674692707	13.88984499848170
H	8.11698264170562	9.66015156744581	13.55705169005125
H	8.53545038285780	7.95242291096061	13.80731877361394
H	7.47490749708830	8.78977102881016	14.94271808926251
C	9.67258748625190	10.75616953104956	11.08105812330993
H	9.16844487407179	10.73283472459993	12.04674278297169
H	10.74234053522387	10.57845250570588	11.23042512476063
H	9.28181231537992	9.93667783502953	10.47280341414709
C	5.62618784795263	9.47102606797474	8.18794002441780
H	6.49556253742970	8.79969230853437	8.15594724573936
H	5.33461778717388	9.70688337320498	7.16375459639032

C	10.06254765820420	13.18355787166696	11.33255888986885
H	10.08627283429051	14.16699379087548	10.86214077832136
H	11.09320607054239	12.91523439663450	11.58560074241082
H	9.49580571947940	13.24957993858211	12.26554315205577
C	7.07674899148008	7.94430413717358	11.63119790689892
H	6.31303033052935	7.49405344739654	10.99245092255498
H	7.90322356271064	7.23211450250514	11.71117862405133
H	7.44532425654883	8.85479663568082	11.15581766142049
C	5.11206127956447	9.84402422130409	15.63459516611756
H	5.57410425611028	8.88256882150912	15.85973559151046
H	4.47922922993849	10.11087031089502	16.48668408820414
H	5.89534505521085	10.60225942320238	15.54795741265348
C	10.26371269934577	12.09830071990202	9.09024527708514
H	9.91581821141704	11.31997897689999	8.40758136452017
H	11.31253435285659	11.87723041777437	9.31321047259807
H	10.23625461407530	13.04986173731622	8.56596834443839
C	5.95818485721849	6.96233439677297	13.60568997922100
H	5.68429495071436	7.06128839054848	14.65568349217021
H	6.71029547408449	6.16909137084918	13.54233356035443
H	5.08298162849549	6.62087957832254	13.04760257045668
C	3.23738033896839	8.64916075565358	14.47829344692029
H	2.52436662986163	8.65857788584740	13.65143974696680
H	2.65978111298741	8.77784652558469	15.39898267101035
H	3.70244355492671	7.66730156098427	14.51966485614008
C	8.25281968033009	14.61692155182195	8.53169835758320
H	9.24254733370855	14.81360520368911	8.94313065073592
H	7.87562230738485	15.56341419060685	8.13120360067536
H	8.35499836494431	13.92831073535948	7.68962861781449
C	4.76051927265498	12.96583999130963	12.05657125344797
O	3.91777371775233	13.74772830225137	12.13519305269451

[(PCP)Ir(P)] (B) (*S* = 0)

Eh = -2063.647117642298

Ir	6.96007029549233	10.91506629370594	11.80191964802222
P	5.58973189869431	9.39369638041120	12.79517124376289
P	7.85541773214992	12.20095913571373	10.15243406409318
P	7.95270037438327	11.75632203700124	13.54738434926773
N	5.27037919703852	9.00993261299448	10.18115207297387
C	6.11054640005129	10.09442744854576	10.20209097376169
N	6.24446454690647	10.50181287990404	8.90303198386996
C	4.57019568585469	8.70878444938254	11.39380728950541

H	4.40114740120624	7.63430561489512	11.50084442420500
H	3.59257058838968	9.20752677695849	11.41021535404166
C	9.72843278053811	12.26066035585041	9.99950207546318
C	7.07229420374308	13.90676917176971	9.98725239963345
C	6.45344140933023	7.86946731527734	13.48924154405280
C	3.75920200626500	11.35120410384351	13.32177350005140
H	4.57090981105515	12.06793560346839	13.19223692258203
H	2.99017729521272	11.78004762942763	13.97275911006625
H	3.31026730696647	11.17698683568975	12.34082600221130
C	7.35993130920730	11.33552352592650	8.57798944929472
H	7.10767285825956	12.04733005634910	7.78740433510117
H	8.21390914402533	10.73787234893712	8.23485739028528
C	4.27353995374361	10.06003452978577	13.96148908576250
C	7.19062048417417	14.65785772563440	11.30963170548553
H	6.81210912872995	14.05252934342840	12.13684014505401
H	6.60044388145794	15.57757355139243	11.24610816494099
H	8.21800068640725	14.93940370587924	11.54083427196866
C	5.58575375565129	13.64371579516262	9.73357773318672
H	5.40297870583089	13.17732263104930	8.76321344876239
H	5.05562569021256	14.60068282280872	9.75156952510351
H	5.17483873199524	12.99415824954917	10.50952388921639
C	4.64637256522929	8.86467289484587	8.87970159619364
H	3.68633135073830	9.40127613623506	8.84004368136743
H	4.47214839146054	7.81762576593272	8.62512350236229
C	7.49771294785895	8.28171074873934	14.52101459865889
H	8.17498638677081	9.03676085055229	14.11265336842907
H	8.08713407822052	7.40135856710847	14.79589837836414
H	7.05254016378274	8.67897920094103	15.43323156003435
C	10.22051899509771	10.84058396234104	10.28348602104403
H	9.91869431595733	10.51690350476499	11.28043051592481
H	11.31305294966780	10.82854460993767	10.20964682995832
H	9.82813072407036	10.11496797533043	9.56678775785885
C	5.68381916876290	9.53132798929530	7.98530043070516
H	6.44555778278140	8.81052644049311	7.65247861158356
H	5.24595987632547	10.00706613591147	7.10558955317469
C	10.29718612748076	13.18686810377477	11.06690646050478
H	10.08634911375913	14.23643476130761	10.85882016288301
H	11.38395774806899	13.06658189348613	11.10209821528091
H	9.91098603787530	12.93517259572677	12.06007645678858
C	7.19338530029971	7.25843953618678	12.29669716967428
H	6.51335106648989	6.86802785129172	11.53682045906063
H	7.80835470224483	6.42761333418021	12.65575069668491

H	7.84072728899990	8.00186707974908	11.82599897605016
C	4.91031198511148	10.41230057640900	15.30027576392513
H	5.19130779141057	9.52611085654434	15.87053556633903
H	4.19037100181356	10.97774786789730	15.89942626971506
H	5.79187313688071	11.04698350801158	15.16379766124245
C	10.22460759074541	12.68819262383948	8.61965516967700
H	9.84731336832131	12.04244154233258	7.82392052605747
H	11.31607325340134	12.61002939335933	8.60280550974836
H	9.96664906279299	13.71656457268188	8.37578485807900
C	5.51453569868485	6.83319899477745	14.09358345942939
H	5.09911330519928	7.16707157750790	15.04483642301330
H	6.08046479625503	5.91763265739863	14.29212400433533
H	4.68936717649698	6.56758998650829	13.42847401269385
C	3.09276107790049	9.11526232732252	14.17726122500047
H	2.59554389168238	8.85013754540710	13.24179661084454
H	2.35017311693563	9.62413895320269	14.79957562206273
H	3.37074635089537	8.19571460745884	14.68755738647383
C	7.63493138398044	14.75890080206597	8.85645131031468
H	8.64187098401696	15.11529545390493	9.07593227872321
H	7.00014595237011	15.64183123896751	8.73261104633658
H	7.65306873018959	14.23477704552808	7.89769219167552

[(PCP)Ir(P)] (B) (*S* = 1)

Eh = -2063.652298861928

Ir	6.83951972592904	10.99753700225794	11.79002113847637
P	5.54445367425267	9.44110135684696	12.82648479616027
P	7.85035724124632	12.22907873109293	10.16832904759735
P	7.77578624489398	12.01035581477351	13.66682493579023
N	5.15798710640613	9.11435868304338	10.20436800216770
C	6.03436116851468	10.14948185977925	10.20385724548139
N	6.25344036477687	10.49473080763367	8.91056855685548
C	4.47344068534298	8.80043517521491	11.42633635168854
H	4.28707348404155	7.72614297139098	11.50565897741006
H	3.50613041334878	9.31504448240133	11.47053440116360
C	9.72483421089799	12.26185003195844	10.07357501799067
C	7.09750571270080	13.92676425762465	9.87167434663361
C	6.46585676322645	7.90189132251844	13.39135585196592
C	3.75054797987758	11.33705904642802	13.56428289274575
H	4.57156706857454	12.05134250418063	13.48333094241479
H	3.00153666846274	11.72508232597929	14.26257951837069
H	3.28179761981262	11.25606216781359	12.58002489491830

C	7.40417524739361	11.29602872264436	8.60342153787971
H	7.20044018984521	11.96979802493659	7.76714548024222
H	8.25476629862344	10.66211499321778	8.32593724169344
C	4.25613527416394	9.98691031370187	14.07715735194902
C	7.16631202749876	14.73381610866637	11.16626900753441
H	6.72955354834439	14.17452019960302	11.99573761581870
H	6.60463267007255	15.66406115028142	11.03263484052046
H	8.18649196739269	14.99532029554456	11.44480900339717
C	5.62016998584430	13.67082782725128	9.56378956164037
H	5.47428874738222	13.15144002212383	8.61439420888371
H	5.10347411636152	14.63357892923328	9.50173756259599
H	5.16064699619172	13.07446410487728	10.35537634944568
C	4.59987102779696	8.89326475073702	8.88278475246826
H	3.63738473570986	9.41264383001565	8.77449363283708
H	4.44778146910439	7.83261737166533	8.67554844643656
C	7.57651398219507	8.31891955168125	14.35311537390985
H	8.20913151581266	9.08434345252412	13.90018580408523
H	8.19093185777656	7.44291417386018	14.58558866898548
H	7.19323315601203	8.71857511206500	15.29123223535570
C	10.17162514226785	10.86489527694880	10.51005557584842
H	9.81660899731887	10.64277028501170	11.51778431535561
H	11.26568864308012	10.82132085797027	10.49438966829853
H	9.79533163807293	10.08376916805507	9.84454547669077
C	5.67542170461890	9.52385499020850	7.99912357912874
H	6.42653395367390	8.78240207131754	7.69254577897993
H	5.26739467828437	9.99625082352633	7.10382621454370
C	10.26015446925216	13.27419078202990	11.08224000916728
H	10.09506740729289	14.30237413291405	10.75594288749672
H	11.34028974509332	13.13253594492664	11.18895594546821
H	9.80233442500166	13.13782154523973	12.06664710961955
C	7.13088573057225	7.33146130635361	12.13641183406867
H	6.40539001078344	6.94884291201279	11.41558720709256
H	7.77994605108490	6.50110177185064	12.43144982607875
H	7.73746625636168	8.09288438677614	11.64076736902249
C	4.93105555185842	10.20915130336557	15.42786100964170
H	5.20141570618190	9.26881735381641	15.91061197158654
H	4.23526151594073	10.73247902277961	16.09122343426169
H	5.82960160877246	10.82517621949843	15.32722643213910
C	10.29584867748168	12.55583461985011	8.68856959818401
H	9.98185612809825	11.82269901098916	7.94278988877571
H	11.38800317097081	12.50769853147552	8.74402249766557
H	10.03110269052617	13.54665150806837	8.32442630807633

C	5.59458131000905	6.82909719176613	14.03018460179114
H	5.20893150817507	7.13939334577544	15.00181701677379
H	6.19883908307903	5.93062668703182	14.19267594651368
H	4.74991348045518	6.54269574463625	13.39832921484867
C	3.06929988847980	9.04027600895989	14.23606075206746
H	2.52609224220128	8.89059845722274	13.30079161655117
H	2.36353444222711	9.48195844201119	14.94691187428389
H	3.35483323320003	8.06421274200425	14.62377943867207
C	7.72252886922616	14.71799199622558	8.73067626050905
H	8.73175234255108	15.05386627058397	8.97174900424588
H	7.11907060672524	15.61273409400722	8.54578409080981
H	7.76006814525310	14.15222871922167	7.79640465223212

[{(PCP)(OC)Ir}$_2$(η^2,η^2;μ_2-P_2)] (S = 0)

Note: The calculated Ir····Ir distance of the optimized singlet [{(PCP)(OC)Ir}$_2$(η^2,η^2;μ_2-P_2)] structure is 2.823 Å. QTAIM analysis shows a bond critical point (BCP) along the Ir····Ir path of a (3, -1) type. The characteristics of this BCP: $\rho(r)$ = 0.0567 a.u., $\nabla^2\rho(r)$ = +0.0717 a.u., $H(r)$ = −0.0133 a.u., and sign(λ_2)ρ = −0.0567 a.u. indicate an attractive bonding interaction with partial covalent character. However, it is not strictly defined as a classical metal-metal bond.

Eh = -4353.993384649175

Ir	11.25674707595762	5.82314845984813	3.71647760921332
P	12.72523661206610	4.56460354453545	2.41643673563914
P	12.56972740677963	9.65755855262613	6.94306361367468
O	10.98217103692859	4.07768412148993	6.17324826808373
C	11.06019085086666	4.71042217622539	5.20888313061753
N	13.28397956306758	7.79880162994707	5.00502817131836
N	14.18167151691366	6.07878566287313	4.04664922849246
C	13.00343292876201	6.68555824913900	4.31333696950825
C	12.06157599544541	5.37372104665057	-0.18353525638692
H	11.64356227633350	4.40649214905516	-0.45584729012533
H	12.37148344647895	5.87648228616635	-1.10561262616493
H	11.26886792189767	5.96736433366714	0.27180499683375
C	13.27137106862743	5.26090991266564	0.74002331180609
C	13.81102641463058	6.67128005934955	0.98680694354584
H	13.08559320145685	7.28999107886940	1.51515724099590
H	14.01096018010304	7.13392663954264	0.01534840172203
H	14.74717149116740	6.66263511651908	1.54874262123101
C	12.63844661714102	2.67738265451673	2.31381254109659
C	12.36031741169910	8.87076603183506	5.26751584475453
H	11.35158417465213	8.48309390829097	5.12848902136056
H	12.50766139471628	9.65686030807960	4.51886382151678

C	11.38660938751709	8.65799228905951	8.01639161700936
C	14.71733270622509	8.05796608368437	5.06653971872709
H	14.99988317502180	8.43275095087619	6.04967770707993
H	14.98675716750432	8.81345984232311	4.31689425967095
C	11.68393218424095	9.01275626217090	9.47322468953117
H	12.74200466247348	8.87158409723661	9.70594666862006
H	11.10078212089043	8.36012314135096	10.13157764431210
H	11.42149086550675	10.04430549416446	9.71229074487035
C	14.25911431445152	4.78317181785498	3.44377104137410
H	14.27557783844909	3.99143751905753	4.20299238233559
H	15.17693079660278	4.70551173511203	2.85459472006401
C	15.29930742515983	6.69560949057657	4.73109644110320
H	16.18018887266068	6.75301765029067	4.08853825512731
H	15.56273197216568	6.12630837801735	5.63223672357349
C	11.83529165328285	11.34943430791355	6.54188182434930
C	14.00342888331323	1.99784626966013	2.20390973827612
H	14.55568375105133	2.28101784723293	1.31145532618065
H	13.84681087317708	0.91504338354135	2.16264094027500
H	14.63192594855338	2.19437358692133	3.07432564820535
C	11.53077929096633	12.08005176607785	7.84662281653632
H	10.66643345090224	11.65778848598361	8.36026919800865
H	11.30403896740501	13.12933520956609	7.63057217092640
H	12.38334588346147	12.05512750617621	8.53033434784718
C	11.97573796482749	2.18935366400384	3.59705673296809
H	12.54052480839707	2.46137393458313	4.49075806638630
H	11.90841770259015	1.09729257405333	3.56421544483858
H	10.97228450504951	2.59840635340442	3.69810920443490
C	12.96383387317743	12.10360641362001	5.83279896027872
H	13.85378406424280	12.16967725376418	6.46234963654699
H	12.63193090994713	13.12029330439205	5.59727073188240
H	13.24978480146147	11.62838056965221	4.89132996128891
C	10.59820857324500	11.35457449202411	5.64720486198715
H	10.78440828358933	10.89544072618130	4.67526603409423
H	10.29278944963656	12.39118173185211	5.46428120191641
H	9.75558206239442	10.83390807067340	6.09749877593344
C	14.36617015775807	4.46249219450884	0.03861567375306
H	15.25039147949897	4.32066641666958	0.66424883807818
H	14.68407458595236	5.02295647645533	-0.84632605362865
H	14.02155841156092	3.48783576244120	-0.30572457711858
C	11.75804034170825	2.25907483785427	1.14144269241087
H	10.80200050083593	2.78110423557459	1.14696102188900
H	11.55481386472823	1.18630593997332	1.21699310551753

H	12.23594250739186	2.43432641347196	0.17780692705365
C	11.77655785402868	7.19714448735691	7.79079219355286
H	11.49202509816764	6.84789269464405	6.79809227507944
H	11.25925435509182	6.56946481796313	8.52326551822599
H	12.85178880578080	7.03959939639640	7.91707974477672
C	9.89790323345180	8.81264887992839	7.73879987925504
H	9.53473153421330	9.81052379780259	7.98965490128442
H	9.34336211947522	8.09967096508560	8.35978126078621
H	9.64672703314183	8.59888520033202	6.69779676458186
P	10.43885871084957	7.48047671967997	2.17273950397011
P	9.51712077260839	7.48055655506230	4.06544921574209
Ir	8.69927654885966	5.82302568767962	2.52192725637760
P	7.23096224073159	4.56454826635126	3.82224558691233
P	7.38547583338807	9.65856301168862	-0.70318688704530
O	8.97451680942866	4.07753278322972	0.06525627034311
C	8.89612809704741	4.71025442719920	1.02959007741239
N	6.67152329449799	7.79876225378313	1.23403715586783
N	5.77418487554031	6.07838515649500	2.19212286886879
C	6.95234029450625	6.68525853312765	1.92521474071252
C	7.89485249608099	5.37446823006298	6.42185866744410
H	8.31300591274544	4.40736225591587	6.69441692124220
H	7.58507221910444	5.87750873340367	7.34383012280057
H	8.68742504174949	5.96803296658437	5.96616935956040
C	6.68492056936012	5.26125474762734	5.49853441945245
C	6.14510197049737	6.67149777836056	5.25136666541585
H	6.87044312764540	7.29013205937448	4.72278501037346
H	5.94518036096346	7.13441124978386	6.22270163326573
H	5.20892490058142	6.66258978404051	4.68948251865592
C	7.31802415443450	2.67736112538697	3.92536688301150
C	7.59493827568014	8.87106697660544	0.97203104200678
H	8.60374400151041	8.48355905896210	1.11097455830261
H	7.44736105439886	9.65679607447507	1.72101960271730
C	8.56798171534444	8.65897424114040	-1.77716061458647
C	5.23809851534215	8.05753258601718	1.17246508113295
H	4.95550643028923	8.43242186304711	0.18937872755413
H	4.96841685109804	8.81282218846755	1.92222258950437
C	8.27043768762416	9.01431932239858	-3.23380395443735
H	7.21224998533445	8.87366003876878	-3.46631074326366
H	8.85314743569459	8.36164758052671	-3.89250717415241
H	8.53325516816864	10.04583569435683	-3.47260334078356
C	5.69698116170720	4.78275932529728	2.79500669451776
H	5.68057881212659	3.99101287299306	2.03579633946733

H	4.77923443458907	4.70495671951399	3.38427642566245
C	4.65644384898760	6.69498081900100	1.50765946893876
H	3.77548403748488	6.75207437612660	2.15013962034199
H	4.39326871460796	6.12572834578471	0.60641407345636
C	8.12064924690495	11.35000972884517	-0.30156148381740
C	5.95313802453459	1.99764328782620	4.03538533764966
H	5.40081019576664	2.28092869062515	4.92775775125188
H	6.10992274953627	0.91487227799609	4.07689211819813
H	5.32464219424347	2.19388967504878	3.16490395391415
C	8.42509807928614	12.08100096472772	-1.60610779330552
H	9.28919632791858	11.65864537178278	-2.12009542563979
H	8.65223047379586	13.13013016703889	-1.38971641887580
H	7.57237332444082	12.05661983199002	-2.28964196558280
C	7.98089763598567	2.18900364621059	2.64231798876178
H	7.41617396703043	2.46071423917738	1.74848312757736
H	8.04829690939004	1.09695725190061	2.67548966894974
H	8.98432623491563	2.59809830712100	2.54122399872581
C	6.99258494196726	12.10431398949182	0.40813681003551
H	6.10249339888287	12.17098958687879	-0.22115045541841
H	7.32493240073315	13.12076987354892	0.64403332928620
H	6.70670186735883	11.62877111002620	1.34946926439804
C	9.35801272952245	11.35434173490346	0.59272670249081
H	9.17198067188104	10.89485641339552	1.56452841792056
H	9.66384090582855	12.39076709887655	0.77599896956497
H	10.20031745986376	10.83358588489392	0.14193052402584
C	5.59034762154211	4.46289646873626	6.20037305929846
H	4.70601973923179	4.32077464098119	5.57495618468548
H	5.27254288922836	5.02357158464440	7.08521925036212
H	5.93517163298500	3.48838583572843	6.54492174357911
C	8.19835413264720	2.25956495557929	5.09797492577739
H	9.15426842417203	2.78184202991093	5.09246895193245
H	8.40186098851106	1.18682625023371	5.02273781677618
H	7.72025003487444	2.43495477108427	6.06149120963411
C	8.17757630337472	7.19819026226687	-1.55197920519709
H	8.46212029457992	6.84855415302231	-0.55942840751348
H	8.69459480133339	6.57056145266977	-2.28469335088558
H	7.10227940003970	7.04105211074291	-1.67820652992676
C	10.05679893532041	8.81299878587711	-1.49983592066803
H	10.42029350378242	9.81078275518782	-1.75058649888680
H	10.61094107451764	8.09992549299105	-2.12105700013010
H	10.30812446544309	8.59895626831370	-0.45892873650295

[(PCP)Ir(P=CH$_2$)] (4) (S = 0)

Eh = -2102.950174930688

Ir	1.13763961426300	3.10930922699859	5.03384040248351
P	0.94934428940966	1.89402835250161	3.12145285514571
P	3.10965331327734	4.28987864695828	4.50894472411734
C	-0.33875027564997	1.93460863170081	5.55653579839158
N	-1.01979975089298	1.96488075374544	6.73374050807617
N	-0.90039963313446	0.96592872991216	4.79391095265410
C	-0.92798290420153	3.15470273839942	7.53239293407383
H	-0.98274037800983	2.91868317358197	8.59803533523115
H	-1.75184649610345	3.83916609922527	7.29647135959944
C	1.82979354458146	3.47429765724577	8.47057253996359
C	-0.19299210555909	0.50069452144659	3.63734034530532
H	-0.88732606294882	0.22067293196284	2.84069160456044
H	0.41880026670673	-0.37681518901987	3.87749789022416
C	2.97009037844170	5.79710371305977	3.80556180291170
C	-0.01825102212460	2.75116955394276	1.75590377786401
C	-2.22090890548863	1.15131047672790	6.66570252017632
H	-3.09147912081890	1.76689825734217	6.39805797239631
H	-2.42820273495359	0.64912508455786	7.61209492138569
C	-1.86150500526225	0.18017753636429	5.54286139275178
H	-1.39861099728986	-0.73551830298983	5.93601115374445
H	-2.72226143213494	-0.10075415836047	4.93306190737429
C	-1.38371725683594	3.08876884707408	2.35969995022418
H	-1.93863998275587	3.70571946758105	1.64615304182189
H	-1.26472588840077	3.64658267517395	3.29120352947476
H	-1.98074975924015	2.19777684426371	2.56533820581025
C	-0.22131155814144	1.92975073283452	0.48991206248581
H	-0.67271989004997	0.95503736585635	0.69117395994900
H	0.71146403929302	1.77363595743379	-0.05291168210517
H	-0.89936189626178	2.47055217795548	-0.17833408253542
C	3.25854220764928	3.89664472795687	8.13832764070496
H	3.38688904692799	4.97815730438945	8.13755413260278
H	3.56732841494950	3.52479833102942	7.15984365684521
H	3.93332417384187	3.47996860055717	8.89314552589353
C	1.44430204008625	3.99892705067130	9.84739803526920
H	2.07931262001724	3.51998973857091	10.59969351308664
H	0.40679109744282	3.77743608572729	10.10931927079675
H	1.59827328751813	5.07537389462267	9.93257314917940
C	1.78702866066344	1.94418494120251	8.47753899639989
H	2.03270654976122	1.54843489780953	7.48939820457974
H	0.80907767236987	1.55371117308187	8.76656105913182

H	2.52282409301897	1.57542869617639	9.19888813327326
C	0.67945217244243	4.06851614064161	1.42541200727042
H	0.05315689107937	4.63784953723829	0.73066617940238
H	1.65325870906436	3.92571720480597	0.95894384323150
H	0.83486942655898	4.65926144332534	2.32865403842192
P	0.68201787625190	3.99642240369614	7.07534944861330
C	2.42356316823789	0.94845361410388	2.44866021279996
C	3.30373567727043	1.89031247962716	1.63273205428477
H	2.84960828456898	2.14792919700391	0.67468565126963
H	4.25527303817693	1.39191508260486	1.42275424568827
H	3.52516191963000	2.81161992008550	2.17832797287850
C	3.21258337257523	0.49707227961338	3.67974087863348
H	3.56031734961029	1.35334078157582	4.25895146635874
H	4.08093621517519	-0.08211988222011	3.34877863184211
H	2.61954629659982	-0.13752833763945	4.34343227023679
C	2.06266114026407	-0.27819058895247	1.61446060317061
H	1.48340823242760	-1.00996297774182	2.18096431729366
H	2.98820072582494	-0.77411002365979	1.30496568118636
H	1.50834654840742	-0.02848422494028	0.71154092866591
C	0.21719096606220	5.81106855224284	7.18543562504476
C	-0.53129247243296	6.10575911396417	5.88397970669842
H	-1.45239357175673	5.52389292891138	5.79832905005878
H	0.09205913874813	5.86926777374085	5.01976928088945
H	-0.79958678059696	7.16697938587901	5.85848136818475
C	-0.67362281550661	6.17997879983745	8.36840125677022
H	-0.91004789191308	7.24725889040224	8.30728929184753
H	-0.19154092210320	6.00740960501114	9.32916885408362
H	-1.62379837030224	5.64218012647657	8.36154834298706
C	1.48833150436701	6.65558609312699	7.21320885947469
H	2.17807703550689	6.37043121253725	6.41571223550403
H	2.00208298011952	6.58425106835448	8.17294110566592
H	1.21734677041569	7.70564230884237	7.06375553727339
H	3.86832682191320	6.35408245409552	3.54999132560127
H	2.03645630935260	6.30047469213787	3.56266272734804

[(PCP)Ir(P=CH_2)] (4) (*S* = 1)

Eh = -2102.899255644962

Ir	1.05684711448605	3.20423201396860	4.98087086752277
P	0.94314931342022	1.90928692130860	3.11250097137642
P	3.01049608151596	4.50606869927650	4.42162435288465
C	-0.42604079186326	2.05638604881365	5.49412690101292

N	-1.10098260754424	2.09088644783171	6.67134272697277
N	-0.96392279857446	1.04766655400235	4.75569622031812
C	-0.97798485472219	3.25302114002997	7.50199609669608
H	-1.03629579848911	2.98449971273711	8.55992047583303
H	-1.77796164314505	3.97276863518315	7.29109795180948
C	1.80621019037478	3.39257751863011	8.40728159550337
C	-0.19447350810747	0.52922474557267	3.66143167865503
H	-0.84283987012294	0.18032171615647	2.85358171663855
H	0.42457708599560	-0.31539230793494	3.98808969986919
C	2.49195204804662	6.00940142906480	3.68219141877622
C	0.01209090208018	2.67378051188310	1.66948667699199
C	-2.25765036421460	1.21735101221994	6.65304955737705
H	-3.16665250064137	1.77537216849721	6.38835958981320
H	-2.41693093512509	0.73181528205168	7.61761374598093
C	-1.86539551879412	0.23511428087769	5.55133424802125
H	-1.34124394349848	-0.63900553595011	5.96293019160748
H	-2.72006088472295	-0.11447871627472	4.96961718645047
C	-1.38820048945541	2.99658058686352	2.19604770497107
H	-1.92439981654019	3.57040436374059	1.43387736084162
H	-1.32972952317397	3.59392661860368	3.10836242260357
H	-1.97140855816082	2.09847442203175	2.40949803079625
C	-0.10707379008221	1.78943470755338	0.43606336414323
H	-0.53798351370602	0.81101935367833	0.66264191812608
H	0.85463139044792	1.63841838227336	-0.05516769246805
H	-0.76863747626872	2.27708817921877	-0.28737944362737
C	3.24981786455785	3.73690659106459	8.04807553486859
H	3.44710214449170	4.80770737679919	8.07241714702598
H	3.50038521758631	3.37986481648399	7.04854178431228
H	3.91684554064687	3.25575392648196	8.77083827095997
C	1.48770235146659	3.89106293644408	9.81088193565378
H	2.11317277292494	3.35105223029720	10.52906322012808
H	0.44622481319835	3.71946363987418	10.09383789547817
H	1.70582128887157	4.95316813424837	9.92761228375002
C	1.67409139773823	1.86805037928663	8.36889047329259
H	1.87242025807971	1.49086196484287	7.36317356803375
H	0.68405871849225	1.52380966029609	8.67479832180388
H	2.40629719874314	1.43679453630568	9.05851252713430
C	0.68476577311712	3.99643979728151	1.31059088823664
H	0.09008236668381	4.50178321094894	0.54240403860720
H	1.69289511673048	3.86281176602179	0.91879232341083
H	0.75037001732500	4.64471944103247	2.18590305574999
P	0.66203263839642	4.03975384570582	7.06101332675629

C	2.45809991295421	0.95851482788498	2.54258354827249
C	3.34989612434723	1.87443228951112	1.70994531333415
H	2.91848650055847	2.08951097683528	0.73121878259685
H	4.31106256356379	1.37755128606864	1.54437838341505
H	3.54568514513125	2.81661234284676	2.23033550773523
C	3.20977412926380	0.59013978585615	3.82331088695975
H	3.49670720957366	1.48423970429117	4.37823266794769
H	4.11318377169975	0.03187021774340	3.55606577737349
H	2.61209438888534	-0.03867380126679	4.48818806508845
C	2.14960027374684	-0.31797420432184	1.76329779966348
H	1.55721380919329	-1.02744963708892	2.34462591227895
H	3.09415238923691	-0.81476518444948	1.51947990402575
H	1.63026520340253	-0.12681641089797	0.82602683476859
C	0.29224089129555	5.86580240281694	7.26644065393971
C	-0.46970968250132	6.25941015189583	5.99906238442900
H	-1.41457584671564	5.71814054784035	5.90504220072658
H	0.12475413189210	6.05829392567652	5.10557051868023
H	-0.70009798663879	7.32917640869099	6.04221812117408
C	-0.55326413691203	6.22230723644748	8.48604671855941
H	-0.74341017651822	7.30048126859885	8.47506826057258
H	-0.06034419699615	5.98681332657049	9.42756843381933
H	-1.52643999980914	5.72736659142191	8.47573321736704
C	1.60905366658679	6.63630057998293	7.30469387311240
H	2.28105019269606	6.32040228871308	6.50349433431943
H	2.12464182386529	6.51420513838775	8.25864295665896
H	1.40341791207652	7.70332395285698	7.17491713918581
H	2.40240461143046	6.92895159345605	4.25410215637068
H	2.45847495622630	6.12828824830661	2.60312951692447

[(PCP)Ir{P(=CH_2)(NAd)}] (5) (*S* = 0)

Eh = -2547.499179081571

Ir	8.87528319833752	5.72693860778111	11.25741680158322
P	10.90178546389425	6.27182625280549	10.36827309980612
P	6.75687436687513	4.92880160059637	11.50166814803422
P	9.20998956887699	6.53490830159109	13.44818302753370
N	7.32269272914535	4.84807459598430	8.88663985780011
N	9.28137410912525	5.64160912392149	8.34111137809513
C	8.48364771230908	5.35249880596668	9.38320244028832
N	8.77570636454378	8.04545257946890	13.56690283087879
C	11.62269321117957	7.98137680935863	10.63193156852997
C	10.41318437998398	8.92064834692325	10.62739936521376

H	9.86347588164279	8.87327014087706	9.68303160314919
H	10.77061500735435	9.94883854258541	10.75019818231872
H	9.72460282273629	8.69116904969828	11.44494631391466
C	5.44054175677727	5.99737991187937	12.30200362520434
C	10.55185903836346	6.27632921107960	8.52906835121745
H	10.51053882741540	7.31186486165472	8.17883796617061
H	11.34065906806965	5.76584872709942	7.96851992599204
C	6.16138730215952	4.84695151433618	9.73307823122971
H	5.53704275336690	3.96878336387181	9.54926325050105
H	5.55315304071862	5.73838949418156	9.53943812574191
C	12.18743396822755	4.91346265746838	10.54836849336378
C	6.66254536595960	3.13990474934305	12.06703387942444
C	12.36076959136060	4.61449261997861	12.03578309892725
H	12.77410391548755	5.45414593282376	12.59244240642603
H	13.03938727415273	3.76311253465050	12.15148771358705
H	11.40232398454908	4.36299127593431	12.49065567216608
C	5.78163531279399	7.43091853453479	11.88412482692620
H	6.76365809176070	7.73563022919932	12.25517456892928
H	5.02609879855290	8.10343083734837	12.30475473822060
H	5.76615079309436	7.55717982596517	10.79811442837643
C	11.58462903084312	3.66839204001632	9.89477261726968
H	10.59891933934684	3.45639988968825	10.31377387931620
H	12.24113013054442	2.81517398243000	10.09081019233223
H	11.48617504794727	3.76574863991032	8.81119431893756
C	7.21888952198551	5.07504587006363	7.45399313344239
H	6.57814279251811	5.94408421418601	7.25288393448875
H	6.79601358706761	4.21191716432983	6.93711982328948
C	8.67529888128507	5.34277636271681	7.05893839603325
H	9.14429473441720	4.46373265754310	6.60173380863975
H	8.77306706302367	6.18081185339152	6.36466586720034
C	5.55250226889863	5.89387370476435	13.82036364656796
H	5.19398942450017	4.93352183525851	14.19486651600448
H	4.93413730250441	6.67551681600284	14.27194225705915
H	6.57701483977434	6.04563116809511	14.16452028668983
C	7.39200393056748	3.01861957488799	13.40254753433131
H	8.41709779610758	3.37916263483487	13.31445895658668
H	7.41432843495280	1.96592202870006	13.70276702213288
H	6.91553032913348	3.58725610026576	14.19982714067641
C	12.29137241373926	8.04481289628654	12.00219210105871
H	11.64747771407550	7.65205386926298	12.79073368538611
H	12.50350171184918	9.09132096948978	12.24126021561201
H	13.24015334529284	7.50537667938490	12.01914229257544

C	7.44073193599809	2.32217527454954	11.03478126320712
H	6.95337714448710	2.30507328258985	10.05777460499733
H	7.51463941082423	1.28951575609808	11.38923166947309
H	8.44869988461987	2.72070640841833	10.90623730715120
C	9.82928618731565	5.62644658135587	14.69573043980550
H	9.99331686025923	5.98545445666685	15.70269558328522
H	10.08850081953171	4.59584241932202	14.48581674516297
C	13.53646513376191	5.21619378308415	9.91146636558983
H	13.45024142105573	5.49846685749337	8.85912449094983
H	14.16142327226896	4.31844745739142	9.95769371360440
H	14.06836936532712	6.00865218847663	10.43841291975630
C	4.01181650390621	5.67830787361251	11.86803471832169
H	3.86942374022466	5.78912980900011	10.79118324899529
H	3.33743773629787	6.39145601418253	12.35245316245240
H	3.69049162255914	4.67824636674853	12.15396625323862
C	12.61244314734794	8.43289479883248	9.56062355109312
H	13.50171608803539	7.80701240881340	9.50930023630558
H	12.93950174712996	9.44949318745282	9.80058228958036
H	12.16310257774589	8.47213363914826	8.56639977717931
C	5.25088638487349	2.58062137521782	12.18517500611332
H	4.69669592553580	3.03786211300195	13.00496293199633
H	5.31084011030680	1.50704109037153	12.39059423372402
H	4.67447867916234	2.70523668089445	11.26526091885890
C	8.81340197903007	8.90735272960242	14.72162286031990
C	10.23858027943601	9.10930465400350	15.25627188748895
H	10.85801437830478	9.49774557381209	14.44088363243751
H	10.66181450859172	8.14375963483807	15.54734457341458
C	7.92104570811949	8.39831003263351	15.86241592244426
H	6.90395123755034	8.27915354343924	15.47404577033453
H	8.25918611511677	7.40782196150226	16.18040008446207
C	7.93269464676032	9.36889167523599	17.04131955373684
H	7.29796277241848	8.97657760539080	17.84373817807377
C	9.36342453335322	9.52680638057077	17.55375354953697
H	9.74719290873291	8.55798364181631	17.89228011665650
H	9.38302382833225	10.20163488119862	18.41728821600995
C	7.40255292114825	10.72702225762667	16.58690126614015
H	6.36972346921346	10.62595078555356	16.23515612445081
H	7.39052966044898	11.42899824481051	17.42873513064415
C	10.24935462045257	10.07700295142731	16.43738969426137
H	11.27461628973999	10.19179582040954	16.80649136319507
C	9.71290247467133	11.43326241500219	15.98419691017386
H	10.35352826014092	11.84255207113410	15.19513047900833

H	9.72969105697858	12.14536123845523	16.81737372649189
C	8.28619163655376	11.26668602600457	15.46376536348710
H	7.90246104455155	12.23630455105743	15.12768027151033
C	8.28122030881903	10.28205035985023	14.29645781725585
H	8.90060834590752	10.65209863673019	13.47247872587191
H	7.26781997786232	10.15376051475477	13.90137880656836

TSB$_a$ (*S* = 0)

Eh = -2102.916160332869

Ir	0.26966099555868	0.33079266912080	-0.18336718038445
P	0.19356369045237	-1.08047356074901	-2.00525753343110
P	2.29483361594876	1.17153774353684	-1.10347066989717
C	-1.22765006738403	-0.79184073146118	0.33247659029907
N	-1.83638148893896	-0.82342950228233	1.55589410295594
N	-1.78387547624866	-1.82602661907082	-0.38628027084524
C	-1.70093097188755	0.30160310923827	2.41710212409287
H	-1.70191084125543	-0.00295309919383	3.46729227276820
H	-2.52090587138765	1.01478011756803	2.26987763163730
C	1.14648269538261	0.46228883006238	3.17716553994890
C	-0.99028018536839	-2.39076518822579	-1.43344540322563
H	-1.61195875795867	-2.76916049728068	-2.24861352618174
H	-0.39704467437694	-3.22831256236369	-1.04441243572431
C	1.33502428492040	2.55866148410973	-0.89472097503782
C	-0.68910619253818	-0.35486991693019	-3.50550048499347
C	-2.96111840164037	-1.72887008431430	1.58512339509731
H	-3.90206772136692	-1.20767251662199	1.35505493428694
H	-3.06330507028760	-2.21790568127324	2.55617295566187
C	-2.57075373958356	-2.69059007483126	0.47323139855377
H	-1.95678949969612	-3.51720032059159	0.86218148478473
H	-3.42928267679370	-3.11098922571403	-0.05298609331728
C	-2.05556407633023	0.10928283004964	-2.99785194710054
H	-2.56575847750224	0.64340524613333	-3.80562326866446
H	-1.93983732636626	0.78074567299678	-2.14418305229481
H	-2.69136889420383	-0.72290878615900	-2.68885747932601
C	-0.88966419099969	-1.33029944766408	-4.65856060446966
H	-1.39092129633830	-2.25064025666059	-4.35039326147770
H	0.04925260090762	-1.59418169707948	-5.14571905239714
H	-1.52398296572559	-0.85519765296213	-5.41388613023355
C	2.53978132075449	0.95002062549368	2.79043876204645
H	2.67411995342992	2.01552618745900	2.97789295992281
H	2.73477377509582	0.76111318388845	1.73159659887667

H	3.28171590367554	0.41052993638877	3.38747082686867
C	0.86934959316903	0.79987202013180	4.63656956442102
H	1.56926979928392	0.23765666760742	5.26297134246008
H	-0.13927804238482	0.52312799034584	4.95316307359263
H	1.02407087642939	1.85678226419854	4.85447525382316
C	1.11398203296886	-1.05885978225212	3.00808126859684
H	1.30775289474282	-1.33530452883218	1.96970193238097
H	0.15341172299848	-1.48759106898508	3.30253400365086
H	1.88855312087381	-1.49635919029043	3.64587119898875
C	0.06712659684884	0.87536233719888	-3.99824463081027
H	-0.50295649112087	1.33206443930349	-4.81398334397851
H	1.06190124920984	0.63785181112461	-4.37282748781299
H	0.18182012286882	1.60744680441255	-3.19972696114146
P	-0.10863151185998	1.14039553084245	1.93933064899110
C	1.65931071791036	-2.14717505917995	-2.54302487805562
C	2.56915593357092	-1.40836329486771	-3.52331257937102
H	2.09945844736395	-1.28719620346517	-4.50051347562697
H	3.47630882140478	-2.00411444620827	-3.66904552274398
H	2.85842024059368	-0.42586770448001	-3.14756800098820
C	2.44054592483367	-2.45067430061587	-1.26428711615236
H	2.81069670487711	-1.53804739609197	-0.79719965307096
H	3.28852139811301	-3.09852105979864	-1.50989682120236
H	1.82401687210988	-2.96879927168174	-0.52502601186005
C	1.24385243382082	-3.47450940916680	-3.18084244199525
H	0.69039720500281	-4.11910355868034	-2.49681083886522
H	2.15429348718799	-4.01433750743206	-3.45983613227933
H	0.65288816386734	-3.34440246646291	-4.08546650789947
C	-0.56370434775063	2.92689990467318	2.37522705918118
C	-1.43307467075660	3.44274074246314	1.22599107329496
H	-2.34755628143356	2.85499796267263	1.11955613783759
H	-0.91750949295083	3.41207397137344	0.26769676045113
H	-1.72390216899523	4.47636089612969	1.43978257011028
C	-1.37816159820987	3.05860769553340	3.66461058124863
H	-1.60237959010816	4.11902066563286	3.81955178006033
H	-0.84246419263318	2.70760348835605	4.54358447547223
H	-2.33532742352066	2.53766347454784	3.61511145397697
C	0.68261885508601	3.79955386688573	2.51963025657744
H	1.38327756325427	3.69914571414349	1.69107784340678
H	1.22178831057161	3.57819347817919	3.44180507510579
H	0.37354925336633	4.84852948125894	2.56630150802642
H	1.11262845822885	3.24765796189394	-1.71390786227612
H	0.99722903921987	2.92361686496562	0.06305319567557

I$_B$ (S = 0)

Eh = -2102.922060645339

Ir	1.24740812772262	3.11127257165874	5.04152497026439
P	0.99964351138534	1.94445591568299	3.04599528811215
P	2.77186748756090	4.29056484878239	3.63569529055099
C	-0.26091337970912	1.97414118504915	5.51927950820118
N	-0.94202680070479	1.95966199471819	6.70964566582195
N	-0.86424845739065	1.05313543616659	4.71748609028304
C	-0.86109046193325	3.13739377120390	7.51943382522396
H	-0.95272487484438	2.89277419631254	8.57927735210999
H	-1.66479955199582	3.83931894588957	7.26351659314818
C	1.79212313001772	3.40075784557454	8.62448567620475
C	-0.10715561085908	0.54934484049923	3.61809374715007
H	-0.76144084164113	0.17812029511776	2.82684566678728
H	0.54155231565494	-0.27272193387855	3.94301325572827
C	3.28391478004536	4.06722666647236	5.27916127483216
C	-0.05301548389951	2.75676124147803	1.70329925725588
C	-2.17072655967390	1.19897255342508	6.59057780059659
H	-3.01060271414668	1.85285603131294	6.31155918012984
H	-2.42208468421956	0.68410781442338	7.51940909171308
C	-1.81443901928278	0.24826512182262	5.45683190703799
H	-1.34260076823084	-0.66931737632348	5.83745963431791
H	-2.67432569492247	-0.02926609722697	4.84443918977620
C	-1.36421800494241	3.16778126285610	2.37753403922992
H	-1.96571823140930	3.72624497661913	1.65370006894818
H	-1.17068242440192	3.80496256541496	3.24233512671636
H	-1.94886604414562	2.30893012583724	2.71159189258698
C	-0.37138961125778	1.83130215772855	0.53279841722079
H	-0.83455266786764	0.89319591348966	0.84527734446564
H	0.51096328388583	1.60030929635161	-0.06348288436639
H	-1.08373716408569	2.33993015886931	-0.12439511452956
C	3.26111969903696	3.78702774582209	8.49015978085635
H	3.41179205653920	4.86431855881288	8.41921064461046
H	3.71419428799093	3.31991281773836	7.61905564761538
H	3.79128644018420	3.43661689500644	9.38176727614988
C	1.27073149757919	3.92779426675906	9.95711012660376
H	1.76865871545630	3.38531216425396	10.76714478318380
H	0.19560907455617	3.79023042652065	10.08536558931359
H	1.50029862848306	4.98540536370083	10.08867102055858
C	1.71834344407137	1.87123195941294	8.61544575905996
H	2.00308821398227	1.47621127460248	7.63700879672682

H	0.72058674811963	1.49873080135521	8.85339254076937
H	2.40981857652830	1.48193081880193	9.36897999949587
C	0.60779692907986	4.02427937204847	1.17182509487613
H	-0.07585099225193	4.49282916786797	0.45599000558379
H	1.54869483903109	3.82637828755954	0.65975414890718
H	0.80457250131320	4.73395406915228	1.97720635697217
P	0.77594452746966	3.93199307401900	7.11792107593993
C	2.40378424623141	0.92593870331952	2.28030763444549
C	3.17262977805782	1.71412562103411	1.22506780055929
H	2.58961860228429	1.86646354188461	0.31544811356909
H	4.06456111460451	1.14146818680116	0.95053053038087
H	3.49109713785312	2.68056802341787	1.62161684186572
C	3.35508756414320	0.60794986284170	3.43334223597600
H	3.79972849020128	1.52494544207950	3.82127133019427
H	4.14929097920578	-0.04985422270168	3.06470949509919
H	2.84949652974521	0.10565711958128	4.26269489301198
C	1.92700433420960	-0.38621711830913	1.65419317747843
H	1.50807472877492	-1.07818811918171	2.38539436926899
H	2.79482171931115	-0.88320784959942	1.21011399246127
H	1.19667130609575	-0.23815756546849	0.86002722983966
C	0.30934487311043	5.76160281930587	7.15571827669927
C	-0.51297913893909	6.01312350826615	5.88814939018867
H	-1.43294447716751	5.42445319905864	5.86011033689409
H	0.06640129996325	5.77024207807190	4.99588118131384
H	-0.79374542600308	7.07150017749575	5.86269950794047
C	-0.52570545695474	6.16335261819682	8.36998814816260
H	-0.87184047535601	7.19108646526947	8.22204307547992
H	0.04030161921880	6.14175547641876	9.29855401344526
H	-1.41391028933889	5.54137787257758	8.49442124758599
C	1.55098928341345	6.64435038539522	7.08227348853449
H	2.11426847980825	6.46087110468846	6.16622519531367
H	2.20708256331378	6.51617705279737	7.94450874567155
H	1.23345505350572	7.69173251882393	7.06641480467648
H	3.19931796940714	4.87458678052360	5.99599755543615
H	4.08823448342257	3.37692637064921	5.54256779876612

TSB_b (S = 0)

Eh = -2102.917227060384

Ir	0.39340705764671	0.23246872209299	-0.11722894133527
P	0.15790226357124	-1.01596426157915	-2.04734657348013
P	1.86352518496778	1.60701289788246	-1.39307517833096
C	-1.02252476618830	-0.96625762312313	0.43927052602914

N	-1.76040225808643	-0.90787583641413	1.60041939356724
N	-1.61038723422703	-1.93074599671654	-0.32693181486808
C	-1.70765228191327	0.31807813636545	2.33663888019601
H	-1.85126905244726	0.14428263096210	3.40558667603713
H	-2.48686665991607	1.01358702801913	1.99699453699303
C	0.95530303945647	0.45347571444800	3.44722764130736
C	-0.87947224228413	-2.43820230369949	-1.44011354071343
H	-1.55301949912547	-2.81514563675280	-2.21347377249646
H	-0.20780431677552	-3.25269689510388	-1.14215559398392
C	2.78872308084835	1.35163742677285	0.00791462872556
C	-0.92806947844415	-0.27549346727617	-3.40013896832405
C	-2.99626681934798	-1.65444659142900	1.46686794687009
H	-3.81192112878002	-1.00501826726148	1.11439708096674
H	-3.29812876271657	-2.11280386018893	2.41031260983094
C	-2.60921760445253	-2.67456418364479	0.40489116451642
H	-2.18289832532998	-3.58171595331094	0.85793445890236
H	-3.44747055964271	-2.96479989882554	-0.23220708639035
C	-2.27953718897573	0.02188031158629	-2.74552493746720
H	-2.89748131545962	0.57436022624312	-3.46014293184137
H	-2.14621274682224	0.63156454817530	-1.84959461769343
H	-2.81851461250601	-0.88577715210984	-2.46783033617888
C	-1.14944859207771	-1.18713401000577	-4.60128669667507
H	-1.53211305563838	-2.17150764514957	-4.32205581155769
H	-0.24167457292209	-1.32219512955920	-5.18940047814122
H	-1.89421061930360	-0.72444522887243	-5.25682189772623
C	2.44762436971396	0.72747707802388	3.28773283870236
H	2.68528121460183	1.78869845793827	3.20535745287248
H	2.84829836777468	0.21447618780933	2.41469530142961
H	2.96212023091309	0.34295678046337	4.17441228469456
C	0.49709572089457	1.01016744504382	4.79060689173326
H	0.98629406158293	0.44273700133527	5.58885677933506
H	-0.58030695012203	0.92542755158604	4.94397816066935
H	0.78217475360737	2.05523641211561	4.91700432470013
C	0.78563612560987	-1.06835769158053	3.44822810567290
H	1.04284579317722	-1.48499189326613	2.47205242569618
H	-0.22992340661084	-1.38057882973111	3.69414375243079
H	1.45875027418833	-1.49115826764194	4.20072830776909
C	-0.34590586435651	1.05451712146341	-3.86892932283861
H	-1.02091726491466	1.48667476360925	-4.61502174520455
H	0.63922328848144	0.95144920797626	-4.32177203005239
H	-0.26207115784258	1.75260412888809	-3.03539061696912
P	-0.05240930883631	1.07737470674385	1.96353846036339

C	1.62828880375874	-1.94250651993738	-2.78209442245374
C	2.40558850564344	-1.06019495254090	-3.75363898555709
H	1.85997577024645	-0.89315325743893	-4.68384719056929
H	3.34316096414498	-1.56456513140708	-4.00895759738169
H	2.64752324658849	-0.09140563450345	-3.30617739769070
C	2.53359223453568	-2.27427661538507	-1.59466228796768
H	2.88134406957119	-1.36647904117334	-1.10156420587440
H	3.39780803279924	-2.83933016006195	-1.95939102523789
H	2.02289600195206	-2.88176924845198	-0.84348752589037
C	1.24564880497538	-3.24434921093755	-3.48573501351244
H	0.76279089121161	-3.95941239489840	-2.81750171895161
H	2.16251362454060	-3.71807904351639	-3.85029939120817
H	0.59702819281277	-3.08595065009023	-4.34515877938080
C	-0.43844815693957	2.91713291382123	2.09761843794975
C	-1.19710987090729	3.28448904838133	0.82058134586613
H	-2.15237092161652	2.75965389638561	0.74441115139687
H	-0.60999000945329	3.04061090751688	-0.06552154362161
H	-1.40696914884725	4.35921770613117	0.83833352307617
C	-1.30208183017302	3.30018916493094	3.29737188463486
H	-1.56820406306075	4.35809272094211	3.20635107861898
H	-0.79001531230237	3.17481706505243	4.24836164902519
H	-2.23729613917261	2.73773268429497	3.33377426154778
C	0.86516168045592	3.70912741458115	2.12257451066604
H	1.48974452268512	3.47299181111737	1.25864830648312
H	1.43561110680895	3.53592829366842	3.03688800456655
H	0.63282748983375	4.77779722273279	2.07926770647254
H	2.49856281549951	1.68545540381193	0.99251931828045
H	3.80231151343871	0.94196774467268	-0.02201183102974

TSC (*S* = 0)

Eh = -2102.926736223854

Ir	0.99268943781265	3.20050063731011	4.98574271039588
P	0.85760680143571	1.93219365120931	3.08992111128619
P	2.63159287763537	4.58989919605563	4.36083848053621
C	-0.40262620497298	1.99713662677328	5.52417504063600
N	-1.00929941374926	1.93588848571817	6.75784369602648
N	-0.99426409848340	1.02165015326049	4.75436812136847
C	-0.94447901480322	3.10576660412522	7.57955935463385
H	-0.93851121850120	2.84560674746151	8.64110926638759
H	-1.80409881189493	3.76332484069229	7.39530742319674
C	1.85186091698197	3.51751011103687	8.39109015306343
C	-0.25343505655394	0.53992046179707	3.62884562735506

H	-0.91796311531902	0.21114143287434	2.82588745774854
H	0.38362541946893	-0.30801891562698	3.91309360935080
C	3.84142245769847	5.60867167080156	3.89541182550042
C	-0.08354420551337	2.72336530202531	1.66341367587682
C	-2.18025846532243	1.08594643826168	6.71293197712363
H	-3.08502233191626	1.66512857926935	6.47298099311712
H	-2.33976021140156	0.56396596584886	7.65829233003906
C	-1.81021611741428	0.14842120476948	5.57146324795640
H	-1.22957647990718	-0.71171322399435	5.93814266927258
H	-2.67960782298799	-0.22287990570868	5.02571193449451
C	-1.46782462033248	3.05558345770214	2.22623478620512
H	-2.01929811284172	3.63403550376537	1.47872069258033
H	-1.37857335475440	3.64848011468131	3.13905532047806
H	-2.05003215997333	2.16064902248158	2.45491634132350
C	-0.23957030979932	1.83750578255450	0.43443952305818
H	-0.66194600998558	0.85813403496141	0.67234292876618
H	0.70597309869416	1.68940802315563	-0.08822187335751
H	-0.92428611738582	2.32494199603643	-0.26695133262016
C	3.24144618282580	3.99937514551776	7.98463229300230
H	3.33818095180641	5.08400224972145	8.02460683320809
H	3.48391313214853	3.67668586532516	6.96981817137448
H	3.97636517144477	3.57223458431017	8.67432596420356
C	1.52892374141507	3.99954274400965	9.79941620883574
H	2.21868325021739	3.52178524974113	10.50247858177621
H	0.51472025603980	3.74152516895464	10.11431908206728
H	1.65929732732120	5.07743488097613	9.90221801322170
C	1.86619058547291	1.98696122364119	8.36802422032686
H	2.06638511996579	1.62100631888995	7.35862008407409
H	0.92282897581162	1.55387163213293	8.70660942987514
H	2.65755865160721	1.63506193047973	9.03699630526375
C	0.58811644557258	4.03843451892711	1.27660025065075
H	-0.04841670998165	4.55848706662436	0.55366791409033
H	1.56510386995805	3.89292615544593	0.81644423001960
H	0.72147198798849	4.67838458094518	2.15147867377571
P	0.59690778176531	4.02012712604188	7.07947127335179
C	2.38391149457782	1.01205217003329	2.49218768855968
C	3.26825224204222	1.95625620001721	1.68431697532371
H	2.83800979192287	2.18752607694625	0.70894079116145
H	4.23548304112870	1.47418050291244	1.51157159227895
H	3.45431427293976	2.89102535967350	2.21926066432005
C	3.14672646390431	0.59594527273104	3.75098509175182
H	3.43531948309486	1.46884537648409	4.33746653005473

H	4.04405835108030	0.04477438703870	3.45002680412664
H	2.55371359663148	-0.05462632988911	4.39885156250208
C	2.07267651273485	-0.23792444573727	1.67145090332738
H	1.47348960764536	-0.96089302570180	2.22845450772089
H	3.01627478388846	-0.73228667427785	1.41992958677357
H	1.56080257355182	-0.01706431831503	0.73702016987368
C	0.07350580731744	5.81572850981315	7.26214734893494
C	-0.79444826798785	6.12797468667656	6.04140353690723
H	-1.67830959423301	5.48778320170496	5.98482271401425
H	-0.23014382825577	5.99025619929388	5.11839516548258
H	-1.13399808921374	7.16696709424747	6.11038823784760
C	-0.73295960537975	6.10534427618141	8.52627386771339
H	-1.04337265487449	7.15481164472657	8.50732583099926
H	-0.16103562042427	5.95022337286191	9.43890529014243
H	-1.64285031481006	5.50487245308209	8.58543609031190
C	1.30506228734939	6.71441631308371	7.21530309377286
H	1.94073389685806	6.47921035292581	6.35788552375319
H	1.89961298244177	6.64266680578365	8.12681227914719
H	0.98125103459560	7.75480907594653	7.11374346661659
H	4.88915368023989	5.37152746852766	4.05978698140418
H	3.63909959394110	6.55829755224588	3.40736808426081

TSD (*S* = 0)

Eh = -2102.936318391197

Ir	1.17026226019065	3.10452824444770	4.99404695299010
P	0.94899735227949	1.88872824887129	3.06967136373654
P	3.20826424693046	4.11083518098773	4.18079388631910
C	-0.32377692952525	1.94821984727229	5.51786120305299
N	-0.94921212657541	1.93155123733388	6.72153932840231
N	-0.90256750984242	0.98492736771164	4.75787326855180
C	-0.84115677094399	3.08187134695658	7.56799110513982
H	-0.78187339076251	2.78945644245468	8.61864945719219
H	-1.72314089950543	3.72124325308492	7.45243371996351
C	1.85949755656959	3.66232587539393	8.45269248276331
C	-0.21787234273911	0.52592052050379	3.58710583528868
H	-0.92435654300137	0.26445913121810	2.79465746897727
H	0.37962921158532	-0.36441798930403	3.81418293019428
C	3.88226131042519	5.40642764499291	4.99559759196187
C	-0.01489924210136	2.80894065090272	1.74290186528282
C	-2.11618584746105	1.06938163828122	6.70890231272639
H	-3.02698722606147	1.65429152103515	6.51801566974603

H	-2.23926138328371	0.53580789520342	7.65295753778362
C	-1.79026250187067	0.14505897755944	5.53956949756950
H	-1.26926758974960	-0.76196356471791	5.87647593255297
H	-2.67359742357351	-0.15293629596724	4.97211165939575
C	-1.35843937042972	3.18511075060354	2.37102322120572
H	-1.90291200968975	3.83015585269637	1.67434344526523
H	-1.20968861024204	3.72333608035115	3.30929137543691
H	-1.98108959866410	2.31080345911797	2.57275009360024
C	-0.27436376724813	2.02013146460797	0.46594625945732
H	-0.78199075259947	1.07190981695463	0.65995493537505
H	0.64049612788310	1.81466053474095	-0.08973241685205
H	-0.92698805618134	2.60885313214456	-0.18707992713495
C	3.23614042645005	4.24424099161427	8.15805252901452
H	3.24258116394781	5.33437001257723	8.19173031194646
H	3.59758741997995	3.93579748732538	7.17697814770905
H	3.94005298376843	3.88892939531244	8.91759393912850
C	1.39189765037150	4.13576366726534	9.82259533589599
H	2.04410605399377	3.70290487646599	10.58801768841330
H	0.36918113422948	3.83287514483016	10.05687029080820
H	1.45894089593204	5.21969664906400	9.91797246659920
C	1.99385242470902	2.13618986926804	8.44862615727449
H	2.28739429432942	1.77822345251688	7.45845898601870
H	1.06758521893373	1.63132975122549	8.73084751346344
H	2.76407538930637	1.84901015036470	9.17102287555216
C	0.73298881698052	4.10300919248864	1.42449314560972
H	0.13849189595306	4.69516199863218	0.72093478995694
H	1.70931955858696	3.92653458930438	0.97500593012454
H	0.88751445789282	4.69036726409010	2.33181826943966
P	0.68758850105136	4.02884839710022	7.02451149731060
C	2.35006848072193	0.88136047011139	2.31929423785997
C	3.22204511541475	1.75391681087663	1.41908988596422
H	2.71291141693192	2.02764706601248	0.49392935692820
H	4.11437041271148	1.18227602409176	1.14293760877954
H	3.54919936049832	2.65962171371859	1.93596402783062
C	3.19288036682164	0.42416969924429	3.51214353260128
H	3.61395094545771	1.27884442008555	4.04320389824412
H	4.01349323333864	-0.20233926091247	3.14739246241994
H	2.61331655031515	-0.16507807751106	4.22781937121512
C	1.89246966957865	-0.34862130200342	1.53644612641886
H	1.35635021347498	-1.06900379924300	2.15699037004491
H	2.78111918733766	-0.85960063999569	1.15211407250529
H	1.26524844127443	-0.09917989328859	0.68192390524187

C	0.00271068850231	5.78556519433049	7.05485701707614
C	-0.93392537810896	5.86307371240412	5.84599833540966
H	-1.80279794336276	5.20898092689722	5.94767643252062
H	-0.40664118977753	5.58203078269410	4.93176404453401
H	-1.30135641756774	6.88966890998954	5.74804487721934
C	-0.77391683604954	6.15619499039828	8.31541317484370
H	-1.26231026909371	7.12214599569436	8.15102649311839
H	-0.12834227784391	6.26343715935322	9.18509294725687
H	-1.55824957723751	5.43798714669541	8.55995308552467
C	1.12260921895174	6.79365218430164	6.83832372581784
H	1.65863968944544	6.59081773597090	5.91117570586568
H	1.83457467635457	6.80331042299533	7.66508368988009
H	0.68905420969190	7.79614342323507	6.76555449817951
H	4.82567695328231	5.81188815750246	4.63326080239412
H	3.49061959870729	5.91092487146598	5.87232138409475

10 References

(1) Kumar, A.; Gupta, R.; Mani, G. PCP Pincer Carbene Nickel(II) Chloride, Hydride, and Thiolate Complexes: Hydrosilylation of Aldehyde, Ketone, and Nitroarene by the Thiolate Complex. *Organometallics* **2023**, *42*, 732-744.

(2) Suter, R.; Benkő, Z.; Bispinghoff, M.; Grützmacher, H. Annulated 1,3,4-Azadiphospholides: Heterocycles with Widely Tunable Optical Properties. *Angew. Chem. Int. Ed.* **2017**, *56*, 11226-11231.

(3) Transue, W. J.; Yang, J.; Nava, M.; Sergeyev, I. V.; Barnum, T. J.; McCarthy, M. C.; Cummins, C. C. Synthetic and Spectroscopic Investigations Enabled by Modular Synthesis of Molecular Phosphaalkyne Precursors. *J. Am. Chem. Soc.* **2018**, *140*, 17985-17991.

(4) Mallion, K. B.; Mann, F. G. 1095. The conditions determining the quaternisation of tertiary phosphines by methyl 2,4-dinitrobenzenesulphonate. *J. Chem. Soc.* **1964**, 5716-5725.

(5) Leroy, J.; Bensoam, J.; Humiliere, M.; Wakselman, C.; Mathey, F. Action du methyltri(n-butyl)fluorophosphorane sur les halogenures et les sulfonates d'alkyle. *Tetrahedron* **1980**, *36*, 1931-1936.

(6) *CrysAlis Pro*; Agilient Technologies ltd: 2014. (accessed 2025).

(7) Sheldrick, G. M. SHELXT - integrated space-group and crystal-structure determination. *Acta Crystallogr A Found Adv* **2015**, *71*, 3-8.

(8) Sheldrick, G. Crystal structure refinement with SHELXL. *Acta Cryst. C.* **2015**, *71*, 3-8.

(9) Dolomanov, O. V.; Bourhis, L. J.; Gildea, R. J.; Howard, J. A. K.; Puschmann, H. OLEX2: a complete structure solution, refinement and analysis program. *J. Appl. Crystallogr.* **2009**, *42*, 339-341.

(10) Fronza, G.; Bravo, P.; Ticozzi, C. Carbon-13 nuclear magnetic resonance studies of some phosphonium, arsonium, sulfonium and pyridinium keto-stabilized salts, and ylides and of their palladium(II) complexes. *J. Organomet. Chem.* **1978**, *157*, 299-310.

(11) Macur, S.; Farmer, B. T.; Brown, L. R. An improved method for the determination of cross-relaxation rates from NOE data. *J. Magn. Reson.* **1986**, *70*, 493-499.

(12) Neese, F. The ORCA program system. *WIREs Comput. Mol. Sci.* **2012**, *2*, 73-78.

(13) Neese, F. Software update: the ORCA program system, version 4.0. *WIREs Comput. Mol. Sci.* **2018**, *8*, e1327.

(14) Neese, F. Software update: The ORCA program system—Version 5.0. *WIREs Comput. Mol. Sci.* **2022**, *12*, e1606.

(15) Adamo, C.; Barone, V. Toward reliable density functional methods without adjustable parameters: The PBE0 model. *J. Chem. Phys.* **1999**, *110,* 6158-6170.

(16) Weigend, F. Accurate Coulomb-fitting basis sets for H to Rn. *Phys. Chem. Chem. Phys.* **2006**, *8*, 1057-1065.

(17) Weigend, F.; Ahlrichs, R. Balanced basis sets of split valence, triple zeta valence and quadruple zeta valence quality for H to Rn: Design and assessment of accuracy. *Phys. Chem. Chem. Phys.* **2005**, *7*, 3297-3305.

(18) Neese, F.; Wennmohs, F.; Hansen, A.; Becker, U. Efficient, approximate and parallel Hartree–Fock and hybrid DFT calculations. A 'chain-of-spheres' algorithm for the Hartree–Fock exchange. *Chem. Phys.* **2009**, *356*, 98-109.
(19) Neese, F. The SHARK integral generation and digestion system. *J. Comput. Chem.* **2023**, *44*, 381-396.
(20) Grimme, S.; Antony, J.; Ehrlich, S.; Krieg, H. A consistent and accurate ab initio parametrization of density functional dispersion correction (DFT-D) for the 94 elements H-Pu. *J. Chem. Phys.* **2010**, *132*, 154104.
(21) Grimme, S.; Ehrlich, S.; Goerigk, L. Effect of the damping function in dispersion corrected density functional theory. *J. Comput. Chem.* **2011**, *32*, 1456-1465.
(22) Tao, J.; Perdew, J. P.; Staroverov, V. N.; Scuseria, G. E. Climbing the Density Functional Ladder: Nonempirical Meta--Generalized Gradient Approximation Designed for Molecules and Solids. *Phys. Rev. Lett.* **2003**, *91*, 146401.
(23) Staroverov, V. N.; Scuseria, G. E.; Tao, J.; Perdew, J. P. Comparative assessment of a new nonempirical density functional: Molecules and hydrogen-bonded complexes. *J. Chem. Phys.* **2003**, *119*, 12129-12137.
(24) Staroverov, V. N.; Scuseria, G. E.; Tao, J.; Perdew, J. P. Erratum: "Comparative assessment of a new nonempirical density functional: Molecules and hydrogen-bonded complexes" [J. Chem. Phys. 119, 12129 (2003)]. *J. Chem. Phys.* **2004**, *121*, 11507-11507.
(25) Marenich, A. V.; Cramer, C. J.; Truhlar, D. G. Universal Solvation Model Based on Solute Electron Density and on a Continuum Model of the Solvent Defined by the Bulk Dielectric Constant and Atomic Surface Tensions. *J. Phys. Chem. B* **2009**, *113*, 6378-6396.
(26) Pascual-Ahuir, J. L.; Silla, E. GEPOL: An improved description of molecular surfaces. I. Building the spherical surface set. *J. Comput. Chem.* **1990**, *11*, 1047-1060.
(27) Silla, E.; Tuñón, I.; Pascual-Ahuir, J. L. GEPOL: An improved description of molecular surfaces II. Computing the molecular area and volume. *J. Comput. Chem.* **1991**, *12*, 1077-1088.
(28) Pascual-ahuir, J. L.; Silla, E.; Tuñon, I. GEPOL: An improved description of molecular surfaces. III. A new algorithm for the computation of a solvent-excluding surface. *J. Comput. Chem.* **1994**, *15*, 1127-1138.
(29) Neese, F. Importance of Direct Spin−Spin Coupling and Spin-Flip Excitations for the Zero-Field Splittings of Transition Metal Complexes: A Case Study. *J. Am. Chem. Soc.* **2006**, *128,* 10213-10222.
(30) Szabó, P. B.; Csóka, J.; Kállay, M.; Nagy, P. R. Linear-Scaling Open-Shell MP2 Approach: Algorithm, Benchmarks, and Large-Scale Applications. *J Chem Theory Comput* **2021**, *17,* 2886-2905.
(31) Lu, T.; Chen, F. Multiwfn: A multifunctional wavefunction analyzer. *J. Comput. Chem.* **2012**, *33*, 580-592.
(32) Ásgeirsson, V.; Birgisson, B. O.; Bjornsson, R.; Becker, U.; Neese, F.; Riplinger, C.; Jónsson, H. Nudged Elastic Band Method for Molecular Reactions Using Energy-Weighted Springs Combined with Eigenvector Following. *J Chem Theory Comput* **2021**, *17*, 4929-4945.
